# Design and Synthesis of Carbothioamide/Carboxamide-Based Pyrazoline Analogs as Potential Anticancer Agents: Apoptosis, Molecular Docking, ADME Assay, and DNA Binding Studies

**DOI:** 10.1021/acsomega.2c02033

**Published:** 2022-06-23

**Authors:** Manish Rana, Md Imam Faizan, Sajad Hussain Dar, Tanveer Ahmad, Rahisuddin

**Affiliations:** † Department of Chemistry, Jamia Millia Islamia, New Delhi 110025, India; ‡ Multidisciplinary Centre for Advanced Research & Studies, Jamia Millia Islamia, New Delhi 110025, India

## Abstract

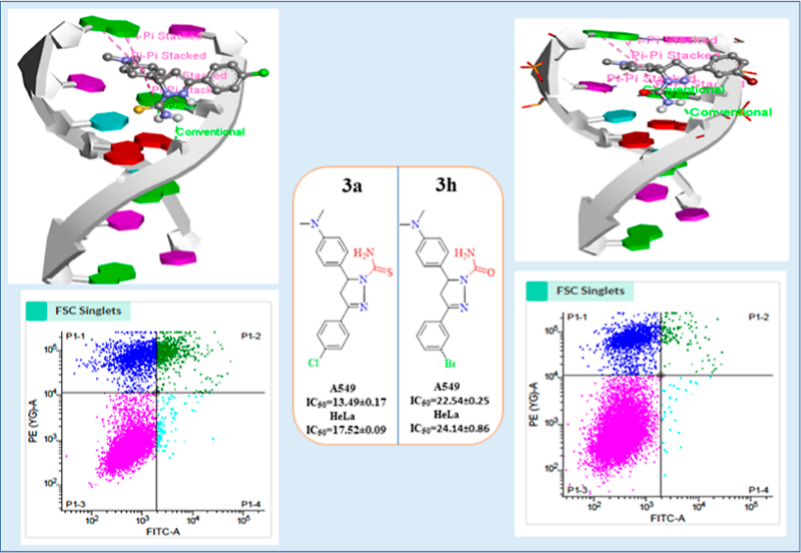

To discover anticancer drugs with novel structures and expand our research scope, pyrazoline derivatives (**3a**–**3l**) were designed and synthesized through cyclization of chalcones with thiosemicarbazide/semicarbazide in CH_3_COOH as a solvent. All newly synthesized pyrazoline derivatives were fully characterized using several spectroscopic experiments such as ^1^H, ^13^C NMR, FT-IR spectroscopy, and mass analysis. By **HPLC**, the purity of all analogs was found above **95%** and both lead compounds (**3a** and **3h**) were also validated by **HRMS**. Anticancer activity of synthesized pyrazoline derivatives (**3a**–**3l**) was investigated by the MTT assay against the human lung cancer cell (A549), human cervical cancer cell (HeLa), and human primary normal lung cells (HFL-1). Staurosporine (STS) was used as a standard drug. The anticancer results showed that two potent analogs **3a** and **3h** exhibit excellent activity against **A549** (IC_50_ = 13.49 ± 0.17 and 22.54 ± 0.25 μM) and **HeLa** cells (IC_50_ = 17.52 ± 0.09 and 24.14 ± 0.86 μM) and low toxicity against the HFL-1 (IC_50_ = 114.50 ± 0.01 and 173.20 ± 10 μM). The flow cytometry was further used to confirm the anticancer activity of potent derivatives against the A549 cancer cell line. DNA binding interaction of anticancer agents **3a** and **3h** with Ct-DNA has been carried out by absorption, fluorescence, EtBr (dye displacement assay), circular dichroism, cyclic voltammetry and time-resolved fluorescence, which showed noncovalent binding mode of interaction. Anticancer activity of both lead compounds (**3a** and **3h**) may be attributed to DNA binding. The evaluation of the antioxidant potential of pyrazoline analogs 3a and 3h by 2,2-diphenyl-1-picrylhydrazyl free radical showed promising antioxidant activity with IC_50_ values of 0.132 ± 0.012 and 0.215 ± 0.025 μg/mL, respectively. In silico molecular docking of pyrazoline derivatives was also performed using autodock vina software against the DNA hexamer with PDB ID: 1Z3F and ADMET properties to explore their best hits.

## 1 Introduction

**Figure 1:**
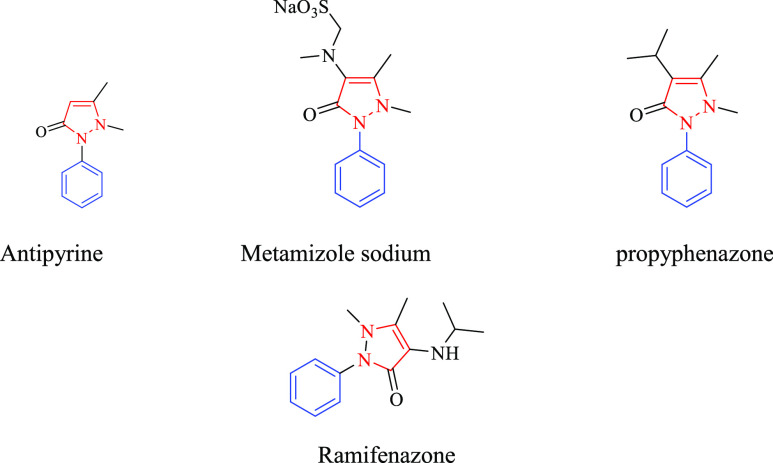
Some available bioactive drugs of pyrazoline containing scaffold.

**Figure 2:**
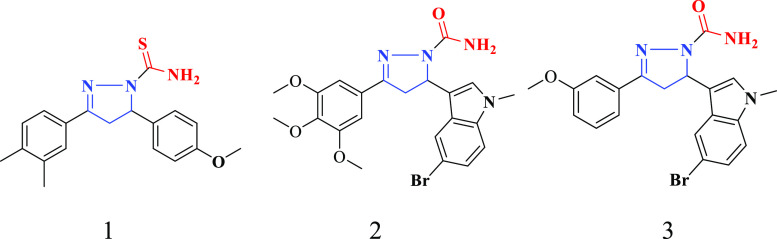
Reported carbothioamide/carboxamide-based pyrazoline derivatives.

**Figure 3:**
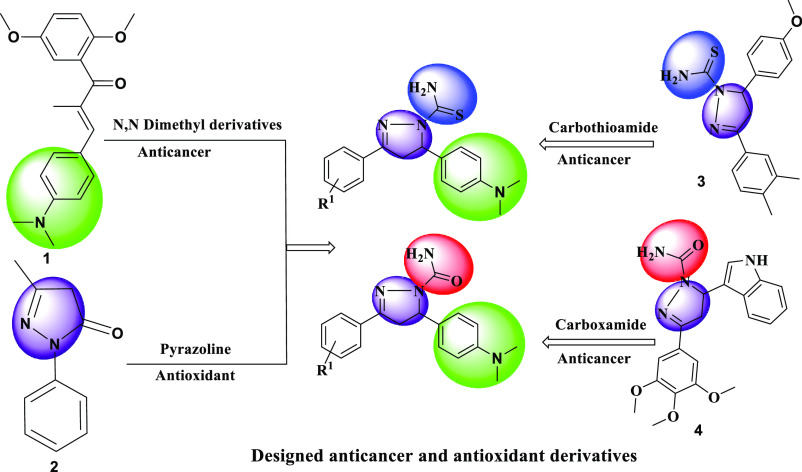
Design strategy of new pyrazoline compounds.

Cancer is one of the most critical health issues as well as the leading cause of mortality in the world. Among all types of cancer, lung and cervical cancer are the leading causes of cancer death. However, in the twenty-first century, effective malignant tumor therapy is a problem, and new and less hazardous anticancer medicines with a broader range of tumor cell cytotoxicity may be necessary.^1−3^ Nearly all kinds of cancer belong to a wide category of diseases in which abnormal cells develop out of control and invade nearby organs. The three most frequent treatments are general surgery, chemotherapy, and radiation. However, in most malignant tumor types, there may not be a treatment that is completely successful.^4−7^ There are many drugs currently used for cancer therapy worldwide, however, anticancer drugs have major shortcomings such as dose-limiting side effects, induced cellular resistance, intrinsic acquired resistance, acceptable specificity, reduced bioavailability, severe toxicity, uncomfortable, cost-intensive way of administration, and a spectrum of activity limited to narrow range of tumor types. Despite such limitations, researchers took efforts to develop anticancer agents for reducing these effects.^8,9^ In the last several years, the FDA has approved a large number of heterocyclic analogs as chemotherapeutic drugs.^10,11^ Pyrazoline is a pyrazole substructure found in just a few of them. In the field of drug design, pyrazoline (4,5-dihydropyrazoles) is one of the most prominent instances of a physiologically active five-membered ring. The N–N bond in the pyrazoline ring and its biological applications appear to be one of the most important factors. Natural compounds have fewer N–N bonds than living creatures because they are more difficult to form. Heterocyclic analogs such as 1*H*-pyrazole-1-carbothioamide/carboxamide are often used to design and develop physiologically active novel medications.^12−16^ The pyrazoline core showed the potential anticancer activity^17−21^, and 1*H*-pyrazole-1-carbothioamide types also exhibited biological activities such as antibacterial,^22^ antifungal,^23^ antiviral,^24^ antimalarial,^25^ antioxidant,^26^ anti-inflammatory,^27,28^^,^ and analgesic effects.^29−31^ Anticancer drugs have DNA as their primary intracellular target. In the present era, researchers are learning about the interaction of medications with DNA, and they believe that this interaction is responsible for DNA damage produced by malignant tumor cells’ inability to proliferate quickly.^32,33^ Medication and micro-molecules commonly employ noncovalent, intercalation, groove binding, and electrostatic binding to interact with DNA. In today’s market, there are numerous different substances with pyrazoline rings that have a variety of actions,^34,35^ that is, examples of adducible medicines include antipyrine, metamizole, propyphenazone, and ramifenazone (Figure 1). The carbothioamide/carboxamide-based pyrazoline analog (1) displayed potent cytotoxic activity against breast cancer cell line (MCF-7) with an IC_50_ value of 0.08 μM.^36^ Moreover, the pyrazoline derivatives (2 and 3) exhibited promising cytotoxicity against HeLa with IC_50_ values of 0.21 and 0.25 μM, respectively^37^ (Figure 2). For our present research work, the design strategy for the synthesis of pyrazoline derivatives is depicted in Figure 3. A549 cells are lung adenocarcinoma cells, and thus, an ideal cancer cell line to test our synthetic drug products. As lung cancer is one of the leading causes of mortality worldwide, we believe that our lead compounds **3a** and **3h** will be an ideal candidate for the treatment of lung cancer. To expand the anticancer activity of the drug products, we further chose Hela cells, which are well-established cell lines for testing the anticancer drugs and other therapeutic products. For us, the rationale to use Hela cells was to validate the findings of A549 in another cell line, and of a tissue of different origin besides lungs. Therefore, using more than one cell line and of different tumor origin provides a more comprehensive evaluation of the screened drug products. The apoptosis, DNA binding, molecular docking, ADMET assay, and antioxidant assay of the lead analogs were carried out.

## 2 Experimental Section

### 2.1 Materials and Methods

All reagents and solvents, TLC (precoated 60 F_254_Al sheets, Merck), melting point, IR, ^1^H and ^13^C NMR spectra, chemical shift values in ppm, mass spectral analysis and UV–visible, fluorescence, time-resolved fluorescence, dye displacement assay, and cyclic voltammetry to examine the DNA-drug interaction performed as described previously.^38^

### 2.2 Synthesis of Carbothioamide/Carboxamide Pyrazoline Derivatives (**3a**–**3l**)

**Scheme 1:**
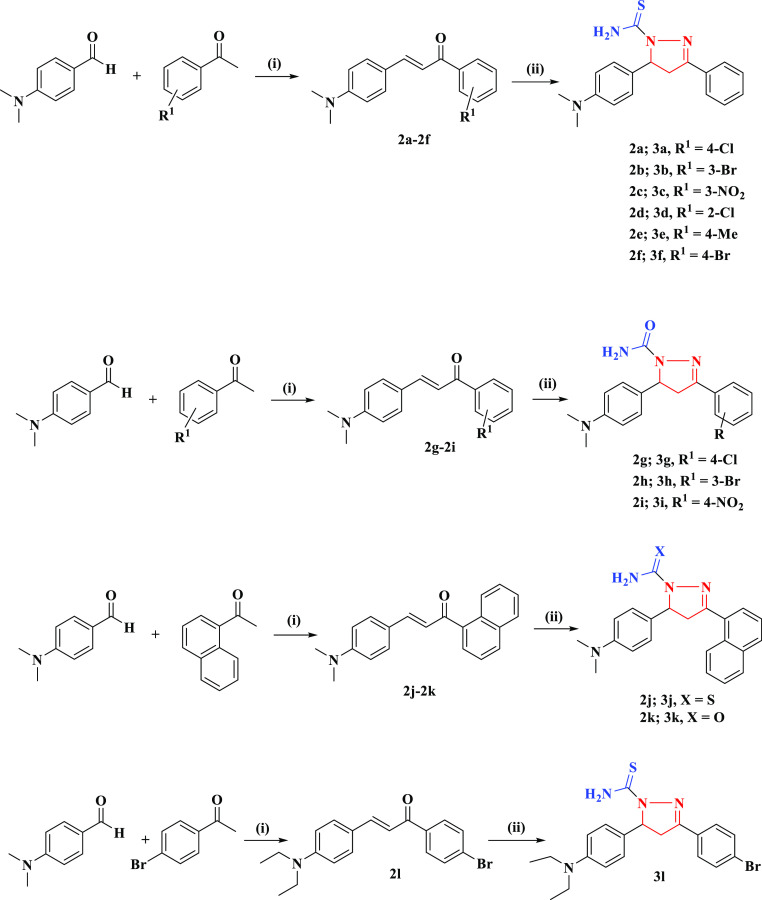
Preparation of Pyrazoline Derivatives (**3a**–**3l**) (i) NaOH (50%), absolute ethanol, stir; (ii) thiosemicarbazide/semicarbazide, reflux for 4–6 h.

As reported earlier,^39^ chalcone analogs (**2a**–**2l**) were synthesized in ethanol as solvent by the reaction of substituted aldehydes with acetophenone derivatives in the presence of a base (NaOH). 1 mmol chalcone derivatives (**2a**–**2f**, **2j**, and **3l**) were stirred with 1 mmol thiosemicarbazide in glacial acetic acid (5 mL) and heated under reflux conditions for 4–6 h. The same procedure was followed for chalcone derivatives (**2g**–**2i** and **2k**) with 1 mmol semicarbazide in acetic acid. The precipitates (**3a**–**3l**) were filtered, washed with water, dried, and recrystallized in chloroform. The synthesis of pyrazoline analogs is illustrated in Scheme 1.

Compound (**3a**) color/yield: light green/80%. Eluent: hexane/EtOAc = 3/1. mp 105 °C. IR (neat, ν_max_ cm^–1^): ν(NH_2_) 3263, 3442 ν(C=N) 1587 ν(C=S) 1337. ^1^H NMR (400 MHz, δ ppm, DMSO-*d*_6_): 7.79–7.81 (d, 2H), 7.48–7.50 (d, 2H), 6.98–7.00 (d, 2H), 6.65–6.67 (d, 2H), 6.45 (s, 2H), 5.31 (dd, *J*_AX_ = 4.6, *J*_BX_ = 11.8 Hz, 1H, C_5_-H_X_), 3.73 (dd, *J*_BX_ = 11.8, *J*_BA_ = 17.7 Hz, 1H, C_4_-H_B_), 3.02 (dd, *J*_AX_ = 4.6, *J*_AB_ = 17.7 Hz, 1H, C_4_-H_A_), 2.84 (6H, s, N(CH_3_)_2_). ^13^C NMR (75.47 MHz, δ ppm, CDCl_3_): 176.74, 155.03, 149.88, 132.09, 129.82, 129.62, 128.32, 126.50, 125.31, 112.78, 63.29, 42.92, 40.61 HRMS (*m*/*z*) calcd for C_18_H_19_N_4_S (M + Na^**+**^): 381.09; (M + Na^**+**^) found: 381.0905. HPLC: purity %: 98.91; compound (**3b**) color/yield: light pink/78%. Eluent: hexane/EtOAc = 3/1. mp 190 °C. IR (neat, ν_max_ cm^–1^): ν(NH_2_) 3245, 3422 ν(C=N) 1584 ν(C=S) 1354. ^1^H NMR (400 MHz, δ ppm, DMSO-*d*_6_): 7.75–7.77 (d, 2H), 7.59–7.61 (d, 2H), 6.88–6.90 (d, 2H), 6.58–6.60 (d, 2H), 7.90 (s, 2H), 5.74 (dd, *J*_AX_ = 4.6, *J*_BX_ = 11.8 Hz, 1H, C_5_-H_X_), 3.76 (dd, *J*_BX_ = 11.8, *J*_BA_ = 17.7 Hz, 1H, C_4_-H_B_), 3.03 (dd, *J*_AX_ = 4.6, *J*_AB_ = 17.7 Hz, 1H, C_4_-H_A_), 2.78 (s, 6H, N(CH_3_)_2_). ^13^C NMR (CDCl_3_, 75.47 MHz): 177.15, 159.89, 155.64, 150.26, 133.86, 133.19, 130.56, 129.94, 129.49, 126.67, 125.70, 123.25, 112.86, 63.56, 43.14, 40.72. MS (*m*/*z*) calcd for C_18_H_19_N_4_S (M + 2): 403.34; found: 405.16. HPLC: purity %: 96.83; compound (**3c**) color/yield: yellow/76%. Eluent: hexane/EtOAc = 3/1. mp 193 °C. IR (neat, ν_max_ cm^–1^): ν(NH_2_) 3259, 3433 ν(C=N) 1588 ν(C=S) 1334. ^1^H NMR (CDCl_3_, 300 MHz ppm): 7.52–7.56 (m, 4H), 7.11–7.13 (d, 2H), 6.67–6.69 (d, 2H), 5.29 (s, 2H), 5.46 (dd, *J*_AX_ = 4.6, *J*_BX_ = 17.8 Hz, 1H, C_5_-H_X_), 3.70 (dd, *J*_BX_ = 11.8, *J*_BA_ = 17.7 Hz, 1H, C_4_-H_B_), 3.14 (dd, *J*_AX_ = 4.6, *J*_AB_ = 11.8 Hz, 1H, C_4_-H_A_), 2.91 (s, 6H, N(CH_3_)_2_). ^13^C NMR (CDCl_3_, δ ppm, 75.47 MHz): 177.85, 156.14, 151.0, 133.20, 130.93, 130.74, 129.43, 127.61, 126.43, 113.89, 64.40, 44.03, 41.72. MS (*m*/*z*) calcd for C_18_H_19_N_5_S (M+1): 369.44; found: 370.18. HPLC: purity: 94.65; compound (**3d**) color/yield: light brown/78%. Eluent: hexane/EtOAc = 3/1. mp 175 °C. IR (neat, ν_max_ cm^–1^): ν(NH_2_) 3247, 3430 ν(C=N) 1576 ν(C=S) 1333. ^1^H NMR (CDCl_3_, 300 MHz, δ ppm): 7.54–7.61 (m, 4H), 7.08–7.11 (d, 2H), 6.69–6.72 (d, 2H), 6.09 (s, 2H), 5.97 (dd, *J*_AX_ = 4.6, *J*_BX_ = 17.8 Hz, 1H, C_5_-H_X_), 3.77 (dd, *J*_BX_ = 11.8, *J*_BA_ = 17.7 Hz, 1H, C_4_-H_B_), 3.19 (dd, *J*_AX_ = 4.6, *J*_AB_ = 11.8 Hz, 1H, C_4_-H_A_), 2.92 (s, 6H, N(CH_3_)_2_). ^13^C NMR (CDCl_3_, δ ppm, 75.47 MHz): 178.58, 169.83, 154.88, 149.74, 132.09, 129.82, 129.60, 128.32, 126.50, 125.32, 116.52, 112.77, 63.30, 42.92, 40.61. MS (*m*/*z*) calcd for C_18_H_19_N_4_S (M + 1): 358.89; found: 359.17. HPLC: purity %: 98.21; compound (**3e**) color/yield: light pink/81%. Eluent: hexane/EtOAc = 3/1. mp 128 °C. IR (neat, ν_max_ cm^–1^): ν(NH_2_) 3247, 3448 ν(C=N) 1576 ν(C=S) 1342. ^1^H NMR (400 MHz, δ ppm, DMSO-*d*_6_): 7.69–7.71 (d, 2H), 7.19–7.21 (d, 2H), 6.88–6.90 (d, 2H), 6.58–6.60 (d, 2H), 7.83 (s, 2H), 5.73 (dd, *J*_AX_ = 4.6, *J*_BX_ = 17.8 Hz, 1H, C_5_-H_X_), 3.74 (dd, *J*_BX_ = 11.8 *J*_BA_ = 17.7 Hz, 1H, C_4_-H_B_), 3.02 (dd, *J*_AX_ = 4.6, *J*_AB_ = 11.8 Hz, 1H, C_4_-H_A_), 2.78 (s, 6H, N(CH_3_)_2_), 2.28 (s, 3H). ^13^C NMR (CDCl_3_, δ ppm, 75.47 MHz): 176.39, 156.32, 149.83, 141.42, 129.90, 128.53, 128.06, 126.92, 126.56, 112.78, 111.74, 63.05, 55.43, 43.11, 40.63, 21.55. MS (*m*/*z*) calcd for C_19_H_22_N_4_S (M + 1):338.47; found: 339.20. HPLC: purity %: 98.61; compound (**3f**) color/yield: light yellow/77%. Eluent: hexane/EtOAc = 3/1. mp 190 °C. IR (neat, ν_max_ cm^–1^): ν(NH_2_) 3227, 3430 ν(C=N) 1576 ν(C=S) 1333. ^1^H NMR (400 MHz, δ ppm, DMSO-*d*_6_): 7.78–7.80 (d, 2H), 7.61–7.63 (d, 2H), 6.90–6.92 (d, 2H), 6.61–6.63 (d, 2H), 7.86 (s, 2H), 5.75 (dd, *J*_AX_ = 4.6 *J*_BX_ = 17.8 Hz, 1H, C_5_-H_X_), 3.78 (dd, *J*_BX_ = 11.8 *J*_BA_ = 17.7 Hz, 1H, C_4_-H_B_), 3.05 (dd, *J*_AX_ = 4.6, *J*_AB_ = 11.8 Hz, 1H, C_4_-H_A_), 2.81 (s, 6H, N(CH_3_)_2_). ^13^C NMR (CDCl_3_, δ ppm, 75.47 MHz): 177.27, 153.40, 150.10, 148.71, 136.95, 128.98, 128.58, 126.44, 124.08, 112.59, 63.70, 42.84, 40.47. MS (*m*/*z*) calcd for C_18_H_19_N_4_S (M+): 403.34; found: 403.09. HPLC: purity %: 98.21; compound (**3g**) color/yield: yellow/73%. Eluent: hexane/EtOAc = 3/1. mp 165 °C. IR (neat, ν_max_ cm^–1^): ν(NH_2_) 3276, 3442 ν(C=N) 1513 ν(C=S) 1342. ^1^H NMR (400 MHz, δ ppm, DMSO-*d*_6_): 7.79–7.81 (d, 2H), 7.48–7.50 (d, 2H), 6.98–7.00 (d, 2H), 6.65–6.67 (d, 2H), 6.45 (s, 2H), 5.31 (dd, *J*_AX_ = 4.6 *J*_BX_ = 17.8 Hz, 1H, C_5_-H_X_), 3.73 (dd, *J*_BX_ = 11.8, *J*_BA_ = 17.7 Hz, 1H, C_5_-H_X_), 3.02 (dd, *J*_AX_ = 4.6, *J*_AB_ = 11.8 Hz, 1H, C_4_-H_A_), 2.84 (s, 6H, N(CH_3_)_2_). ^13^C NMR (CDCl_3_, δ ppm, 75.47 MHz): 176.74, 155.03, 149.88, 132.09, 129.82, 129.62, 128.32, 126.50, 125.31, 112.78, 63.29, 42.92, 40.61. MS (*m*/*z*) calcd. for C_18_H_19_N_4_O (M + 1): 342.83; found: 343.10. HPLC: purity %: 95.00; compound (**3h**) color/yield: yellow/71%. Eluent: hexane/EtOAc = 3/1. mp 178 °C. IR (neat, ν_max_ cm^–1^): ν(NH_2_) 3269, 3491 ν(C=N) 1579 ν(C=S) 1342. ^1^H NMR (CDCl_3_, 300 MHz ppm): 7.53–7.60 (m, 4H), 7.08–7.11 (d, 2H), 6.67–6.70 (d, 2H), 6.07 (s, 2H), 5.96 (dd, *J*_AX_ = 4.6, *J*_BX_ = 17.8 Hz, 1H, C_5_-H_X_), 3.76 (dd, *J*_BX_ = 11.8, *J*_BA_ = 17.7 Hz, 1H, C_4_-H_B_), 3.18 (dd, *J*_AX_ = 4.6, *J*_AB_ = 11.8 Hz, 1H, C_4_-H_A_), 2.91 (s, 6H, N(CH_3_)_2_). ^13^C NMR (CDCl_3_, δ ppm, 75.47 MHz): 155.12, 150.77, 150.14, 131.88, 130.56, 130.13, 128.27, 127.82, 126.44, 124.13, 112.83, 59.90, 42.66, 40.63. HRMS (*m*/*z*) calcd for C_18_H_19_N_4_O (M + Na^**+**^):409.06; (M + Na^**+**^): found: 409.0626. HPLC: purity %: 95.57; compound (**3i**) color/yield: yellow/79%. Eluent: hexane/EtOAc = 3/1. mp 130 °C. IR (neat, ν_max_ cm^–1^): ν(NH_2_) 3266, 3479 ν(C=N) 1591 ν(C=S) 1354. ^1^H NMR (400 MHz, δ ppm, DMSO-*d*_6_): 8.20–8.22 (d, 2H), 7.96–7.99 (d, 2H), 6.93–6.95 (d, 2H), 6.59–6.61 (d, 2H), 6.55 (s, 2H), 5.31 (dd, *J*_AX_ = 4.6, *J*_BX_ = 17.8 Hz, 1H, C_5_-H_X_), 3.73 (dd, *J*_BX_ = 11.8, *J*_BA_ = 17.7 Hz, 1H, C_4_-H_B_), 3.04 (dd, *J*_AX_ = 4.6, *J*_AB_ = 11.8 Hz, 1H, C_4_-H_A_), 2.79 (s, 6H, N(CH_3_)_2_). ^13^C NMR (CDCl_3_, 75.47 MHz): 154.86, 150.23, 149.45, 148.14, 137.64, 129.57, 126.92, 126.42, 123.99, 112.78, 60.43, 42.43, 40.55. MS (*m*/*z*) calcd for C_18_H_19_N_5_O_3_ (M + 1):353.38.; found: 354.15. HPLC: purity %: 98.51; compound (**3j**) color/yield: yellow/67%. Eluent: hexane/EtOAc = 3/1. mp 220 °C. IR (neat, ν_max_ cm^–1^): ν(NH_2_) 3257, 3451 ν(C=N) 1576 ν(C=S) 1351. ^1^H NMR (CDCl_3_, 300 MHz ppm): 9.24–9.27 (d, 2H), 7.99–8.05 (t, 2H), 7.52–7.79 (m, 4H), 7.02–7.05 (d, 2H), 6.65–6.67 (2H), 8.91 (s, 1H), 5.85 (dd, *J*_AX_ = 4.6, *J*_BX_ = 17.8 Hz, 1H, C_5_-H_X_), 4.12 (dd, *J*_BX_ = 11.8, *J*_BA_ = 17.7 Hz, 1H, C_4_-H_B_), 3.25 (dd, *J*_AX_ = 4.6, *J*_AB_ = 11.8 Hz, 1H, C_4_-H_A_), 2.83 (s, 6H, N(CH_3_)_2_). ^13^C NMR (CDCl_3_, δ ppm, 75.47 MHz): 176.56, 167.02, 156.63, 150.02, 134.01, 131.68, 131.00, 130.26, 129.93, 129.10, 128.51, 127.74, 127.45, 126.73, 125.65, 112.89, 61.76, 45.54, 40.80. MS (*m*/*z*) calcd for C_22_H_22_N_4_S (M + 1): 374.51; found: 375.26. HPLC: purity %: 95.61; compound (**3k**) color/yield: yellow/65%. Eluent: hexane/EtOAc = 3/1. mp 203 °C. IR (neat, ν_max_ cm^–1^): ν(NH_2_) 3266, 3488 ν(C=N) 1567 ν(C=S) 1364. ^1^H NMR (CDCl_3_, 300 MHz ppm): 9.27–9.29 (d, 2H), 7.97–8.00 (d, 2H), 7.50–7.76 (m, 4H), 7.06–7.09 (d, 2H), 6.67–6.70 (d, 2H), 6.49 (s, 2H), 5.32 (dd *J*_AX_ = 4.6, *J*_BX_ = 17.8 Hz, 1H, C_5_-H_X_), 3.73 (dd, *J*_BX_ = 11.8, *J*_BA_ = 17.7 Hz, 1H, C_4_-H_B_), 3.04 (dd, *J*_AX_ = 4.6, *J*_AB_ = 11.8 Hz, 1H, C_4_-H_A_), 2.84 (s, 6H, N(CH_3_)_2_). ^13^C NMR (CDCl_3_, δ ppm, 75.47 MHz): 154.85, 151.31, 149.56, 133.56, 131.45, 130.25, 129.87, 128.54, 128.37, 127.89, 127.69, 127.02, 126.25, 125.16, 112.61, 58.12, 44.85, 40.33. MS (*m*/*z*) calcd for C_22_H_22_N_4_O (M + 1): 358.45; found: 359.27. HPLC: purity %: 99.79; compound (**3l**) color/yield: light blue/85%. Eluent: hexane/EtOAc = 3/1. mp 208 °C. IR (neat, ν_max_ cm^–1^): ν(NH_2_) 3247, 3442 ν(C=N) 1574 ν(C=S) 1339. ^1^H NMR (400 MHz, δ ppm, DMSO-*d*_6_): 7.78–7.80 (d, 2H), 7.61–7.63 (d, 2H), 6.86–6.88 (d, 2H), 6.50–6.52 (d, 2H), 7.94 (s, 2H), 5.74 (dd, *J*_AX_ = 4.6, *J*_BX_ = 17.8 Hz, 1H, C_5_-H_X_), 3.76 (dd, *J*_BX_ = 11.8, *J*_BA_ = 17.7 Hz, 1H, C_4_-H_B_), 3.06 (dd, *J*_AX_ = 4.6, *J*_AB_ = 11.8 Hz, 1H, C_4_-H_A_), 3.23 (q, 4H), 1.00 (t, 6H). ^13^C NMR (CDCl_3_, δ ppm, 75.47 MHz): 176.66, 155.20, 147.14, 132.07, 129.85, 128.33, 127.75, 125.29, 111.73, 63.28, 44.39, 42.92, 12.57. MS (*m*/*z*) calcd for C_20_H_23_N_4_S (M + 1): 431.40; found: 432.12. HPLC: purity %: 98.51.

### 2.3 In Vitro Cytotoxicity Evaluation

The American type culture collection (ATCC, USA) provided human lung cancer cell line (A549), cervical cancer cell line (HeLa), and human primary normal lung cells (HFL-1) for this investigation. In a water-jacketed cell culture incubator, all of these cell types were cultured according to conventional culture conditions at 37 °C with 5% CO_2._ These cells were cultured in appropriate cell culture media. A549 cells were grown in DMEM media with 2 mM glutamine, HeLa cells were grown in Eagle’s minimum essential medium (EMEM) as recommended, whereas HFL-1 cells were also grown in F-12K. Fetal bovine serum 10% (FBS) was added to the entire medium. At a rate of 8000 cells were seeded in triplicate in a single 96-well plate for the MTT technique of cell death investigation. The plate was treated with an increasing concentration of pyrazoline derivatives (5, 10, 15, 20, 25, and 50 μM) and Staurosporine dissolved in DMSO. The MTT assay was used to determine whether the test substances have any effect on cell death after 24 h of treatment. After the treatment period was finished, the media was removed, and each well was gently washed three times with PBS to clear any remaining residue just before the experiment. We used 10 μL of MTT reagent (Sigma, USA, cat. no. 11465007001) in each well with a working stock concentration of 5 mg/mL, as previously described.^40,41^ The plates were incubated at 37 °C for 4 h after the MTT was added. We used DMSO to remove crystals after the incubation period was completed. A hybrid multi-mode plate reader (BioTek, USA) with a microplate reader was used to measure absorbance at 570 nm. The percentage inhibition was calculated using eq 1:
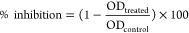
1where OD_treated_ is the mean optical density (OD) of the treated cells and ODcontrol is the OD of the vehicle control cells (negative control).

With all of the experimental drugs, we conducted the MTT assay three times. Prism8 software (GraphPad) was used to determine the IC_50_ values of the various drugs, which were then expressed as a drug concentration (μM).

### 2.4 Apoptosis Studies

We used flow cytometry analysis on A549 and HeLa cells for the quantitative apoptosis assay. As previously stated, cells were cultivated at 1.0 × 10^5^ cells/ml and plated in 24-well culture plates (Corning) for 24 h. Cells were cultured for further 24 h after being treated with 4 μm of target drugs **3a** and **3h**. For analysis, the medium was discarded and cells were washed briefly in PBS, and then, the trypsinization protocol was carried out. Furthermore, cells were suspended in PBS and stained with annexin in V-FITC/PI according to the supplier technique (Sigma, USA, catalogue number: APOAF-20TST). After adding the reagents, the cells were allowed to incubate for 30 min at room temperature before being analyzed in PBS. The analysis was conducted on a BD FACS Accuri (BD Biosciences, USA), and the results were analyzed using FlowJo software (BD Biosciences, USA).^42^

### 2.5 Molecular Docking Study

Investigators used molecular docking to determine which drugs exhibit anticancer effects, including the target area’s binding domain. Crystal structure B-DNA was downloaded from Protein Data Bank (http://www.pdb.org/, ID: 1Z3F). PDB file of drug structures **3a**–**3l** (as a flexible ligand) was obtained via Mervin software. The hexamer B-DNA was docked with pyrazoline analogs using the autodock vina software 4.0 version. Autodock was used to eliminate heteroatoms and water molecules around the duplex. Introduced to the specific receptor are polar hydrogen atoms, Kollman unified atom type charges, and gasteiger partial charges. For BNA, a grid box with 74 × 64 × 117 Å point spacing of 0.375 Å was used to reformate structure files into PDBQT formats. The number of generated algorithm runs and assessments were both limited to 100. To compare structural similarities, the most optimized model with the minimum binding energy was chosen (RMSD = 0.0). The Discovery studio visualizer was used to examine the docked DNA-drug complex image including hydrogen bonds, π–π stacking, and bond lengths.^43,44^

### 2.6 In Silico ADMET Assay

The drug’s likeness features of all heterocyclic analogs (**3a**–**3l**) were screened out to determine the drug-like behavior. The ADMET characteristics of proposed chemical compounds were computed using the free online Swiss ADMET program after 2D structures were converted to canonical SMILES representation. It is important in the drug development process because it provides free access to reliable models for physicochemical pharmacokinetics and drug-likeness property prediction.^45,46^

### 2.7 DNA Binding Studies

#### 2.7.1 Absorption Titration

The DNA interaction affinity of the test compound is determined using electronic absorption titration. First, in Tris-buffer solutions, the absorbance of pure DNA, compounds, and λ_max_ has been calculated. The absorbance proportion at fixed wavelengths 260 and 280 nm was 1.9:1, indicating that DNA was free from protein contamination. The intrinsic binding constant (*K*_b_) was utilized to evaluate pyrazoline DNA binding affinity by using eq 2. 2 mL of Tris-HCl/NaCl buffer (pH = 7.4) was used to dilute a 5 μL stock solution of pyrazoline derivatives (5 mg/2 mL in DMSO). The molar absorption coefficient (ε_260_) 6600 L mol^–1^ cm^–1^ was used to evaluate the concentration of DNA in the stock solution. The UV–visible spectrum of the compound–DNA mixture solution was obtained when an increasing concentration of Ct-DNA solution (10–100 μM) was added to the solution of the test compound (10 μM) at 25 °C. The mixture solution was allowed to incubate for 3 min before observations. Titration experiments were repeated three times (*n* = 3) to ensure consistent results. The binding affinity (*K*_b_) was calculated using eq 2:^47−49^
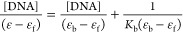
2where *A*_obsd_/[compound], the extinction coefficient of the complex in the bound form, and the extinction coefficient for the complex are represented by absorption coefficients, ε_a_, ε_b_, and ε_f_, respectively.

#### 2.7.2 Fluorescence Measurements

Drug interactions with Ct-DNA have been studied using fluorescence assays. The experiments were achieved by varying drug concentration from 10 to 80 μM while keeping the concentration of DNA (10 μM) constant. Before making emission observation, the synthesized compound was incubated for 3 min with mixing DNA, after which emission titration was collected in Tris-buffer (pH 7.4) excitation at wavelength 260 nm, and the quenching results were analyzed using the Stern–Volmer equation (eq 3):
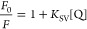
3Slit width has been kept constant at 10 nm during emission and excitation. The titration experiments were repeated three times (*n* = 3) to ensure consistent results.^50−52^

#### 2.7.3 Competitive Fluorescent Displacement Assays

Competitive fluorescence binding experiments utilizing ethidium bromide, a standard intercalation indicator, were used to evaluate the degree of molecular binding interaction mechanism of an active drug with Ct-DNA. The presence of compounds, which is highly effective in replacing EB from the binding site, might quench the rising fluorescence intensity. The observable changes in the EB–DNA adduct’s emission spectrum as a function of quencher addition are particularly valuable for researching compound–DNA interactions. Fluorescence measurements were done in Tris-buffer at a 515 nm excitation wavelength in the range of 530–700 nm. Slit widths for both emission and excitation were set at about 10 nm.^54,69^

#### 2.7.4 Circular Dichroism

This experimental technique is a simple and effective way to observe structural changes that take place as a result of DNA and drug interactions. These studies were carried out in the free DNA and presence of the compounds (10 μM) at room temperature in Tris buffer (pH = 7.4) in the wavelength range from 200 to 320 nm.^55^ At 50 nm/min scan rate, the spectrum was generated by averaged triple scans and eliminating buffer background.

#### 2.7.5 Electrochemical Measurements

Using this experiment, a drug–DNA binding interaction has also been investigated. The solution’s pH was maintained before analysis, and the entire analytical equipment was built at 25 °C with a constant potential of 100 mV s^–1^ scan rate and a voltage range of −1 to +1 V. The spectra of a test compound (60 μL) were recorded using a screen-printed electrode (SPE). These experiments were carried out in a 1:1 ferri/ferrocyanide solution ratio. Various concentrations of Ct-DNA (Tris-buffer) were used to further evaluate test compounds **3a** and **3h**. The working electrode is made of the test compound, whereas the counter and reference electrodes are made of gold-coated copper. Binding constants were obtained using eq 4:
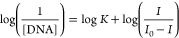
4where *I*_0_ and *I* are the peak currents of compound and compound–DNA complexes, respectively.^56^

#### 2.7.6 Time-Resolved Fluorescence

A study was carried out using TRF spectroscopy to decide the lifetime decay measurement of free compound (excited at 280 nm) and when DNA is presented. The investigations were carried out at room temperature with a quartz cuvette with an optical path of 10 mm. Using a dilute suspension of colloidal silica to scatter the excitation beam, the instrument response function may be determined. Reduced statistic and residual distributions were used to test the fit’s adequacy.^57−59^

#### 2.7.7 Antioxidant Assay

The antioxidant activity of the **3a** and **3h** was determined using the DPPH (2,2-diphenyl-2-picrylhydrazyl) radical scavenging technique, as reported.^60,73^ In methanol, the DPPH radical has a purple color and has a strong absorption band with a maximum of 517 nm. Compound concentrations ranging from 1 to 4 μM were mixed with DPPH at a concentration of 10 μM. The mixtures were properly mixed and then incubated at room temperature for 1 h in the dark. The standard reference was ascorbic acid. As a control, DPPH in methanol was utilized. The % of DPPH radical scavenged was used to calculate the antioxidant activity of the compounds, which was calculated using eq 5:
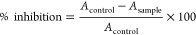
5where *A*_control_ = DPPH absorbance; *A*_sample_ = sample with DPPH absorbance.

## 3 Results and Discussion

### 3.1 Chemistry

**Figure 4:**
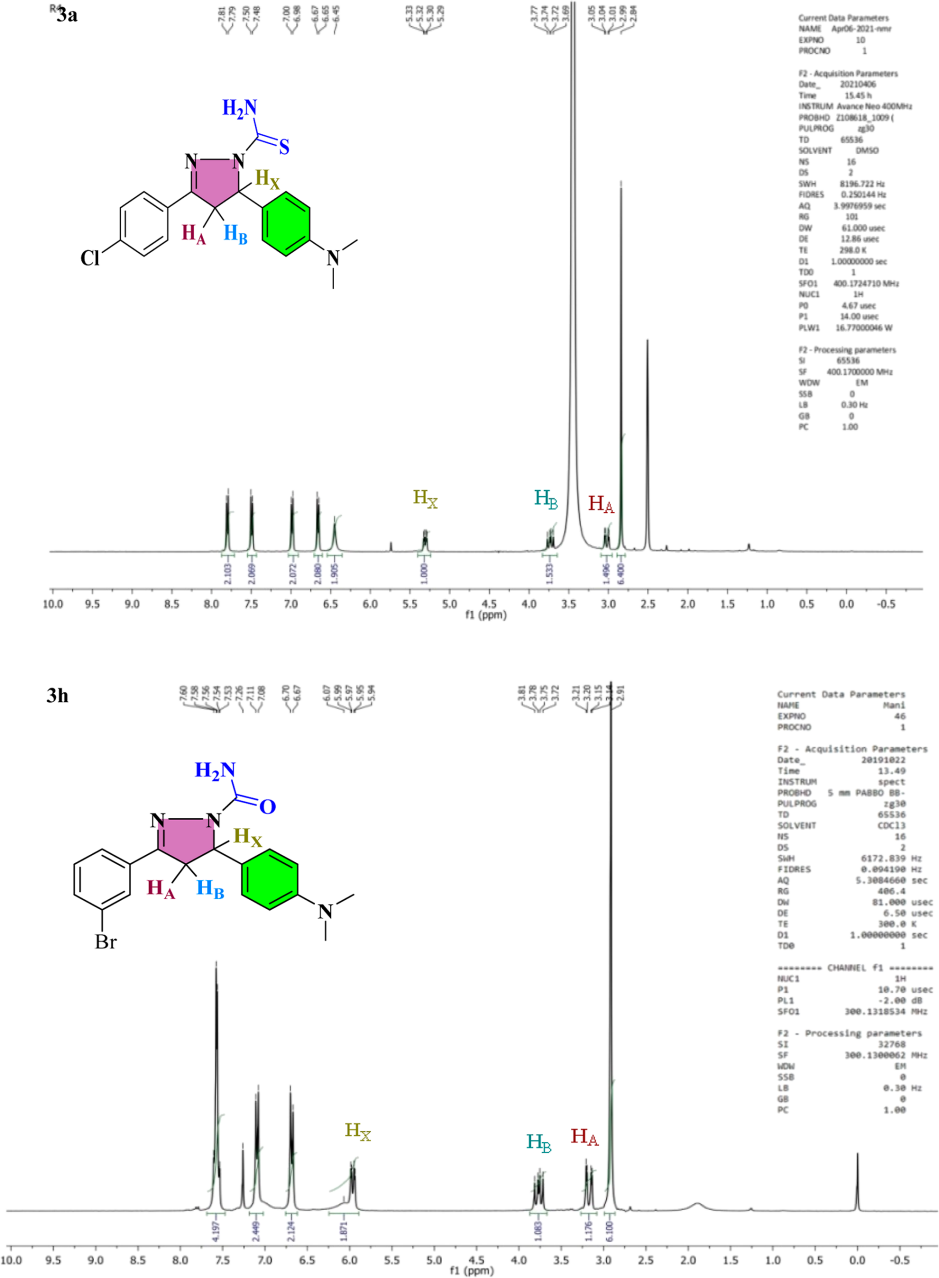
^1^H NMR spectra of compounds **3a** and **3h**.

The nucleophilic addition procedure was used to treat appropriate chalcone analogs (**2a**–**2l**) with thiosemicarbazide/semicarbazide in glacial acetic acid. The pyrazoline ring is synthesized by 1,4 nucleophilic attack of thiosemicarbazide/semicarbazide on chalcone derivatives, followed by cyclization and finally, dehydration. In IR spectra of pyrazoline analogs (**3a**–**3l**), the vibrational bands at 3122–3491, 1557–1591, and 1322–1364 cm^–1^ correspond to the NH_2_, nitrile, and C=S functional groups, respectively. The ^1^H NMR spectra of analogs show an ABX pattern among −CH_2_ protons at 3.02–3.25 ppm (H_A_) and 3.73–4.12 ppm (H_B_) as a doublet of doublet confirmed the creation of the pyrazoline ring. When a 2*J* coupling occurs with a proton from the −CH_2_ group that is not magnetically equivalent, the −CH proton exhibits a doublet of doublet and resonates at 5.31–5.97 ppm (H_X_). At 6.50–9.29 ppm, aromatic proton signals were detected. Carbon signals for −CH_2_ and methyne were detected in the range of 40.33–45.54 ppm using ^13^C NMR spectroscopy. The other signal, which ranges from 154.85 to 178.58 ppm and is attributed to −C=O/–C=S, supports the carboxamide/carbothioamide assessment. The ^1^H NMR spectra of lead compounds **3a** and **3h** are given in Figure 4 and HRMS spectra of both lead compounds are given in Figure S5, and the remaining ^1^H and ^13^C NMR, IR, mass, and HPLC spectral analyses were also supported by the structure of pyrazoline analogs (**3a**–**3l**) (Figures S1–S4).

### 3.2 Cytotoxicity

**Figure 5:**
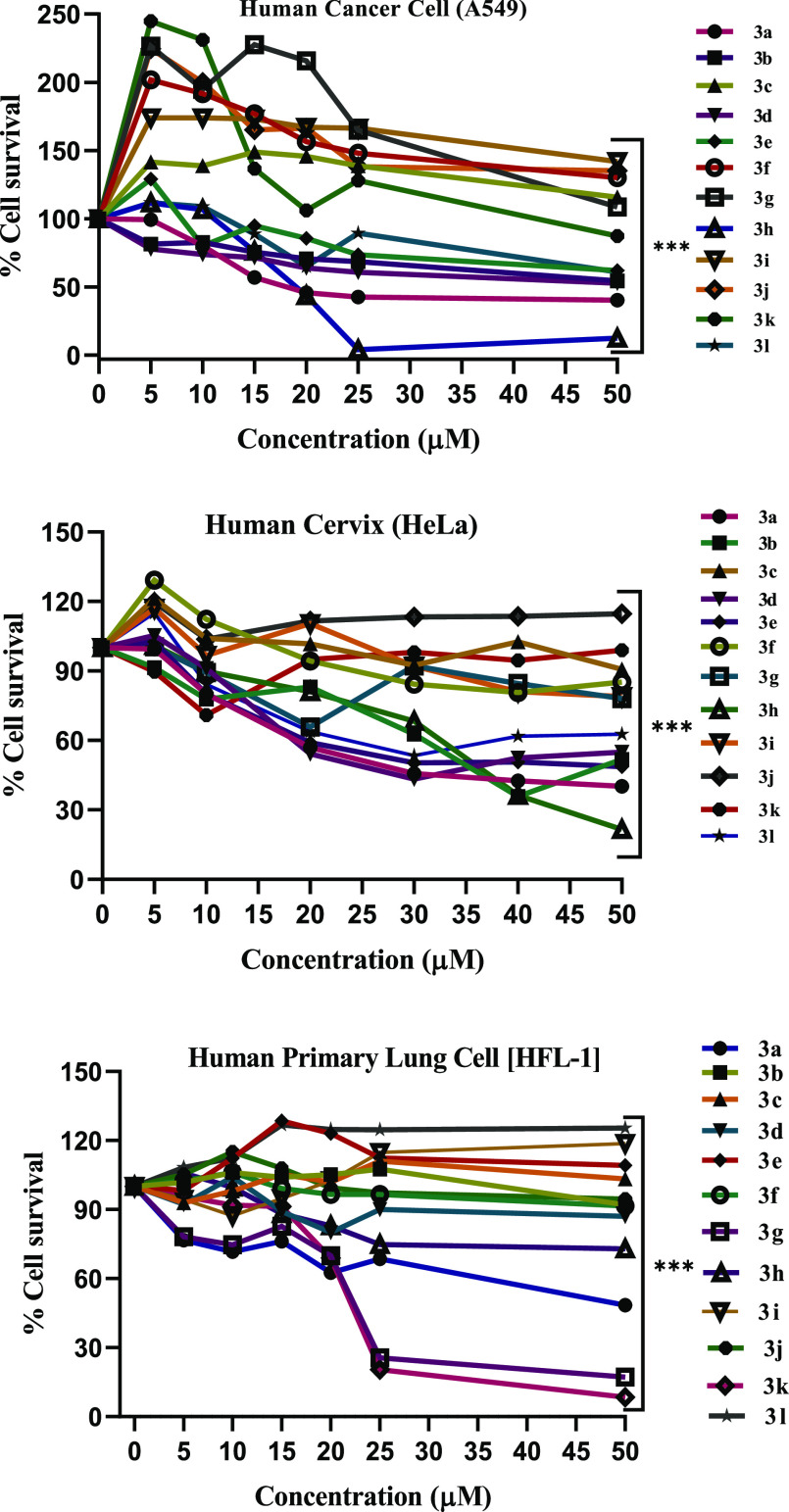
Dose–response curve of compounds **3a**–**3l** concentration vs % cell survival, and obtained results are given as mean ± SEM. *** indicates *P* less than 0.001. Non-parametric *t*-test was used for calculating statistical values by using GraphPad prism.

Cytotoxicity of pyrazoline derivatives **3a**–**3l** against human lung cancer (**A549**), cervical cancer cell line (**HeLa**), and human primary normal lung cell (**HFL-1**) were assessed in vitro using the MTT assay.^62^ Staurosporine (STS) was used as a standard drug. It is extensively used to induce apoptosis in cell culture systems, including a large number of cancer cells. Furthermore, staurosporine is well established in cell culture as a positive control to induce cell death. Considering that our study is evaluating the anticancer activity of the synthesized drug products, which act by inducing the apoptosis, we thus preferred to use staurosporine as a control drug. The results in terms of IC_50_ values are given in Table 1, and dose–response curve is also shown in Figure 5. The IC_50_ values demonstrate that mostly pyrazoline derivatives have moderate to good cytotoxicity against A549 and HeLa cell lines, and extremely low toxicity against HFL-1. Compounds **3a**, **3d**, **3e**, **3h**, **3k**, and **3l** showed remarkable cytotoxicity as compared to the reference drug (staurosporine). Analogs *p-*CH_3_ substituent **3e** showed IC_50_ values of 37.07 ± 0.14 and 14.05 ± 0.40 μM against **A549** and **HeLa** cell lines, respectively. Analog **3l** containing para *N,N*-diethyl group of one benzene ring and other benzene-containing −Br at para position exhibited IC_50_ values of 43.93 ± 0.13 and 17.65 ± 0.42 μM against **A549** and **HeLa** cell lines, respectively. Analog *ortho*-Cl substituent **3d** showed an IC_50_ value 15.0 ± 0.46 μM against the **HeLa** cell line. Analog **3k** showed an IC_50_ value of 52.76 ± 0.28 μM against human lung cancer cell (**A549**). The compounds **3a** and **3h** demonstrate magnificent cytotoxicity IC_50_ values of **13.49** ± **0.17** and **22.54** ± **0.25** μM against **A549** and **17.52** ± **0.09** and **24.14** ± **0.86** μM against the **HeLa** cancer cell lines.

**Table 1:**
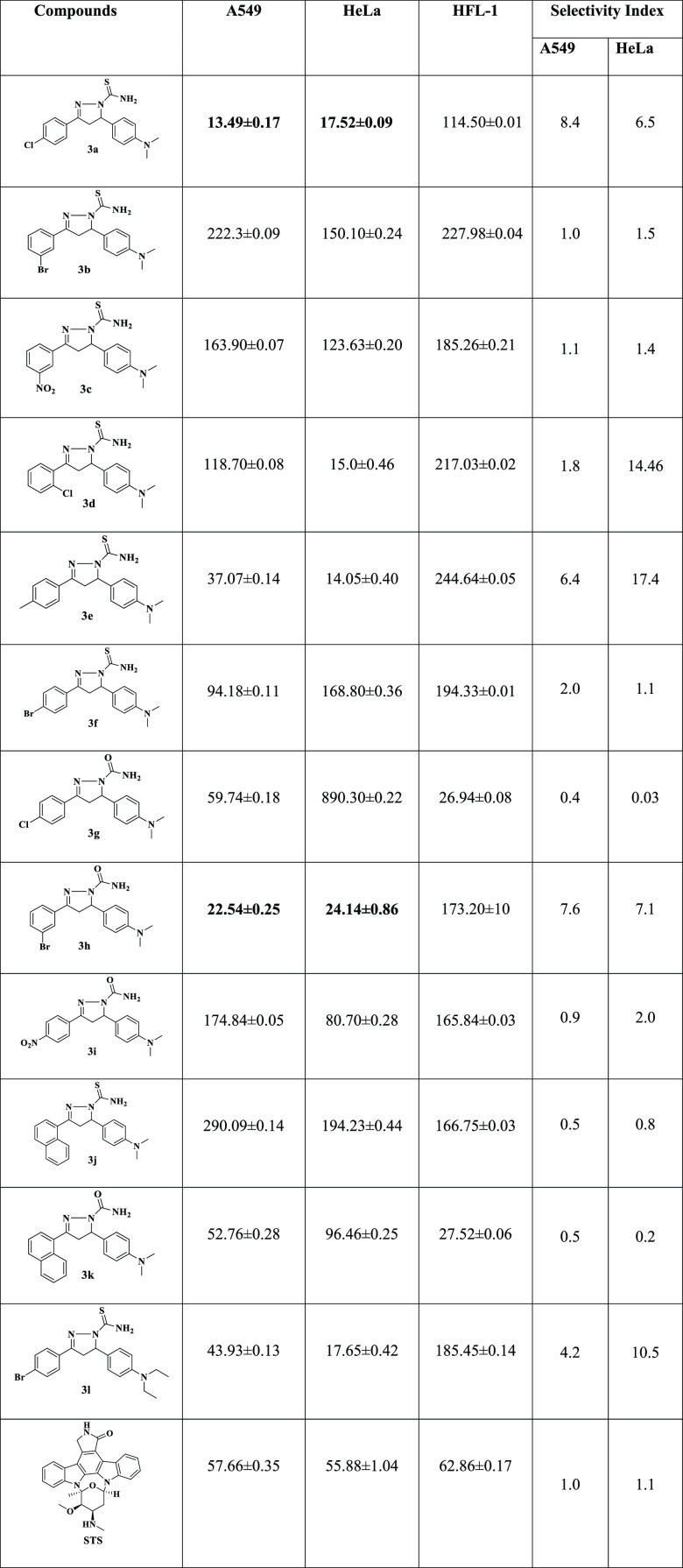
Selectivity Index (SI) Shows That Pyrazoline Derivatives,**3a** and **3h**, Were More Selective for Both Cancer Cell Lines as Compared to the Reference Drugs

### 3.3 Apoptosis

**Figure 6:**
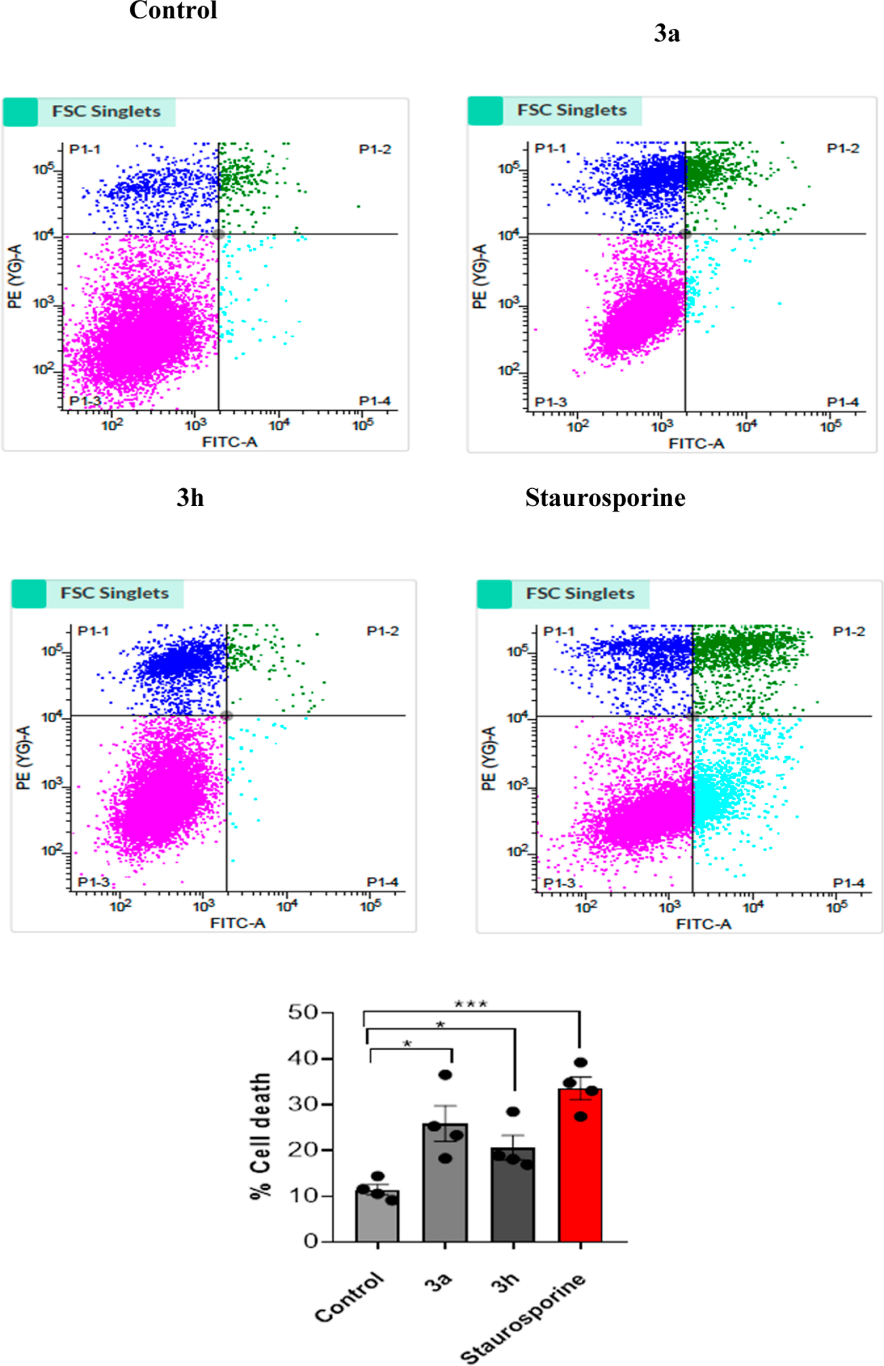
Graphical dot plots of apoptotic A549 cells following 24 h of treatment with 4 μM concentrations of compounds **3a**, **3h**, control, and reference drug. Flow cytometry was used to evaluate the cells after they were extracted and labeled with Annexin-V (AV) and PI. For the analysis of statistical significance between drug and control, the histogram investigation of drug treatment has been used. The results are shown in terms of mean ± SEM. * indicates *P* less than 0.05 and *** indicates *P* less than 0.001 with *n* = 4 for each analysis. Non-parametric *t*-test was used for calculating statistical values by using the GraphPad prism.

We employed two lead compounds **3a** and **3h** for flow cytometry analysis to validate the MTT findings of cell death and provide a quantitative evaluation of cell death.^63^ The % of cells that are positive in each of the three sub-regions was used to calculate cell death; PI-positive cells (Q1), AV + PI positive cells (Q2), AV positive cells(Q3), and live cells(Q4). In the case of staurosporine-treated cells, there was an increase in signal from Q1 to Q3, as well as a decrease in Q4. Control cells, on the other hand, saw a high % of cells in Q4, confirming the assay’s accuracy in detecting cell death. Similarly, active analogs **3a** and **3h** were analyzed with the same number of cells. An increased signal in all quadrants Q1, Q2, and Q3 was observed in cells treated with the drug, indicating enhanced cell death. Compound **3a** causes **25.86%** (12.23 + 11.69 + 1.94) cell death, while **3h** causes **20.60%** (13.48 + 6.02 + 1.08), compared to **11.41%** in the control (9.72 + 1.25 + 0.43). These findings indicate that both active analogs cause considerable cell death, as compared to staurosporine (STA)-induced cell death (Figure 6). Overall, these findings support those of the MTT assay, implying that both potential derivatives could be used as anti-cancer drugs.

### 3.4 Molecular Docking

**Figure 7:**
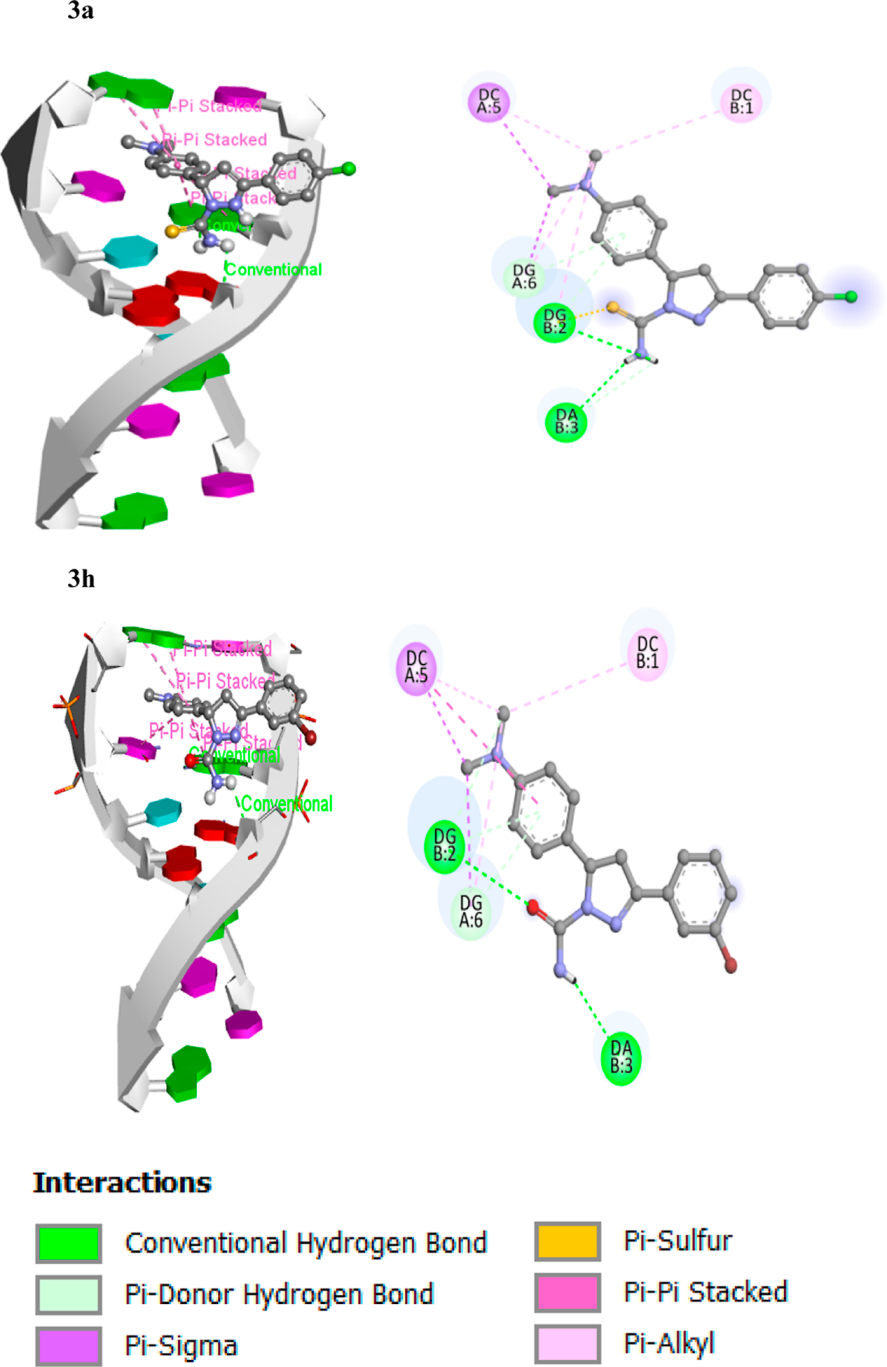
Intercalation of lead compounds **3a** and **3h** with DNA base pairs (PDB ID: 1Z3F).

The anticancer agent ellipticine binds DNA via intercalative binding based on stacking interactions along the major base-pair axis in the PDB ID: 1Z3F structure, but other binding modes have been proposed, particularly for ellipticine derivatives between base pairs and inhibition of topoisomerase II enzyme, a DNA replication enzyme, blocking it and providing potent antitumor action. Therefore, we have done molecular docking of all the synthesized derivatives with 1Z3F and also used it to confirm the experimental results and determine the binding manner of both lead compounds with DNA. All the compounds **3a**, **3b**, **3c**, **3d**, **3e**, **3f**, **3g**, **3h**, **3i**, **3j**, **3k,** and **3l** binding energies were determined to be −7.1, −6.4, −6.6, −6.3, −6.7, −6.5, −6.6, −6.9, −6.7, −7.6, −7.8, and −6.3 kcal/mol. Docked models of both compounds were prepared by using the discovery studio visualizer. A DNA duplex of sequence hexamers d(CGATCG)_2_ was used as a target for molecular docking of the pyrazoline analogs (**3a**–**3l**). Compounds **3a** and **3h** have a significant binding affinity (−7.1 and −6.9 kcal/mol, respectively), indicating that the B-DNA results were of excellent quality.^64,65^ According to the docking experiments, the analogs **3a** and **3h** interacted with DNA via an intercalative binding mode, as illustrated in Figure 7.

### 3.5 Drug Likeness

**Figure 8:**
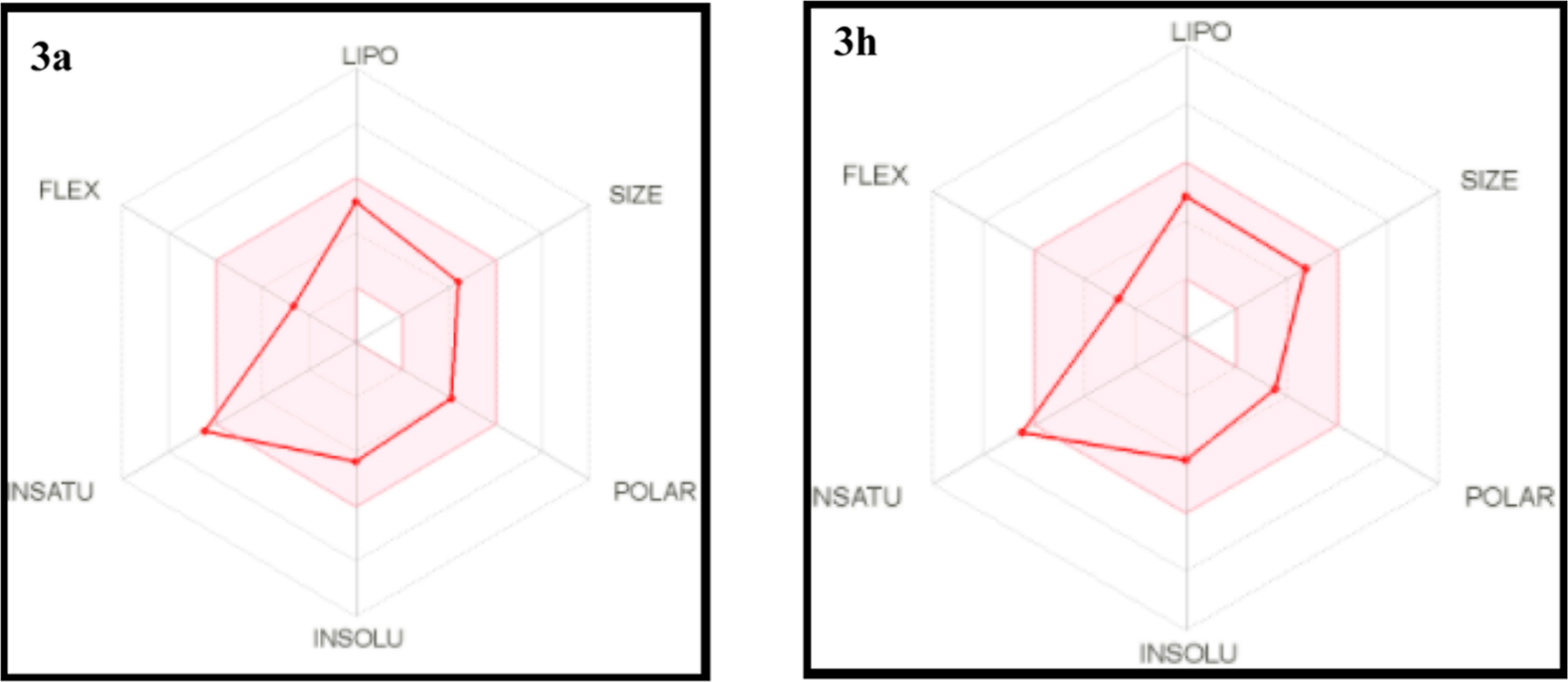
Radar charts for an indicator of oral bioavailability arising by Swiss ADME web tool; pink zone shows ideal values for oral bioavailability and red zone shows drugs.

Oral bioavailability is a critical component in the development of bioactive compounds as medicinal treatments. Lipinski’s rule of five (RO5) is a useful technique for evaluating a molecule’s drug-likeness features.^66^ The value ranges of numerous main variables for all of the compounds are given in Table 2. The molecular weight range was 338.37–431.39 g/mol, and the evaluated numbers of H-bond donors (HBD) and H-bond acceptors (HBA) to/from H_2_O molecules in aqueous solution were 0.0–2.0 and 1.0–4.0, respectively. The predicted octanol/water partition coefficients (QPlogP_o/w_) were 1.89–4.25, and the predicted aqueous solubility (QPlogS) values were −4.04 to −6.41. The predicted numbers of rotatable bonds (RB) were in the range of 4–6, and the apparent Caco-2 cell permeability (QPP_Caco_) values were 20.25–41.09 nm/s. The apparent MDCK cell permeability (QPP_MDCK_) values were 0.09–133.47 nm/s, and the predicted skin permeability (QPlogK_p_) values were −6.65 to −5.52. The human GI absorption values were high. The topological polar surface area (TPSA) values were in the range of 61.93–122.77, and the values of the logarithm of the partition coefficient (IlogP) were 0.00–3.78. The molar refractivity (MR) values were 104.54–124.24, and the fractions of sp^3^-hybridized carbon (fraction Csp^3^) were 0.18–0.26. For every compound, the number of Lipinski violations was zero. In Figure 8, the red lines which represent compounds **3a** and **3h** are incorporated in the pink area predicting good oral bioavailability. The results were established to be within an allowable extent when compared to the pyrazoline derivatives’ drug-likeness features, implying that they have the potential to be druggable compounds.

**Table 2:** Compounds (**3a**–**3l**) Drug-likeness Properties^a^

compounds	MW	RB	fraction Csp^3^	HBA	HBD	TPSA	IlogP	GI absorption	QPlogP_o/w_	QPlogK_p_	LV	MR	QPP_Caco_ (nm/s)	QPP_MDCK_ (nm/s)	QPlogS
**3a**	358.89	4	0.22	1	1	76.95	2.86	high	3.24	–6.02	0	111.74	32.72	49.43	–4.36
**3b**	402.35	4	0.26	1	1	73.71	3.27	high	4.25	–5.52	0	114.80	35.72	0.09	–5.31
**3c**	369.44	5	0.22	3	1	122.77	1.99	high	1.90	–6.65	0	115.55	20.25	1.56	–4.11
**3d**	358.89	4	0.22	1	1	76.95	2.56	high	3.18	–6.02	0	111.74	32.72	12.81	–5.36
**3e**	338.47	4	0.26	1	1	76.95	2.87	high	3.04	–6.08	0	111.70	26.75	60.12	–5.14
**3f**	403.34	4	0.22	1	1	76.95	2.94	high	3.32	–6.25	0	114.43	35.43	0.10	–5.56
**3g**	342.82	4	0.22	2	1	61.93	2.95	high	2.81	–6.35	0	104.54	23.38	133.47	–5.17
**3h**	387.27	4	0.22	2	1	61.93	3.08	high	2.90	–6.57	0	107.23	23.93	0.51	–5.37
**3i**	338.37	4	0.22	4	0	81.73	2.07	high	1.89	–6.47	0	105.68	20.84	4.61	–4.04
**3j**	374.50	4	0.18	1	1	76.95	0.00	high	3.01	–5.67	0	124.24	27.01	28.75	–6.41
**3k**	358.44	4	0.18	2	1	61.93	3.05	high	3.18	–6.00	0	117.03	22.07	66.38	–6.21
**3l**	431.39	6	0.18	1	1	76.95	3.78	high	4.04	–5.89	0	124.04	41.09	0.15	–6.34
**STA**	466.53	2	0.32	4	2	69.45	3.19	high	3.17	–6.85	0	139.39	48.76	153.09	–7.59

a Abbreviations representing physicochemical attributes for “drug likeness” analyzed from SWISS ADMET: MW = molecular weight, RB = number of rotatable bonds, HBA = number of hydrogen-bond acceptors, HBD = number of hydrogen-bond donors, TPSA = topological polar surface area, IlogP = logarithm of the partition coefficient, GI = gastrointestinal, QPlogP_o/w_ = predicted octanol/water partition coefficient, QPlogK_p_ = predicted skin permeability, LV = number of Lipinski violations, MR = molar refractivity, QPP_Caco_ = apparent Caco-2 cell permeability, QPP_MDCK_ = apparent MDCK cell permeability, QPlogS = predicted aqueous solubility.

### 3.6 DNA Binding

#### 3.6.1 Absorption Titration

**Figure 9:**
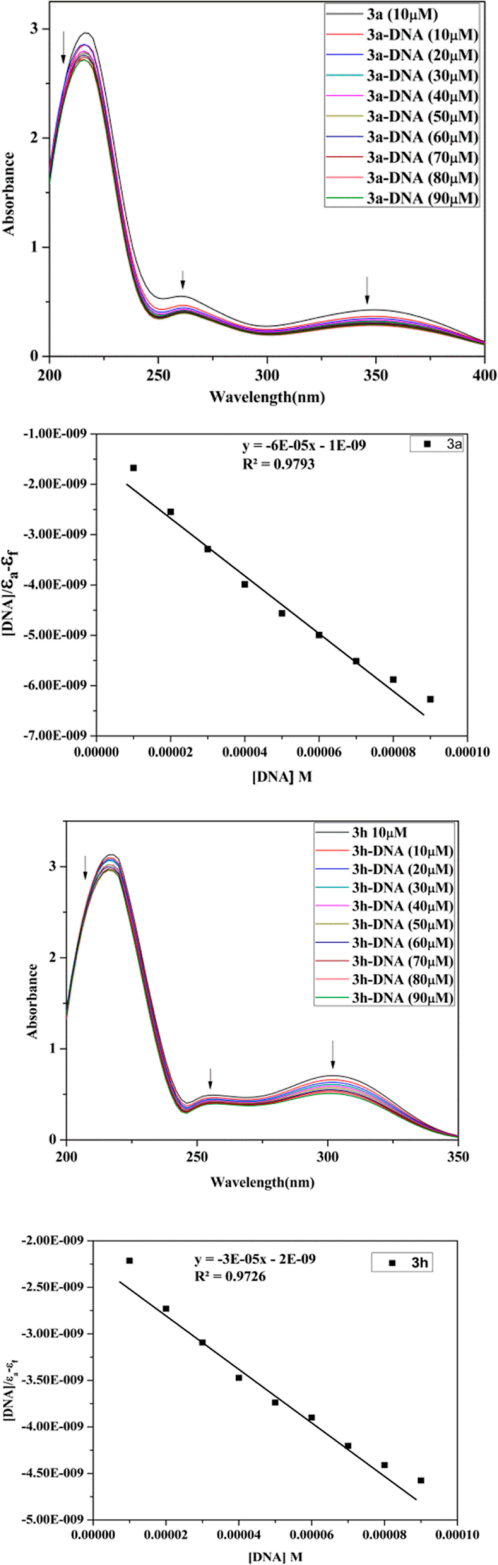
UV spectrum and inset plot between [DNA]/[ε_a_ – ε_f_] vs [DNA] of **3a** and **3h** (10 μM) with increasing amount of Ct-DNA (10–90 μM).

The capability of Ct-DNA to interact with active molecules **3a** and **3h** was investigated using UV–visible spectroscopy to investigate the binding mechanism of DNA. During compound absorption titrations, two traits emerge: hypochromic and hyperchromic. Hypochromic refers to a drop in absorbance caused by a strong stacking interaction between DNA base pairs and the aromatic chromophore of the compounds, which is thought to be a unique property of the intercalative form of binding.^67^Figure 9 illustrates absorption spectra of small molecules **3a** and **3h** without and with increasing concentration of DNA. In the absence of DNA, **3a** displayed three absorbance peaks at 220, 260, and 350 nm, and **3h** showed 215, 255, and 300 nm. With the addition of DNA, the absorbance at all three bands decreased. For quantitative investigation of the binding strength of compounds to DNA, intrinsic binding constants *K*_b_ of compounds with Ct-DNA were determined by utilizing eq 2. Intercept plots of [DNA]/(ε_a_ – ε_f_) vs [DNA] determined the intrinsic binding constants (*K*_b_) for compounds **3a** and **3h** to be 3.8 × 10^4^ and 1.3 × 10^4^ M^–1^, respectively.

#### 3.6.2 Emission Titration

**Figure 10:**
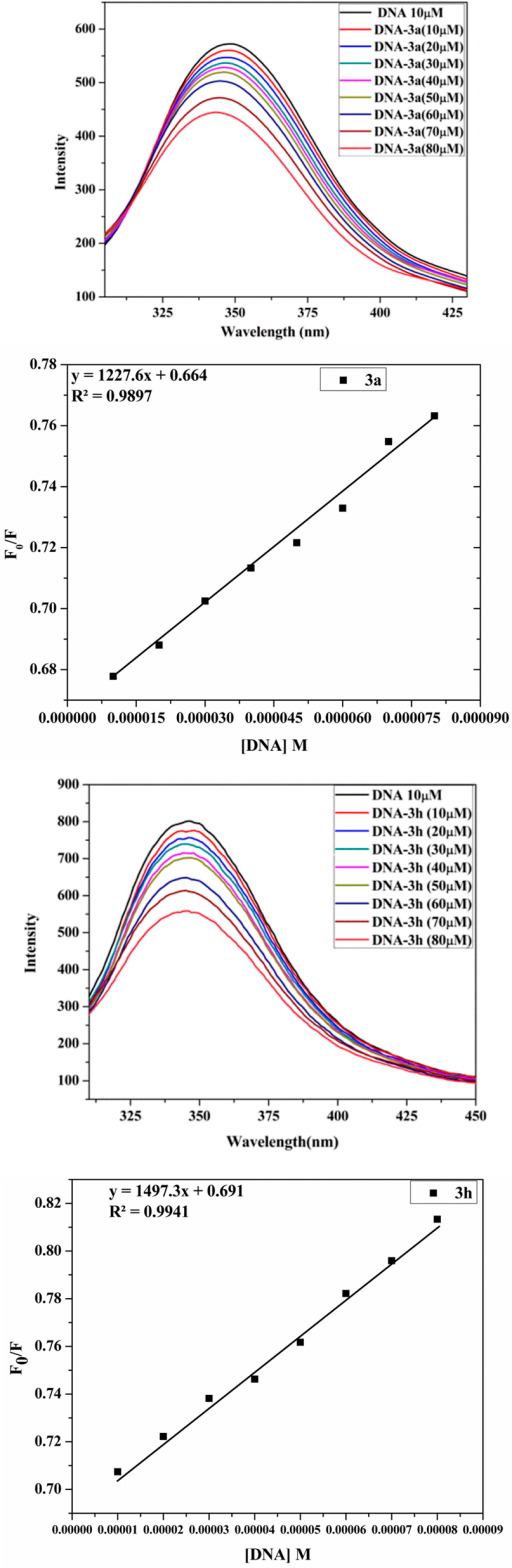
Emission titration spectra of a constant concentration of DNA (10 μM) with varying compounds **3a** and **3h** concentration (10–80 μM). The Stern–Volmer plot of compounds **3a** and **3h**.

Emission titrations were used to explore the binding properties of compounds **3a** and **3h** with DNA as a suitable addition to the previous experiments. Ct-DNA emission titrations were performed in the increasing concentration of both the compounds. Emission intensity decreases dramatically, with the addition of compounds aliquot, as seen in Figure 10. The quenching of emission of the compounds is caused by the transfer of a photoelectron from DNA’s guanine nitrogenous base to excited states of compounds.^68^ The fraction of quenching may be calculated experimentally using the value of *K*_SV_ obtained from the SV plot shown in Figure 10, and the Stern–Volmer constant (*K*_SV_) is the slope of *F*_0_/*F* versus [Q] found to be 5.80 × 10^3^ and 4.28 × 10^3^ M^–1^ for the compounds **3a** and **3h**, respectively.

#### 3.6.3 Dye Displacement Assay

**Figure 11:**
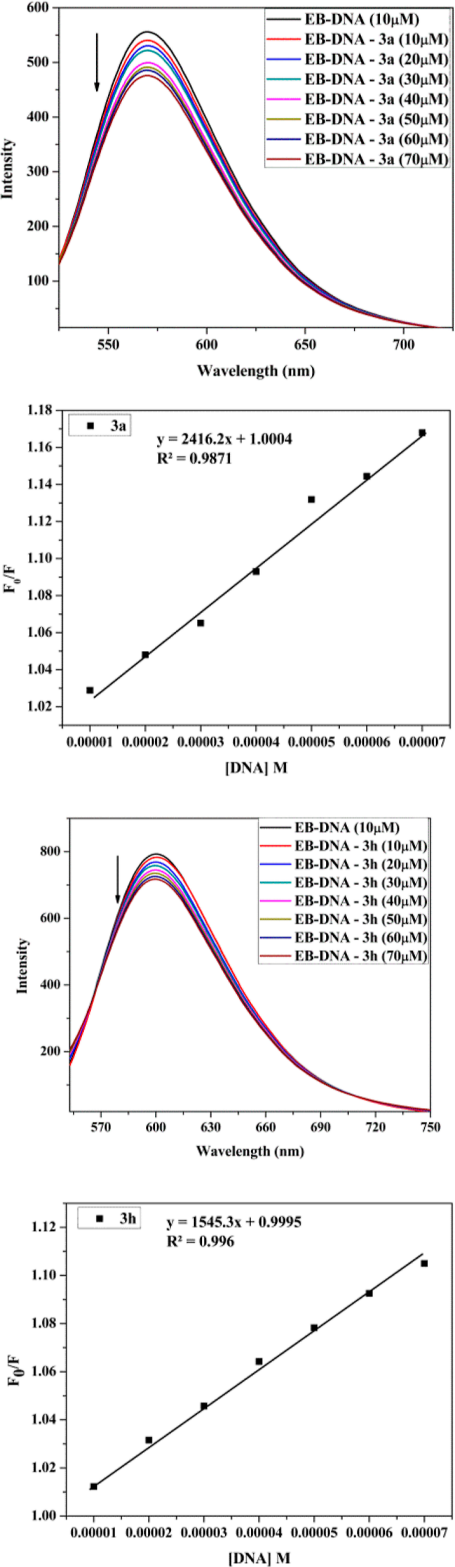
Displacement of ethidium bromide from Ct-DNA by **3a** and **3h**.

The low fluorescence intensity of EtBr offers information regarding DNA-free EtBr, which is utilized to determine the mechanism of DNA binding interaction. When ethidium bromide (EtBr) is intercalated into DNA, the emission peak is significantly increased. Fluorescence quenching was seen after the test chemical was continuously added to the EB-DNA complex. The dye displacement assay shows that compound **3c** interacts with DNA in an intercalative manner. As the concentration of both test compounds increases, the Stern–Volmer eq 3 was employed to quantify the degree of fluorescence quenching of the EB-DNA system. The value of *K*_SV_ obtained from the SV plot may be used to experimentally calculate the fraction of quenching and so predict the optimal DNA binding interaction mode. A competitive fluorescence displacement test is commonly used in the general approach to DNA binding research.^69^ The quenching suggests that compounds **3a** and **3h** cause the release of ethidium bromide from DNA helix, implying that they bind to DNA in an intercalative manner. *K*_SV_ values for the fluorescence quenching depth of EB were found to apply the Stern–Volmer plot. Stern–Volmer constant (*K*_SV_) is the slope of *F*_o_/*F* vs [Q] found to be 1.84 × 10^4^ and 1.69 × 10^4^ M^–1^ for the compounds **3a** and **3h**, respectively. The other parameters of the dye displacement measurement are mentioned in Figure 11.

#### 3.6.4 Circular Dichroism

**Figure 12:**
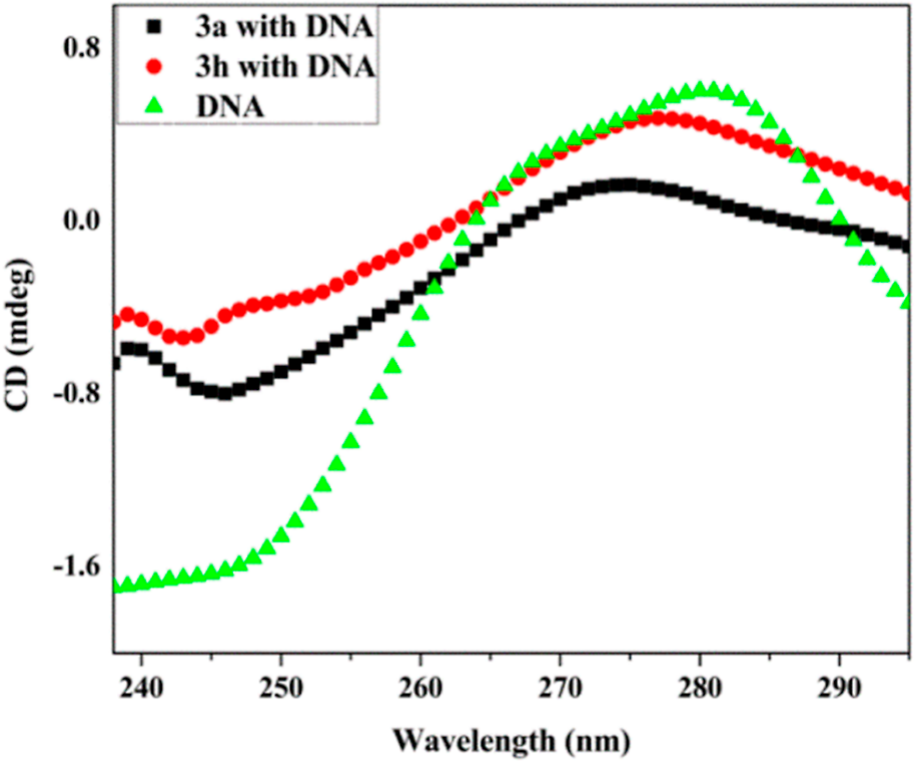
CD spectra of Ct-DNA (50 μM) without and with active analogs **3a** and **3h** (50 μM).

The circular dichroism technique is widely employed to detect conformational changes in Ct-DNA and proteins, as a result of their interactions with ligand molecules. To explore the structural modification found throughout compound–DNA binding interaction, a CD spectrum of Ct-DNA was used without and with active compounds (**3a**, **3h**). Generally, native Ct-DNA shows two distinct peaks, a positive peak at 275 nm due to DNA helicity and a negative peak at 245 nm due to π–π base stacking.^70^Figure 12 shows that compounds binding to DNA have only a significant result on a negative peak location of 245 nm and also a slight shift at 275 nm. The results demonstrate that the compounds **3a** and **3h** interact with Ct-DNA by intercalative mode, which in turn causes conformational alterations in the DNA structure.

#### 3.6.5 Cyclic Voltammetry

**Figure 13:**
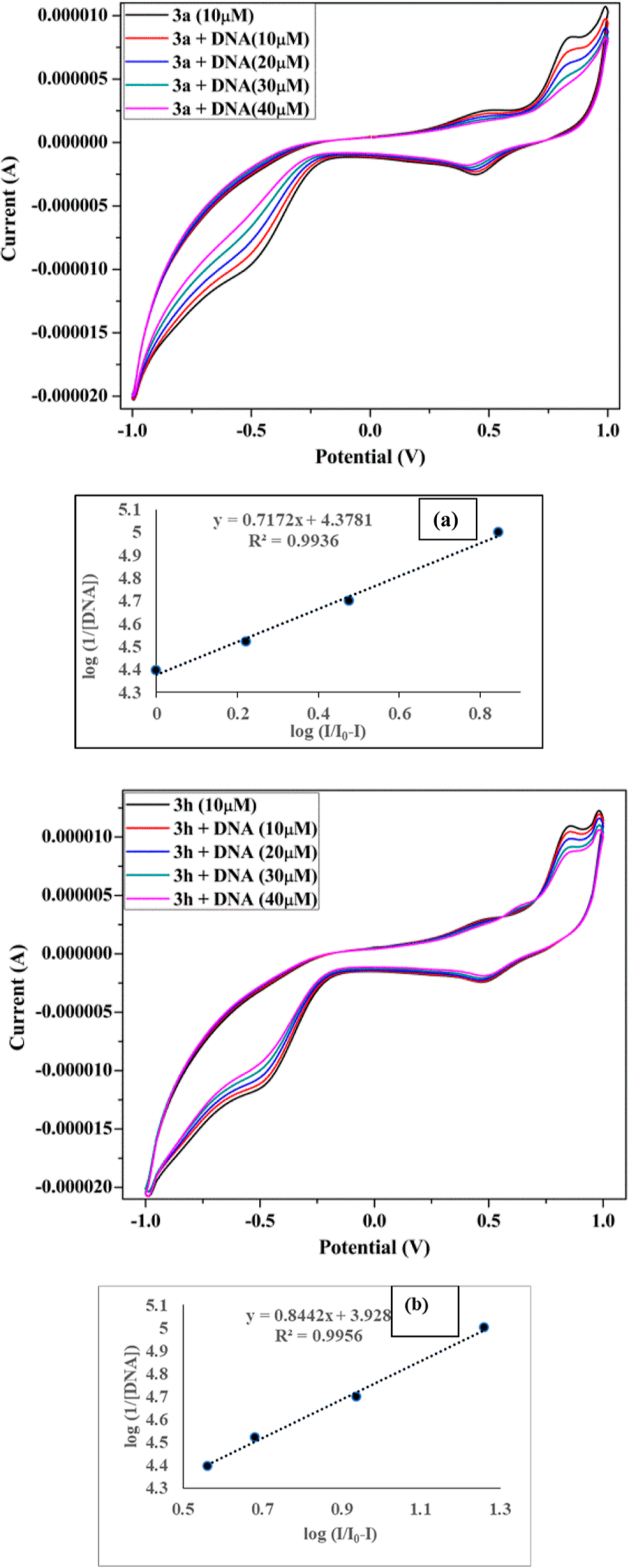
Cyclic voltammogram of 1.0 × 10^–5^ M of analogs **3a** and **3h** in Tris-buffer, at 100 mV s^–1^ scan rate free compound (black), in the presence of DNA (10–40 μM). The plot between log(*I*/*I*_0_ – I) vs log(1/[DNA]) for finding binding constant value (a,b) for **3a** and **3h,** respectively.

The manners of compound–DNA binding interaction were investigated using an electrochemical method. The change in peak potential of the cyclic voltammogram provides important information about the compound’s binding mode with DNA. Figure 13 shows a cyclic voltammogram of compounds **3a** and **3h** in the absence and presence of DNA. The peak current was reduced when Ct-DNA was added to the compounds, as shown in the graph. As a result, the development of a compound–DNA complex formation may occur. This may be due to the complex creation of compounds with bulky Ct-DNA that diffuses gradually toward the working electrode. The greater oxidation peak currents of pyrazoline analogs (**3a**, **3h**) than their DNA adduct correspond to easier mobilities and faster responses toward working electrodes due to faster diffusion. The plot between log(*I*/*I*_0_ – *I*) versus log(1/[DNA]) was utilized to calculate binding constant values *K* for compounds **3a** and **3h,** which are found to be 1.25 × 10^4^ and 7.1 × 10^3^, respectively, as depicted in ref (71).

#### 3.6.6 Time-Resolved Fluorescence

**Figure 14:**
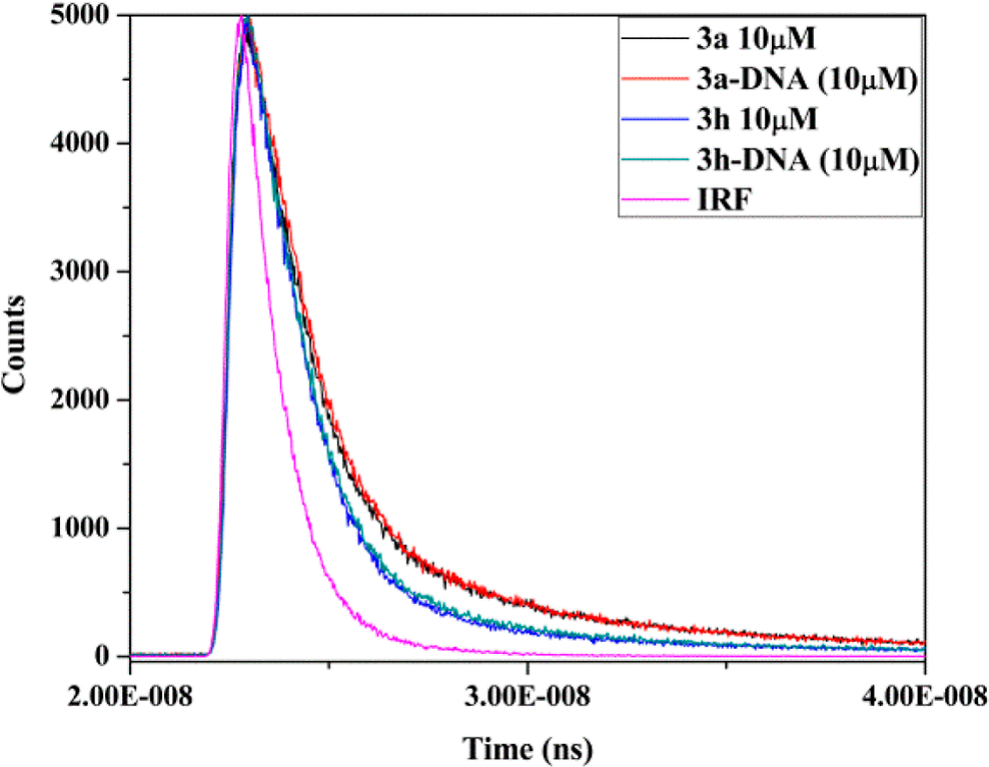
Time-resolved fluorescence graph of active compounds **3a** and **3h** without and with Ct-DNA and standard IRF.

Emission decay of lead compounds **3a** and **3h** with and without DNA is shown in Figure 14 and determined parameters are shown in Table 3; decay curve can be best fitted with a tri-exponential decay model. It was observed that the relative amplitude of the lifetime of active analogs moderately increases with the addition of DNA. Without Ct-DNA, compounds **3a** and **3h** show lifetimes τ_1_, τ_2_, and τ_3_ of 0.90, 6.60, 0.12 and 1.26, 7.46, 0.18 ns, respectively, but in the presence of DNA small changes in all lifetimes occur 1.0, 6.73, 0.21 and 1.38, 7.23, 0.29, respectively. In the case of **3a** and **3h,** we found a slight change in all amplitude τ_1_ time components from 27.81–28.57, 36.5–35.34%, τ_2_ time amplitude from 33.73–32.78, 17.41–19.64%, and τ_3_ time amplitude from 38.46–38.65, 46.09–45.05%, respectively, for upon addition of DNA. The plot of compounds **3a** and **3h** in the absence and presence of DNA demonstrate significant changes in an average lifetime because viscosity difference of compounds solution in the presence of DNA occurs. Results suggest that compound **3a** has more binding affinity as compared to compound **3h**.^72^

**Table 3:** Lifetime Fluorescence Spectral Details Feature Compounds **3a** and **3h**

Analogs	*a*_1_	*a*_2_	*a*_3_	τ_1_	τ_2_	τ_3_	⟨*t*⟩	χ^2^
**3a**	0.10	0.02	0.88	1.26	7.46	0.18	0.43	1.08
**3a**–DNA	0.13	0.03	0.84	1.38	7.23	0.29	0.64	1.13
**3h**	0.1	0.01	0.89	0.90	6.60	0.12	0.25	1.13
**3h**–DNA	0.14	0.01	0.85	1.0	6.73	0.21	0.39	1.04

The data were fitted using triexponential function equation given below:
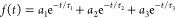


The average lifetime was determined by applying the equation given below:
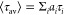


#### 3.6.7 Antioxidant Assay

**Figure 15:**
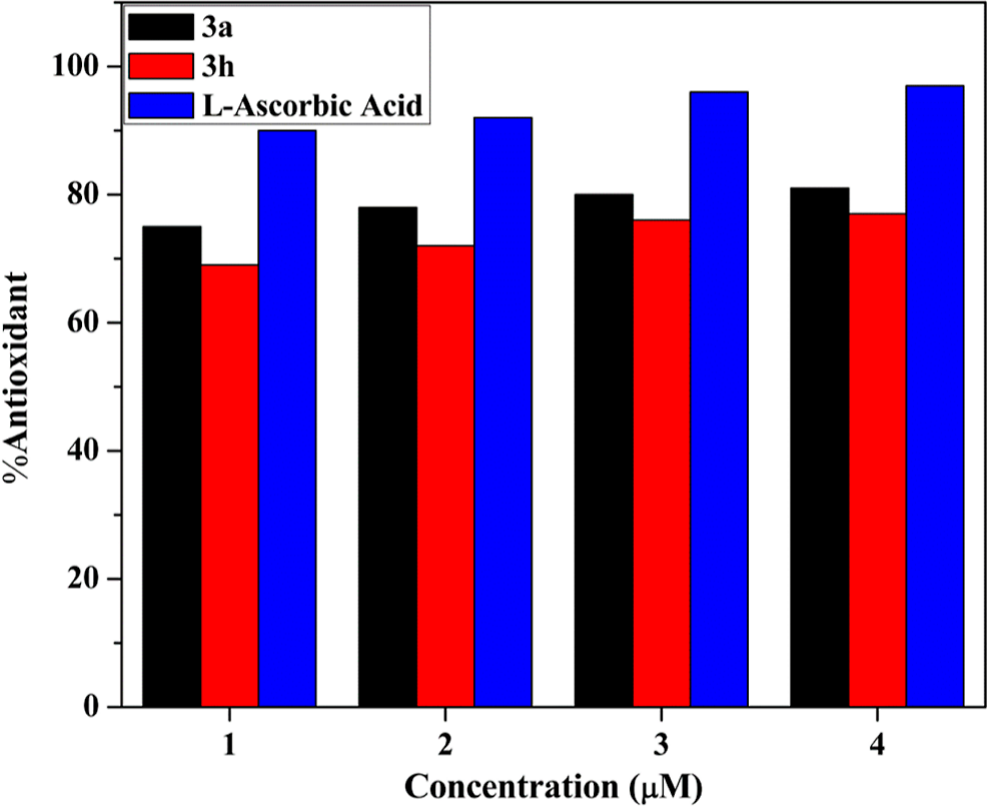
Antioxidant bar graph of active analogs **3a** and **3h**.

The free radical reduced which is present on DPPH accepts an electron or an H-atom from a molecule. The color change from violet to yellow was detected after 1 h of incubation. The UV–visible spectrophotometer absorbance decreases (516 nm wavelength) after the addition of compounds **3a** and **3h** due to pairing of its lone-pair electron with electron of an antioxidant to form reduced DPPH. The compounds **3a** and **3h** show a strong antioxidant activity with IC_50_ values of 0.132 ± 0.012 and 0.215 ± 0.025 μg/mL.^73^ A lower IC_50_ value implies higher antioxidant activity. Higher antioxidant activity of **3a** as compared to **3h** might be due to the presence of nitrogen, chlorine atom, and carbothioamide group in their structure, which donate the electron density easily. The antioxidant activity of both the compounds was significant, as illustrated in Figure 15.

## 4 Conclusions

We demonstrated the synthesis of a series of carbothioamide/carboxamide-based pyrazoline derivatives and characterized these by using different spectroscopic methods. All the analogs have shown moderate to excellent cytotoxicity against A549 and HeLa cancer cell lines. The lead compounds, **3a** and **3b** exhibited significant inhibitory activity toward the A549 than the HeLa cancer cell lines. Anticancer activity of target compounds also has been validated by apoptosis. The binding affinity of both the active analogue with DNA was carried out by using UV–visible, fluorescence, competitive assay method using ethidium bromide, circular dichroism, cyclic voltammetry, and time-resolved fluorescence. The molecular docking analysis of derivatives **3a** and **3h** with the DNA hexamer shows the noncovalent intercalation binding interactions which also verify the experimental results. The drug-likeness property of all derivatives was determined via ADMET assay. Thus, the anticancer mechanism of action of these compounds could be attributed to the DNA intercalation binding manner.

## Supporting Information Available

^1^H, ^13^C NMR, FT-IR spectra, mass analysis, HPLC, and HRMS spectra of the synthesized compounds (PDF)

## References

[ref1] Tan Y.; Chen H.; Zhang J.; Cai L.; Jin S.; Song D.; Yang T.; Guo Z.; Wang X. Platinum(IV) complexes as inhibitors of CD47-SIRPα axis for chemoimmunotherapy of cancer. Eur. J. Med. Chem. 2022, 229, 114047 10.1016/j.ejmech.2021.114047. 34915428

[ref2] Han H.; He C.; Chen X.; Luo Y.; Yang M.; Wen Z.; Hu J.; Lin F.; Han M.; Yin T.; Yang R.; Lin H.; Qi J.; Yang Y. Shikonin *N*-benzyl matrinic acid ester derivatives as novel telomerase inhibitors with potent activity against lung cancer cell lines. Bioorg. Med. Chem. Lett. 2022, 57, 128503 10.1016/j.bmcl.2021.128503. 34922028

[ref3] Zhang Y.; Yang R.; Yin H.-M.; Zhou B.; Hong M.; Zhu B.; Qi M.-H.; Ren G.-B. Cocrystals of flavonoids with 4,4′-ethylenebispyridine: Crystal structures analysis, dissolution behavior, and anti-tumor activity. J. Mol. Struct. 2022, 1252, 132150 10.1016/j.molstruc.2021.132150.

[ref4] Meng Q.; Zhong S.; Xu L.; Wang J.; Zhang Z.; Gao Y.; Cui X. Review on design strategies and considerations of polysaccharide-based smart drug delivery systems for cancer therapy. Carbohydr. Polym. 2022, 279, 119013 10.1016/j.carbpol.2021.119013. 34980356

[ref5] Chattopadhyay S. Simplified Treatment of Electronic Structures of the Lowest Singlet and Triplet States of Didehydropyrazines. J. Phys. Chem. A 2019, 123, 5980–5994. 10.1021/acs.jpca.9b03998. 31287697

[ref6] Stefanes N. M.; Toigo J.; Maioral M. F.; Jacques A. V.; Chiaradia-Delatorre L. D.; Perondi D. M.; Ribeiro A. A. B.; Bigolin Á.; Pirath I. M. S.; Duarte B. F.; Nunes R. J.; Santos-Silva M. C. Synthesis of novel pyrazoline derivatives and the evaluation of death mechanisms involved in their antileukemic activity. Bioorg. Med. Chem. 2019, 27, 375–382. 10.1016/j.bmc.2018.12.012. 30579801

[ref7] Nadaraj V.; Selvi S. T.; Mohan S.; Thangadurai T. D. Microwave-assisted synthesis and pharmacological studies of novel 5-deazaalloxazine derivatives. Med. Chem. Res. 2012, 21, 2911–2919. 10.1007/s00044-011-9811-1.

[ref8] Chiririwa H.; Moss J. R.; Hendricks D.; Smith G. S.; Meijboom R. Synthesis Characterisation and in Vitro Evaluation of Platinum(II) and Gold(I) Iminophosphine Complexes for Anticancer Activity. Polyhedron 2013, 49, 29–35. 10.1016/j.poly.2012.09.053.

[ref9] Mitra I.; Reddy B V. P.; Mukherjee S.; Moi S. C. Kinetic and Mechanistic Study of Substitution on a Cytotoxic Pt II Complex with Biologically Relevant Thiols and a Density Functional Study. Polyhedron 2017, 128, 46–56. 10.1016/j.poly.2017.02.019.

[ref10] Chate A. V.; Redlawar A. A.; Bondle G. M.; Sarkate A. P.; Tiwari S. V.; Lokwani D. K. A new efficient domino approach for the synthesis of coumarin-pyrazolines as antimicrobial agents targeting bacterial alanine- ligase. New J. Chem. 2019, 43, 9002–9011. 10.1039/c9nj00703b.

[ref11] Liu Y.; Fu L.; Wu J.; Liu M.; Wang G.; Liu B.; Zhang L. Transcriptional cyclin-dependent kinases: Potential drug targets in cancer therapy. Eur. J. Med. Chem. 2022, 229, 114056 10.1016/j.ejmech.2021.114056. 34942431

[ref12] Hassan R. A.; Emam S. H.; Hwang D.; Kim G.-D.; Hassanin S. O.; Khalil M. G.; Abdou A. M.; Sonousi A. Design, synthesis and evaluation of anticancer activity of new pyrazoline derivatives by down-regulation of VEGF: Molecular docking and apoptosis inducing activity. Bioorg. Chem. 2022, 118, 105487 10.1016/j.bioorg.2021.105487. 34798455

[ref13] Rasal N. K.; Sonawane R. B.; Jagtap S. V. Synthesis, Characterization, and Biological Study of 3-Trifluoromethylpyrazole Tethered Chalcone-Pyrrole and Pyrazoline-Pyrrole Derivatives. Chem. Biodiversity 2021, 18, e2100504 10.1002/cbdv.202100504. 34409724

[ref14] Alam R.; Wahi D.; Singh R.; Sinha D.; Tandon V.; Grover A.; Rahisuddin Design, synthesis, cytotoxicity, HuTopoIIα inhibitory activity and molecular docking studies of pyrazole derivatives as potential anticancer agents. Bioorg. Chem. 2016, 69, 77–90. 10.1016/j.bioorg.2016.10.001. 27744115

[ref15] Yavari M. A.; Adiloglu Y.; Saglamtas R.; Tutar A.; Gulcin I.; Menzek A. Synthesis and some enzyme inhibition effects of isoxazoline and pyrazoline derivatives including benzonorbornene unit. J. Biochem. Mol. Toxicol. 2022, 36, 22952 10.1002/jbt.22952. 34783117

[ref16] Benupani Sahu B.; Rajapandi R.; Avik Maji A.; Abhik Paul A.; Tanushree Singha T.; Tapan Kumar Maity T. K. Synthesis, characterization, molecular docking and in vitro anticancer activity of 3-(4-methoxyphenyl)-5-substituted phenyl-2-pyrazoline-1-carbothioamide. Int. J. Res. Pharm. Sci. 2021, 12, 1648–1658. 10.26452/ijrps.v12i2.4759.

[ref17] Hayat F.; Salahuddin A.; Umar S.; Azam A. Synthesis, characterization, antiamoebic activity and cytotoxicity of novel series of pyrazoline derivatives bearing quinoline tail. Eur. J. Med. Chem. 2010, 45, 4669–4675. 10.1016/j.ejmech.2010.07.028. 20696501

[ref18] Li Q.-S.; Shen B.-N.; Zhang Z.; Luo S.; Ruan B.-F. Discovery of Anticancer Agents from 2-Pyrazoline-Based Compounds. Curr. Med. Chem. 2021, 28, 940–962. 10.2174/0929867327666200306120151. 32141413

[ref19] Matiadis D.; Sagnou M. Pyrazoline Hybrids as Promising Anticancer Agents: An Up-to-Date Overview. Int. J. Mol. Sci. 2020, 21, 5507 10.3390/ijms21155507. PMC7432644 32752126

[ref20] Karabacak M.; Altıntop M.; İbrahim Çiftçi H.; Koga R.; Otsuka M.; Fujita M.; Özdemir A. Synthesis and Evaluation of New Pyrazoline Derivatives as Potential Anticancer Agents. Molecules 2015, 20, 19066–19084. 10.3390/molecules201019066. 26492233 PMC6332424

[ref21] Sever B.; Altıntop M. D.; Radwan M. O.; Özdemir A.; Otsuka M.; Fujita M.; Ciftci H. I. Design, Synthesis and Biological Evaluation of a New Series of Thiazolyl-Pyrazolines as Dual EGFR and HER2 Inhibitors. Eur. J. Med. Chem. 2019, 182, 111648 10.1016/j.ejmech.2019.111648. 31493743

[ref22] Karad S. C.; Purohit V. B.; Raval D. K. Design, synthesis, and characterization of fluoro substituted novel pyrazolylpyrazolines scaffold and their pharmacological screening. Eur. J. Med. Chem. 2014, 84, 51–58. 10.1016/j.ejmech.2014.07.008. 25016227

[ref23] Altıntop M. D.; Özdemir A.; Turan-Zitouni G.; Ilgın S.; Atlı Ö.; Demirel R.; Kaplancıklı Z. A. A novel series of thiazolyl–pyrazoline derivatives: Synthesis and evaluation of antifungal activity, cytotoxicity, and genotoxicity. Eur. J. Med. Chem. 2015, 92, 342–352. 10.1016/j.ejmech.2014.12.055. 25576739

[ref24] Havrylyuk D.; Zimenkovsky B.; Vasylenko O.; Day C. W.; Smee D. F.; Grellier P.; Lesyk R. Synthesis and biological activity evaluation of 5-pyrazoline substituted 4-thiazolidinones. Eur. J. Med. Chem. 2013, 66, 228–237. 10.1016/j.ejmech.2013.05.044. 23811085 PMC7115615

[ref25] Nehra B.; Rulhania S.; Jaswal S.; Kumar B.; Singh G.; Monga V. Recent advancements in the development of bioactive pyrazoline derivatives. Eur. J. Med. Chem. 2020, 205, 112666 10.1016/j.ejmech.2020.112666. 32795767

[ref26] Arwansyah A.; Arif A. R.; Syahputra G.; Sukarti S.; Kurniawan I. Theoretical studies of Thiazolyl-Pyrazoline derivatives as promising drugs against malaria by QSAR modelling combined with molecular docking and molecular dynamics simulation. Mol. Simul. 2021, 47, 988–1001. 10.1080/08927022.2021.1935926.

[ref27] Abdel-Halim M.; Tinsley H.; Keeton A. B.; Weam M.; Atta N. H.; Hammam M. A.; Hefnawy A.; Hartmann R. W.; Engel M.; Piazza G. A.; Abadi A. H. Discovery of trisubstituted pyrazolines as a novel scaffold for the development of selective phosphodiesterase 5 inhibitors. Bioorg. Chem. 2020, 104, 104322 10.1016/j.bioorg.2020.104322. 33142429 PMC7686114

[ref28] Zhang Z.; Cao P.; Fang M.; Zou T.; Han J.; Duan Y.; Xu H.; Yang X.; Li Q.-S. Design, synthesis, and SAR study of novel 4,5-dihydropyrazole-Thiazole derivatives with anti-inflammatory activities for the treatment of sepsis. Eur. J. Med. Chem. 2021, 225, 113743 10.1016/j.ejmech.2021.113743. 34403978

[ref29] Marella A.; Rahmat Ali M.; Tauquir Alam M.; Saha R.; Tanwar O.; Akhter M.; Shaquiquzzaman M.; Mumtaz Alam M. Pyrazolines: A Biological Review, Mini-Reviews. Med. Chem. 2013, 13, 921–931. 10.2174/1389557511313060012. 23544604

[ref30] Joshi R. S.; Mandhane P. G.; Diwakar S. D.; Dabhade S. K.; Gill C. H. Synthesis, analgesic and anti-inflammatory activities of some novel pyrazolines derivatives. Bioorg. Med. Chem. Lett. 2010, 20, 3721–3725. 10.1016/j.bmcl.2010.04.082. 20529688

[ref31] Abdel-Sayed M. A.; Bayomi S. M.; El-Sherbeny M. A.; Abdel-Aziz N. I.; ElTahir K. E. H.; Shehatou G. S. G.; Abdel-Aziz A. A.-M. Synthesis, anti-inflammatory, analgesic, COX-1/2 inhibition activities and molecular docking study of pyrazoline derivatives. Bioorg. Med. Chem. 2016, 24, 2032–2042. 10.1016/j.bmc.2016.03.032. 27025563

[ref32] Rezki N.; Al-blewi F. F.; Al-Sodies S. A.; Alnuzha A. K.; Messali M.; Ali I.; Aouad M. R. Synthesis, Characterization, DNA Binding, Anticancer, and Molecular Docking Studies of Novel Imidazolium-Based Ionic Liquids with Fluorinated Phenylacetamide Tethers. ACS Omega 2020, 5, 4807–4815. 10.1021/acsomega.9b03468. 32201766 PMC7081306

[ref33] Scoditti S.; Dabbish E.; Russo N.; Mazzone G.; Sicilia E. Anticancer Activity, DNA Binding, and Photodynamic Properties of an N∧C∧N-Coordinated Pt(II) Complex. Inorg. Chem. 2021, 60, 10350–10360. 10.1021/acs.inorgchem.1c00822. 34170132

[ref34] Al-Rashood S. T.; Elshahawy S. S.; El-Qaias A. M.; El-Behedy D. S.; Hassanin A. A.; El-Sayed S. M.; El-Messery S. M.; Shaldam M. A.; Hassan G. S. New thiazolopyrimidine as anticancer agents: Synthesis, biological evaluation, DNA binding, molecular modeling and ADMET study. Bioorg. Med. Chem. Lett. 2020, 30, 127611 10.1016/j.bmcl.2020.127611. 33068712

[ref35] Nekvinda J.; Różycka D.; Rykowski S.; Wyszko E.; Fedoruk-Wyszomirska A.; Gurda D.; Orlicka-Płocka M.; Giel-Pietraszuk M.; Kiliszek A.; Rypniewski W.; Bachorz R.; Wojcieszak J.; Grüner B.; Olejniczak A. B. Synthesis of naphthalimide-carborane and metallacarborane conjugates: Anticancer activity, DNA binding ability. Bioorg. Chem. 2020, 94, 103432 10.1016/j.bioorg.2019.103432. 31776032

[ref36] Lv P.-C.; Li H.-Q.; Sun J.; Zhou Y.; Zhu H.-L. Synthesis and biological evaluation of pyrazole derivatives containing thiourea skeleton as anticancer agents. Bioorg. Med. Chem. 2010, 18, 4606–4614. 10.1016/j.bmc.2010.05.034. 20627597

[ref37] Zhang Y.-L.; Qin Y.-J.; Tang D.-J.; Yang M.-R.; Li B.-Y.; Wang Y.-T.; Cai H.-Y.; Wang B.-Z.; Zhu H.-L. Synthesis and Biological Evaluation of 1-Methyl-1 H -indole-Pyrazoline Hybrids as Potential Tubulin Polymerization Inhibitors. ChemMedChem 2016, 11, 1446–1458. 10.1002/cmdc.201600137. 27159418

[ref38] Rana M.; Arif R.; Khan F. I.; Maurya V.; Singh R.; Faizan M. I.; Yasmeen S.; Dar S. H.; Alam R.; Sahu A.; Ahmad T.; Rahisuddin Pyrazoline analogs as potential anticancer agents and their apoptosis, molecular docking, MD simulation, DNA binding and antioxidant studies. Bioorg. Chem. 2021, 108, 104665 10.1016/j.bioorg.2021.104665. 33571809

[ref39] Shalaby R.; Petzer J. P.; Petzer A.; Ashraf U. M.; Atari E.; Alasmari F.; Kumarasamy S.; Sari Y.; Khalil A. SAR and molecular mechanism studies of monoamine oxidase inhibition by selected chalcone analogs. J. Enzyme Inhib. Med. Chem. 2019, 34, 863–876. 10.1080/14756366.2019.1593158. 30915862 PMC6442233

[ref40] Wei M.-X.; Yu J.-Y.; Liu X.-X.; Li X.-Q.; Zhang M.-W.; Yang P.-W.; Yang J.-H. Synthesis of artemisinin-piperazine-furan ether hybrids and evaluation of in vitro cytotoxic activity. Eur. J. Med. Chem. 2021, 215, 113295 10.1016/j.ejmech.2021.113295. 33636536

[ref41] Yu Strobykina I.; Voloshina A. D.; Andreeva O. V.; Sapunova A. S.; Lyubina A. P.; Amerhanova S. K.; Belenok M. G.; Saifina L. F.; Semenov V. E.; Kataev V. E. Synthesis, antimicrobial activity and cytotoxicity of triphenylphosphonium (TPP) conjugates of 1,2,3-triazolyl nucleoside analogues. Bioorg. Chem. 2021, 116, 105328 10.1016/j.bioorg.2021.105328. 34500307

[ref42] Hassan R. M.; Abd-Allah W. H.; Salman A. M.; El-Azzouny A. A.-S.; Aboul-Enein M. N. Design, synthesis and anticancer evaluation of novel 1,3-benzodioxoles and 1,4-benzodioxines. Eur. J. Pharm. Sci. 2019, 139, 105045 10.1016/j.ejps.2019.105045. 31421253

[ref43] Dallakyan S.; Olson A. J. Small-Molecule Library Screening by Docking with PyRx. Methods Mol. Biol. 2015, 243–250. 10.1007/978-1-4939-2269-7_19. 25618350

[ref44] Trott O.; Olson A. J. AutoDock Vina: Improving the speed and accuracy of docking with a new scoring function, efficient optimization, and multithreading. J. Comput. Chem. 2010, 31, 455–461. 10.1002/jcc.21334. 19499576 PMC3041641

[ref45] Rasool F.; Khalid M.; Yar M.; Ayub K.; Tariq M.; Hussain A.; Lateef M.; Kashif M.; Iqbal S. Facile synthesis, DNA binding, Urease inhibition, anti-oxidant, molecular docking and DFT studies of 3-(3-Bromo-phenyl)-1-(2-trifluoromethyl-phenyl)-propenone and 3-(3-Bromo-5 chloro-phenyl)-1-(2-trifluoromethyl-phenyl)-propenone. J. Mol. Liq. 2021, 336, 116302 10.1016/j.molliq.2021.116302.

[ref46] Walters W. P.; Murcko M. A. Prediction of ‘drug-likeness’. Adv. Drug Delivery Rev. 2002, 54, 255–271. 10.1016/s0169-409x(02)00003-0. 11922947

[ref47] Nehra N.; Tittal R. K.; Ghule V. D. 1,2,3-Triazoles of 8-Hydroxyquinoline and HBT: Synthesis and Studies (DNA Binding, Antimicrobial, Molecular Docking, ADME, and DFT). ACS Omega 2021, 6, 27089–27100. 10.1021/acsomega.1c03668. 34693129 PMC8529673

[ref48] Vadivel M.; Aravinda T.; Swamynathan K.; Kumar B. V.; Kumar S. DNA binding activity of novel discotic phenathridine derivative. J. Mol. Liq. 2021, 332, 115798 10.1016/j.molliq.2021.115798.

[ref49] Patel M.; Chhasatia M.; Parmar P. Antibacterial and DNA interaction studies of zinc(II) complexes with quinolone family member, ciprofloxacin. Eur. J. Med. Chem. 2010, 45, 439–446. 10.1016/j.ejmech.2009.10.024. 19913957

[ref50] Rajalakshmi S.; Weyhermüller T.; Dinesh M.; Nair B. U. Copper(II) complexes of terpyridine derivatives: A footstep towards the development of antiproliferative agent for breast cancer. J. Inorg. Biochem. 2012, 117, 48–59. 10.1016/j.jinorgbio.2012.08.010. 23078774

[ref51] El-Sonbati A. Z.; Diab M. A.; Morgan S. M. Thermal properties, antimicrobial activity and DNA binding of Ni(II) complexes of azo dye compounds. J. Mol. Liq. 2017, 225, 195–206. 10.1016/j.molliq.2016.11.047.

[ref52] Morgan S. M.; Diab M. A.; El-Sonbati A. Z. Synthesis, spectroscopic, thermal properties, Calf thymus DNA binding and quantum chemical studies of M(II) complexes. Appl. Organomet. Chem. 2018, 32, e4281 10.1002/aoc.4281.

[ref54] Akram M.; Lal H.; Kabir-ud-Din Exploring the binding mode of ester-based cationic gemini surfactants with calf thymus DNA: A detailed physicochemical, spectroscopic and theoretical study. Bioorg. Chem. 2022, 119, 105555 10.1016/j.bioorg.2021.105555. 34923244

[ref55] Xi P.-x.; Xu Z.-h.; Liu X.-h.; Cheng F.-j.; Zeng Z.-z. Synthesis, characterization, and DNA-binding studies of 1-cyclohexyl-3-tosylurea and its Ni(II), and Cd(II) complexes. Spectrochim. Acta, Part A 2008, 71, 523–528. 10.1016/j.saa.2008.01.005. 18280777

[ref56] Pešić M.; Bugarinovic J.; Minic A.; Novakovic S. B.; Bogdanovic G. A.; Todosijevic A.; Stevanovic D.; Damljanovic I. Electrochemical characterization and estimation of DNA-binding capacity of a series of novel ferrocene derivatives. Bioelectrochemistry 2020, 132, 107412 10.1016/j.bioelechem.2019.107412. 31889632

[ref57] Chung C.-C.; Chung C. W.; Yuan C. P. Excited-state vibrational relaxation and deactivation dynamics of trans-4-(N, N-dimethylamino)-40-nitrostilbene in nonpolar solvents studied by ultrafast time-resolved broadband fluorescence spectroscopy. J. Photochem. Photobiol., A 2015, 310, 26–32. 10.1016/j.jphotochem.2015.05.023.

[ref58] Tripathi A. K. Binding interaction of N-acetylated acridine conjugate with ct-DNA and β-cyclodextrin: synthesis and photophysical studies. J. Photochem. Photobiol., A 2018, 205, 497–502. 10.1016/j.saa.2018.07.069. 30059876

[ref59] Zhu Z.; Li W.; Yang C. Switching monomer/excimer ratiometric fluorescence to time-resolved excimer probe for DNA detection: a simple strategy to enhance the sensitivity. Sens. Actuators, B 2016, 224, 31–36. 10.1016/j.snb.2015.10.004.

[ref60] Kashid B. B.; Salunkhe P. H.; Dongare B. B.; More K. R.; Khedkar V. M.; Ghanwat A. A. Synthesis of novel of 2, 5-disubstituted 1, 3, 4- oxadiazole derivatives and their in vitro anti-inflammatory, anti-oxidant evaluation, and molecular docking study. Bioorg. Med. Chem. Lett. 2020, 30, 127136 10.1016/j.bmcl.2020.127136. 32280025

[ref62] Chen K.; Zhang Y.-L.; Fan J.; Ma X.; Qin Y.-J.; Zhu H.-L. Novel nicotinoyl pyrazoline derivates bearing N-methyl indole moiety as antitumor agents: Design, synthesis and evaluation. Eur. J. Med. Chem. 2018, 156, 722–737. 10.1016/j.ejmech.2018.07.044. 30041136

[ref63] Burmaoglu S.; Gobek A.; Aydin B. O.; Yurtoglu E.; Aydin B. N.; Ozkat G. Y.; Hepokur C.; Ozek N. S.; Aysin F.; Altundas R.; Algul O. Design, synthesis and biological evaluation of novel bischalcone derivatives as potential anticancer agents. Bioorg. Chem. 2021, 111, 104882 10.1016/j.bioorg.2021.104882. 33839582

[ref64] Aneja B.; Arif R.; Perwez A.; Napoleon J. V.; Hasan P.; Rizvi M. M. A.; Azam A.; Rahisuddin M.; Abid M. Abid, N-Substituted 1,2,3-Triazolyl-Appended Indole-Chalcone Hybrids as Potential DNA Intercalators Endowed with Antioxidant and Anticancer Properties. ChemistrySelect 2018, 3, 2638–2645. 10.1002/slct.201702913.

[ref65] Yang C.-Z.; Liang C.-Y.; Zhang D.; Hu Y.-J. Deciphering the interaction of methotrexate with DNA: Spectroscopic and molecular docking study. J. Mol. Liq. 2017, 248, 1–6. 10.1016/j.molliq.2017.10.017.

[ref66] Hassan N. W.; Saudi M. N.; Abdel-Ghany Y. S.; Ismail A.; Elzahhar P. A.; Sriram D.; Nassra R.; Abdel-Aziz M. M.; El-Hawash S. A. Novel pyrazine based anti-tubercular agents: Design, synthesis, biological evaluation and in silico studies. Bioorg. Chem. 2020, 96, 103610 10.1016/j.bioorg.2020.103610. 32028062

[ref67] Pyle A. M.; Rehmann J. P.; Meshoyrer R.; Kumar C. V.; Turro N. J.; Barton J. K. Mixed-ligand complexes of ruthenium(II): factors governing binding to DNA. J. Am. Chem. Soc. 1989, 111, 3051–3058. 10.1021/ja00190a046.

[ref68] Kovvuri J.; Nagaraju B.; Nayak V. L.; Akunuri R.; Rao M. P. N.; Ajitha A.; Nagesh N.; Kamal A. Design, synthesis and biological evaluation of new β-carboline-bisindole compounds as DNA binding, photocleavage agents and topoisomerase I inhibitors. Eur. J. Med. Chem. 2018, 143, 1563–1577. 10.1016/j.ejmech.2017.10.054. 29129513

[ref69] Patel S.; Patel P.; Undre S. B.; Pandya S. R.; Singh M.; Bakshi S. DNA binding and dispersion activities of titanium dioxide nanoparticles with UV/vis spectrophotometry, fluorescence spectroscopy and physicochemical analysis at physiological temperature. J. Mol. Liq. 2016, 213, 304–311. 10.1016/j.molliq.2015.11.002.

[ref70] Dehkordi M. F.; Farhadian S.; Abdolvand M.; Soureshjani E. H.; Rahmani B.; Darzi S. Deciphering the DNA-binding affinity, cytotoxicity, and apoptosis induce as the anticancer mechanism of Bavachinin: An experimental and computational investigation. J. Mol. Liq. 2021, 341, 117373 10.1016/j.molliq.2021.117373.

[ref71] Zia M.; Hameed S.; Ahmad I.; Tabassum N.; Yousuf S. Regio-isomeric isoxazole sulfonates: Synthesis, characterization, electrochemical studies, and DNA binding activity. J. Mol. Struct. 2020, 1220, 128635 10.1016/j.molstruc.2020.128635.

[ref72] Chiu C.-C.; Chen W.-C.; Cheng P.-Y. Excited-state vibrational relaxation and deactivation dynamics of trans-4-(N, N-dimethylamino)-4′-nitrostilbene in nonpolar solvents studied by ultrafast time-resolved broadband fluorescence spectroscopy. J. Photochem. Photobiol., A 2015, 310, 26–32. 10.1016/j.jphotochem.2015.05.023.

[ref73] Ansari I. A.; Sama F.; Shahid M.; Rahisuddin R.; Arif R.; Khalid M.; Siddiqi Z. A. Isolation of proton transfer complexes containing 4-picolinium as cation and pyridine-2,6-dicarboxylate complex as anion: Crystallographic and spectral investigations, antioxidant activities and molecular docking studies. RSC Adv. 2016, 6, 11088–11098. 10.1039/c5ra25939h.

