# Correction to “Design and Synthesis of Carbothioamide/Carboxamide-Based Pyrazoline Analogs as Potential Anticancer Agents: Apoptosis, Molecular Docking, ADME Assay, and DNA Binding Studies”

**DOI:** 10.1021/acsomega.2c05261

**Published:** 2022-08-29

**Authors:** Manish Rana, Md Imam Faizan, Sajad Hussain Dar, Tanveer Ahmad, Rahisuddin

Scheme 1 on page no. 22642 needs to be replaced with the version given here.

Equation 2 in section 2.7 on page no. 22643 needs to be replaced as
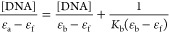
2where the extinction coefficient of the compound at each DNA concentration, the extinction coefficient of the drug–DNA complex in the bound form, and the extinction coefficient for the drug are represented as ε_a_, ε_b_, and ε_f_, respectively.

**Scheme 1:**
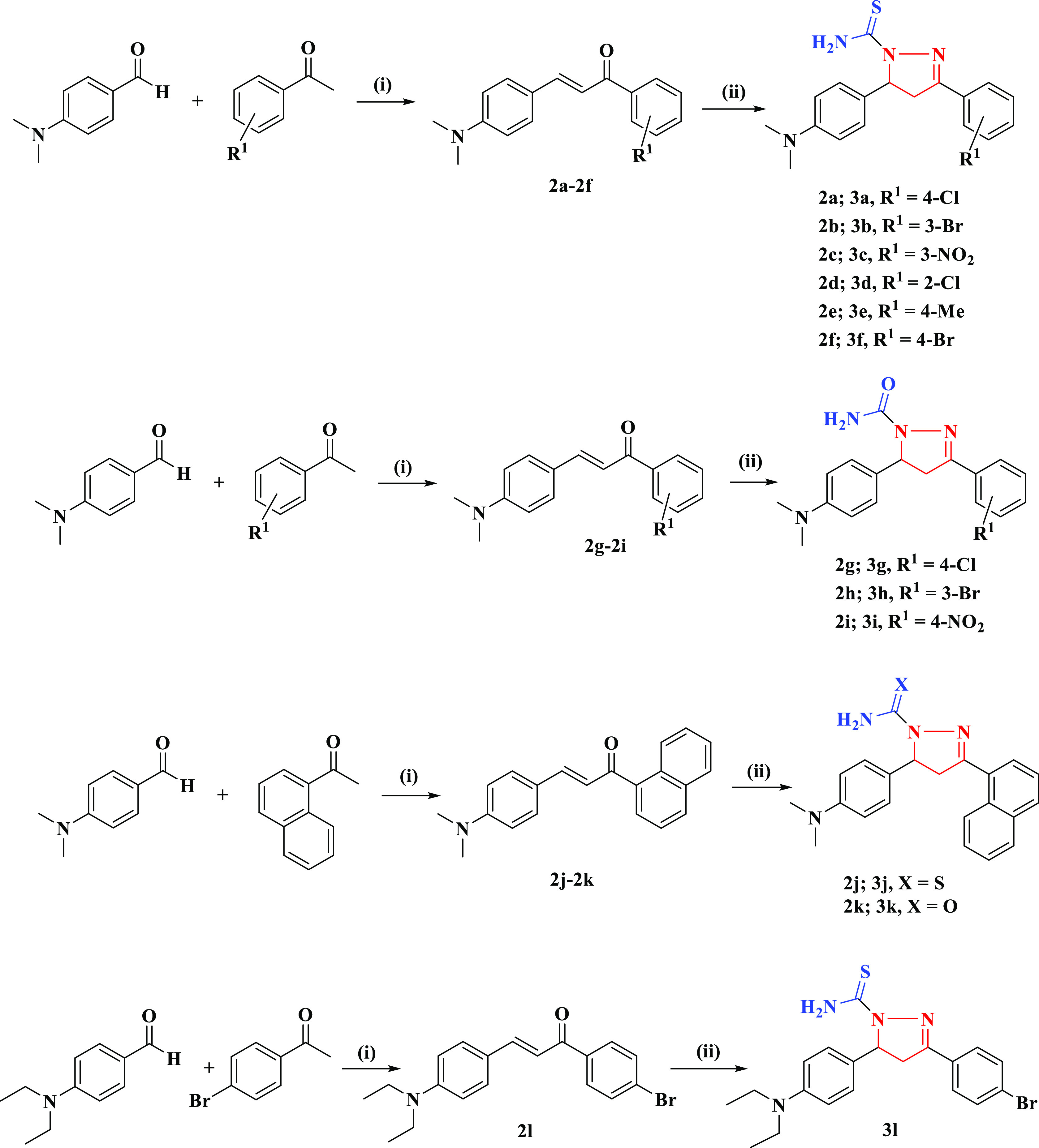
Preparations of Pyrazoline Derivatives (**3a**–**3l**) (i) NaOH (50%), absolute ethanol, stir; (ii) thiosemicarbazide/semicarbazide, reflux for 4−6 h.

