# Supplementary material for: Design and Synthesis of Carbothioamide/Carboxamide-Based Pyrazoline Analogs as Potential Anticancer Agents: Apoptosis, Molecular Docking, ADME Assay, and DNA Binding Studies
Source: ACS Omega. 2022 Jun 23;7(26):22639–56. doi: 10.1021/acsomega.2c02033 (PMC9260921; doi:10.1021/acsomega.2c02033)
Supplement: Supplementary file 1 — ao2c02033_si_001.pdf [file ao2c02033_si_001.pdf]

Design and Synthesis of Carbothioamide/Carboxamide-Based Pyrazoline Analogs as Potential Anticancer Agents: Apoptosis, Molecular Docking, ADME Assay, and DNA Binding Studies

Manish Rana[a], Md Imam Faizan[b], Sajad Hussain Dar[a], Tanveer Ahmad[b] and Rahisuddin[a,*]

[a]*Department of Chemistry, Jamia Millia Islamia, New Delhi 110025, INDIA,*

[b]*Multidisciplinary Centre for Advanced Research & Studies, Jamia Millia Islamia, New Delhi110025, INDIA*

*Author for correspondence:

rahisuddin@jmi.ac.in (+919871460479)

Contents

1. NMR spectra of products……………………………………………S1
2. IR spectra of products……………………………………………..S2
3. Mass spectra of products……………………………………………S3
4. HPLC spectra of products……………………………………………S4
5. HRMS of lead compounds……………………………………………S5

3a

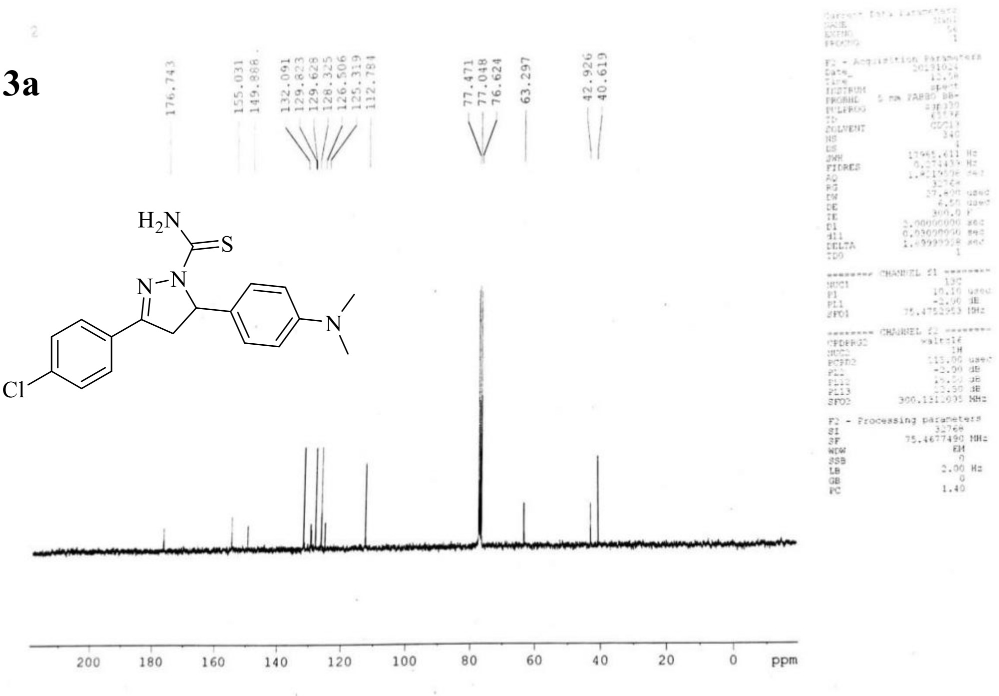

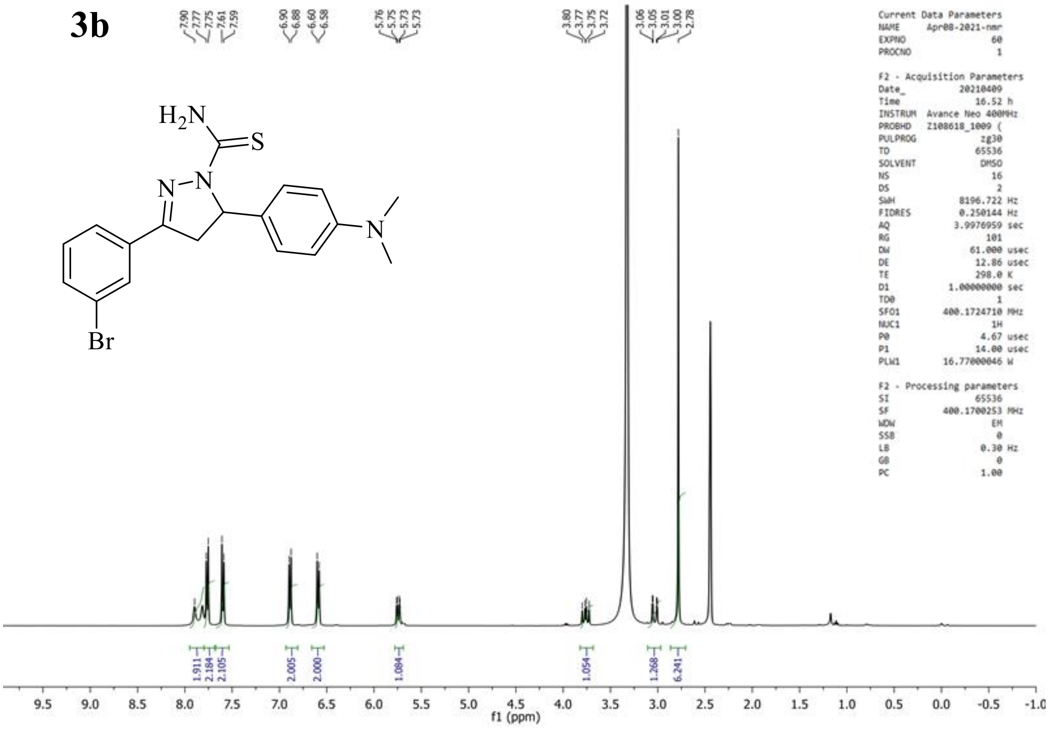
3b
7.90 7.77 7.75 7.61 7.59
6.90 6.88 6.60 6.58
5.76 5.75 5.73 5.73
3.80 3.77 3.75 3.72
3.06 3.05 3.01 3.00 2.78
H2N
S
N
N
N
Br
1.911 2.184 2.105
2.005 2.000
1.084
1.054
1.268 6.241
f1 (ppm)
Current Data Parameters
NAME Apr08-2021-nmr
EXPNO 60
PROCNO 1
F2 - Acquisition Parameters
Date_ 20210409
Time 16.52 h
INSTRUM Avance Neo 400MHz
PULPROG zg30
TD 65536
SOLVENT DMSO
NS 16
DS 2
SWH 8196.722 Hz
FIDRES 0.250144 Hz
AQ 3.9976959 sec
RG 101
DW 61.000 usec
DE 12.86 usec
TE 298.0 K
D1 1.00000000 sec
TD0 1
SFO1 400.1724710 MHz
NUC1 1H
P0 4.67 usec
P1 14.00 usec
PLW1 16.77000046 W
F2 - Processing parameters
SI 65536
SF 400.1700253 MHz
WDW EM
SSB 0
LB 0.30 Hz
GB 0
PC 1.00

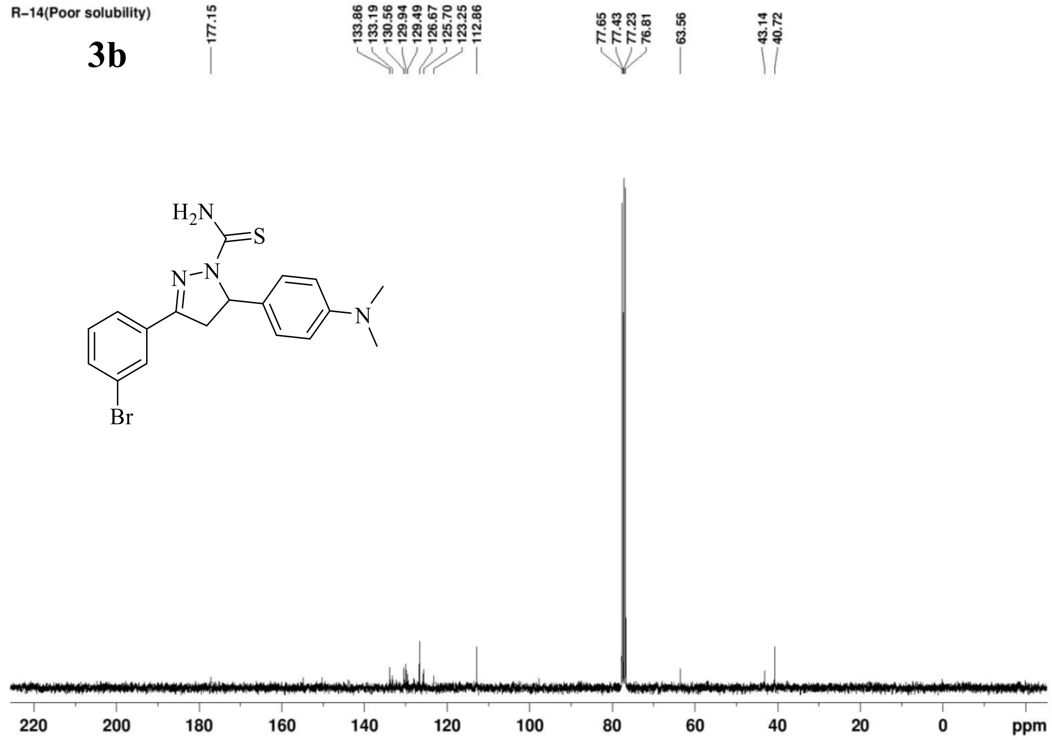
R-14(Poor solubility)
3b
177.15
133.86 133.19 130.56 129.94 129.49 126.67 125.70 123.25 112.86
77.65 77.43 77.23 76.81
63.56
43.14 40.72
H2N
S
N
N
N
Br
ppm

3c

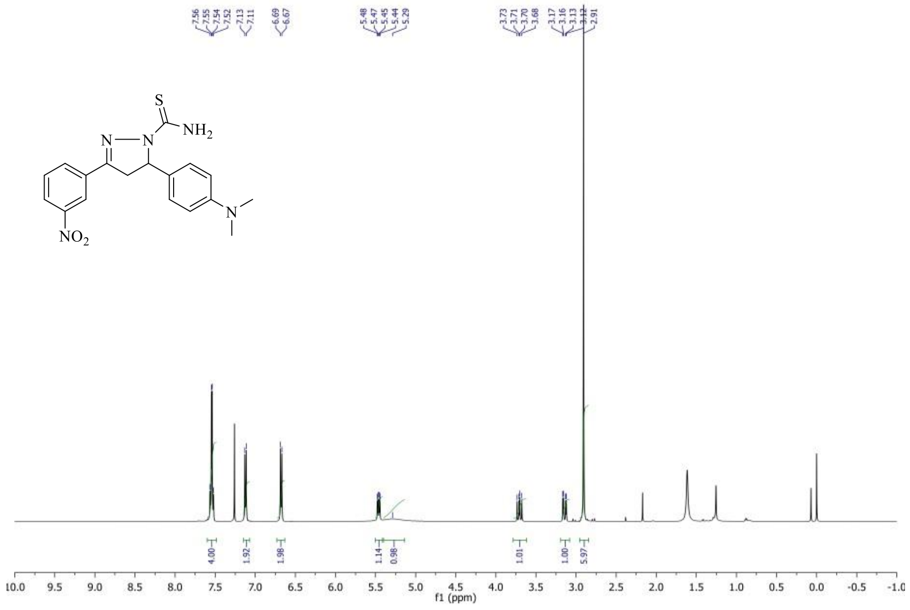

3c

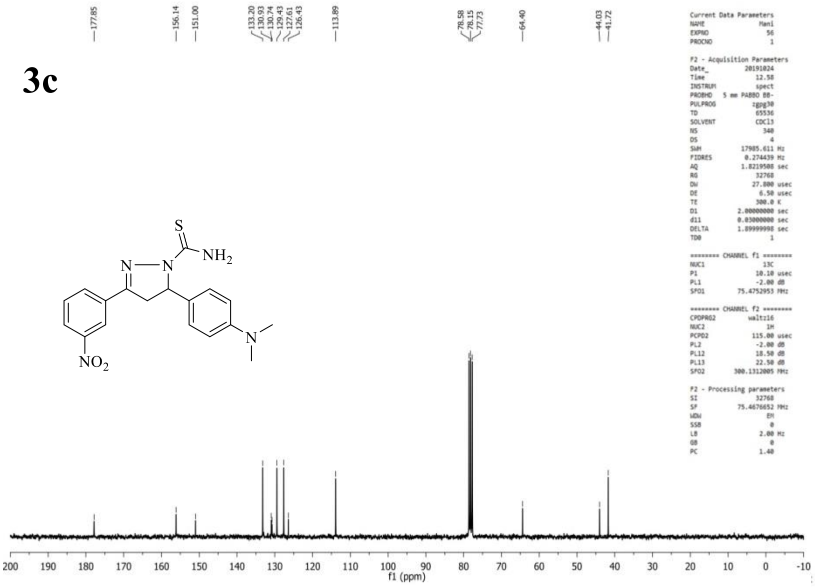

3d

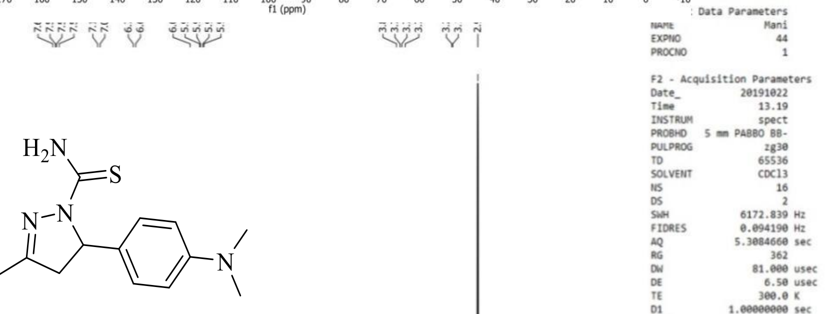

Data Parameters
NAME Mani
EXPNO 44
PROCNO 1
F2 - Acquisition Parameters
Date_ 20191022
Time 13.19
INSTRUM spect
PROBHD 5 mm PABBO BB-
PULPROG zg30
TD 65536
SOLVENT CDCl3
NS 16
DS 2
SWH 6172.839 Hz
FIDRES 0.094190 Hz
AQ 5.3084660 sec
RG 362
DW 81.000 usec
DE 6.50 usec
TE 300.0 K
D1 1.00000000 sec

3d

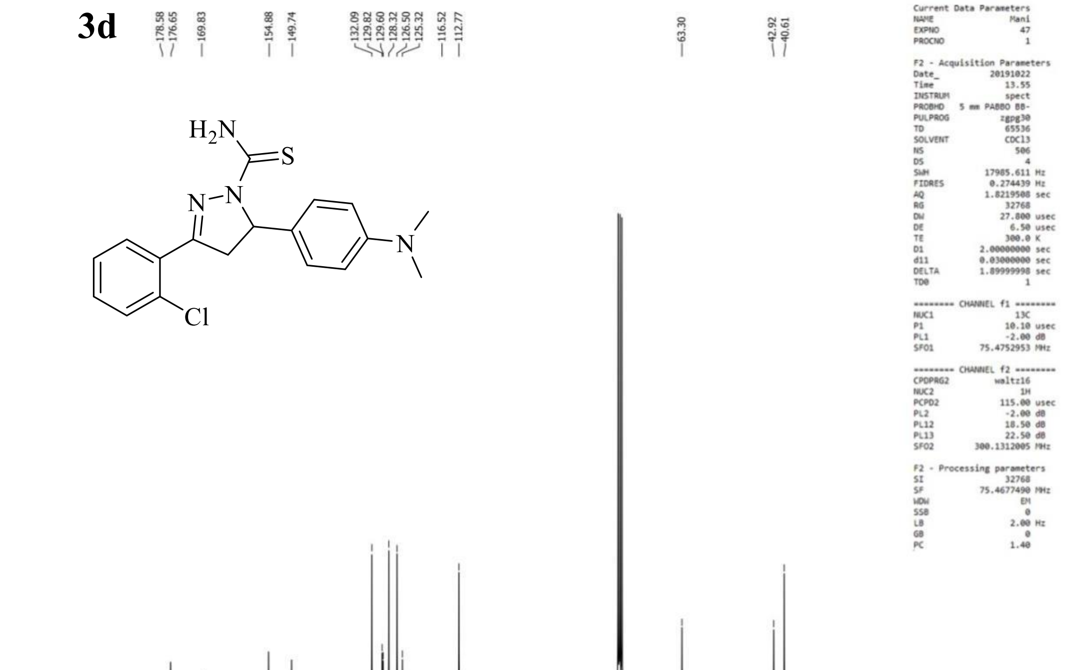

178.58
176.65
169.83
154.88
149.74
132.09
129.82
129.60
128.32
126.50
125.32
116.52
112.77
63.30
42.92
40.61
H2N
S
N
N
N
Cl
Current Data Parameters
NAME Mani
EXPNO 47
PROCNO 1
F2 - Acquisition Parameters
Date_ 20191022
Time 13.55
INSTRUM spect
PROBHD 5 mm PABBO BB-
PULPROG zgpg30
TD 65536
SOLVENT CDCl3
NS 506
DS 4
SWH 17985.611 Hz
FIDRES 0.274439 Hz
AQ 1.8219508 sec
RG 32768
DW 27.800 usec
DE 6.50 usec
TE 300.0 K
D1 2.00000000 sec
d11 0.03000000 sec
DELTA 1.89999998 sec
TD0 1
======== CHANNEL f1 ========
NUC1 13C
P1 10.10 usec
PL1 -2.00 dB
SFO1 75.4752953 MHz
======== CHANNEL f2 ========
CPDPRG2 waltz16
NUC2 1H
PCPD2 115.00 usec
PL2 -2.00 dB
PL12 18.50 dB
PL13 22.50 dB
SFO2 300.1312005 MHz
F2 - Processing parameters
SI 32768
SF 75.4677490 MHz
WDW EM
SSB 0
LB 2.00 Hz
GB 0
PC 1.40
210 200 190 180 170 160 150 140 130 120 110 100 90 80 70 60 50 40 30 20 10 0 -10
f1 (ppm)

3e

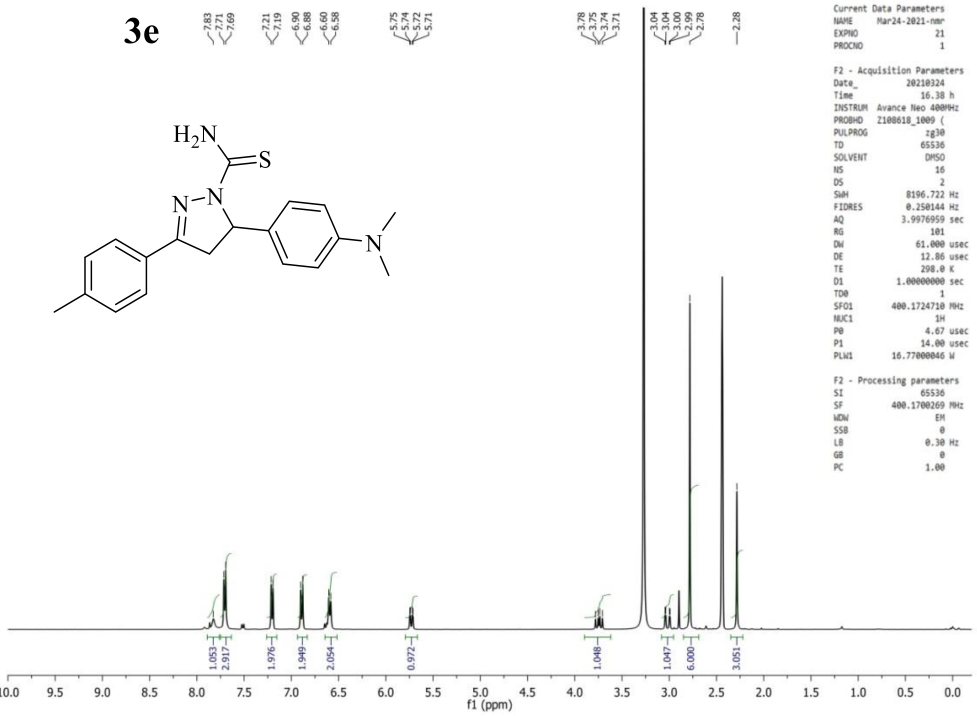

7.83
7.71
7.69
7.21
7.19
6.90
6.88
6.60
6.58
5.75
5.74
5.72
5.71
3.78
3.75
3.74
3.71
3.04
3.04
3.00
2.99
2.78
2.28
H_2N
S
N
N
N
1.053
2.917
1.976
1.949
2.054
0.972
1.048
1.047
6.000
3.051
10.0 9.5 9.0 8.5 8.0 7.5 7.0 6.5 6.0 5.5 5.0 4.5 4.0 3.5 3.0 2.5 2.0 1.5 1.0 0.5 0.0
f1 (ppm)
Current Data Parameters
NAME Mar24-2021-nmr
EXPNO 21
PROCNO 1
F2 - Acquisition Parameters
Date_ 20210324
Time 16.38 h
INSTRUM Avance Neo 400MHz
PROBHD Z108618_1009 (
PULPROG zg30
TD 65536
SOLVENT DMSO
NS 16
DS 2
SWH 8196.722 Hz
FIDRES 0.250144 Hz
AQ 3.9976959 sec
RG 101
DW 61.000 usec
DE 12.86 usec
TE 298.0 K
D1 1.00000000 sec
TD0 1
SFO1 400.1724710 MHz
NUC1 1H
P0 4.67 usec
P1 14.00 usec
PLW1 16.77000046 W
F2 - Processing parameters
SI 65536
SF 400.1700269 MHz
WDW EM
SSB 0
LB 0.30 Hz
GB 0
PC 1.00

3e

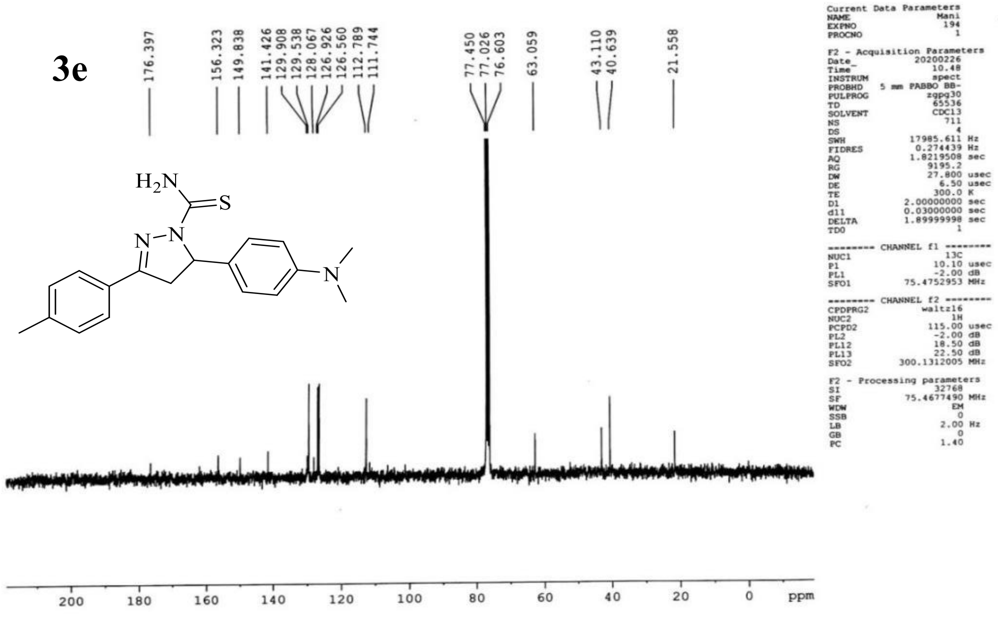

176.397
156.323
149.838
141.426
129.908
129.538
128.067
126.926
126.560
112.789
111.744
77.450
77.026
76.603
63.059
43.110
40.639
21.558
H_2N
S
N
N
N
200 180 160 140 120 100 80 60 40 20 0 ppm
Current Data Parameters
NAME Mani
EXPNO 194
PROCNO 1
F2 - Acquisition Parameters
Date_ 20200226
Time 10.48
INSTRUM spect
PROBHD 5 mm PABBO BB-
PULPROG zgpg30
TD 65536
SOLVENT CDCl3
NS 711
DS 4
SWH 17985.611 Hz
FIDRES 0.274439 Hz
AQ 1.8219508 sec
RG 9195.2
DW 27.800 usec
DE 6.50 usec
TE 300.0 K
D1 2.00000000 sec
d11 0.03000000 sec
DELTA 1.89999998 sec
TD0 1
======== CHANNEL f1 ========
NUC1 13C
P1 10.10 usec
PL1 -2.00 dB
SFO1 75.4752953 MHz
======== CHANNEL f2 ========
CPDPRG2 waltz16
NUC2 1H
PCPD2 115.00 usec
PL2 -2.00 dB
PL12 18.50 dB
PL13 22.50 dB
SFO2 300.1312005 MHz
F2 - Processing parameters
SI 32768
SF 75.4677490 MHz
WDW EM
SSB 0
LB 2.00 Hz
GB 0
PC 1.40

3f

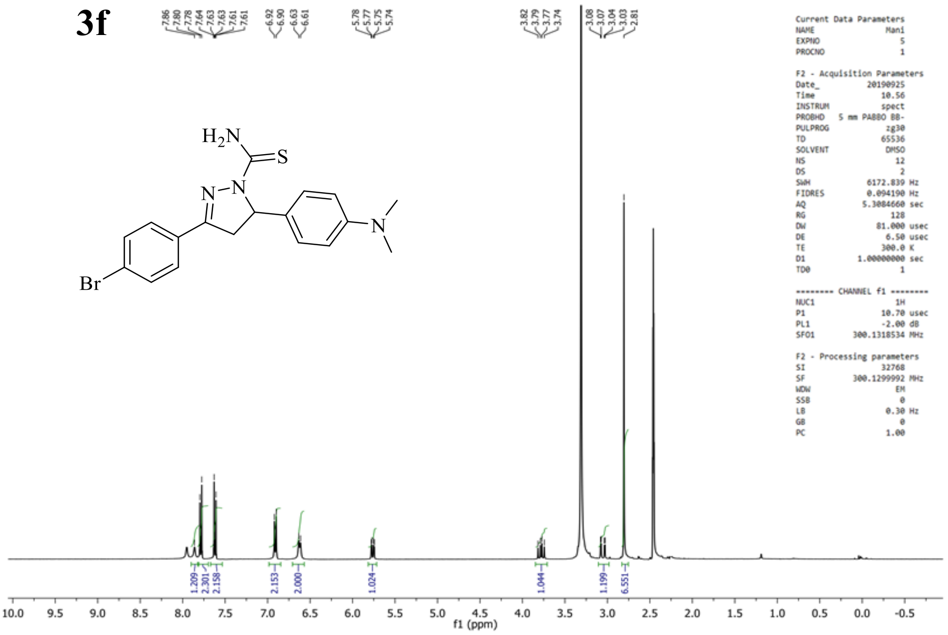

7.86
7.80
7.78
7.64
7.63
7.63
7.61
7.61
6.92
6.90
6.63
6.61
5.78
5.77
5.75
5.74
3.82
3.79
3.77
3.74
3.08
3.07
3.04
3.03
2.81
H_2N
S
N
N
N
Br
1.209
2.301
2.158
2.153
2.000
1.024
1.044
1.199
6.551
f1 (ppm)
Current Data Parameters
NAME Mani
EXPNO 5
PROCNO 1
F2 - Acquisition Parameters
Date_ 20190925
Time 10.56
INSTRUM spect
PROBHD 5 mm PABBO BB-
PULPROG zg30
TD 65536
SOLVENT DMSO
NS 12
DS 2
SWH 6172.839 Hz
FIDRES 0.094190 Hz
AQ 5.3084660 sec
RG 128
DW 81.000 usec
DE 6.50 usec
TE 300.0 K
D1 1.00000000 sec
TD0 1
======== CHANNEL f1 ========
NUC1 1H
P1 10.70 usec
PL1 -2.00 dB
SFO1 300.1318534 MHz
F2 - Processing parameters
SI 32768
SF 300.1299992 MHz
WDW EM
SSB 0
LB 0.30 Hz
GB 0
PC 1.00

3f

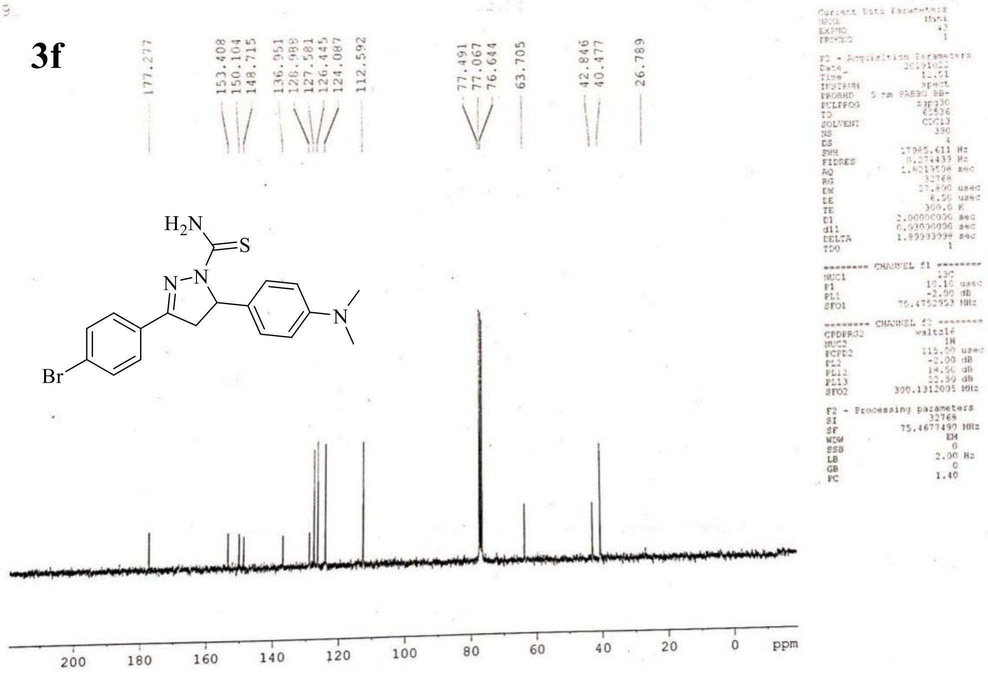

177.277
153.408
150.104
148.715
136.951
128.988
127.581
126.445
124.087
112.592
77.491
77.067
76.644
63.705
42.846
40.477
26.789
H_2N
S
N
N
N
Br
ppm

3g

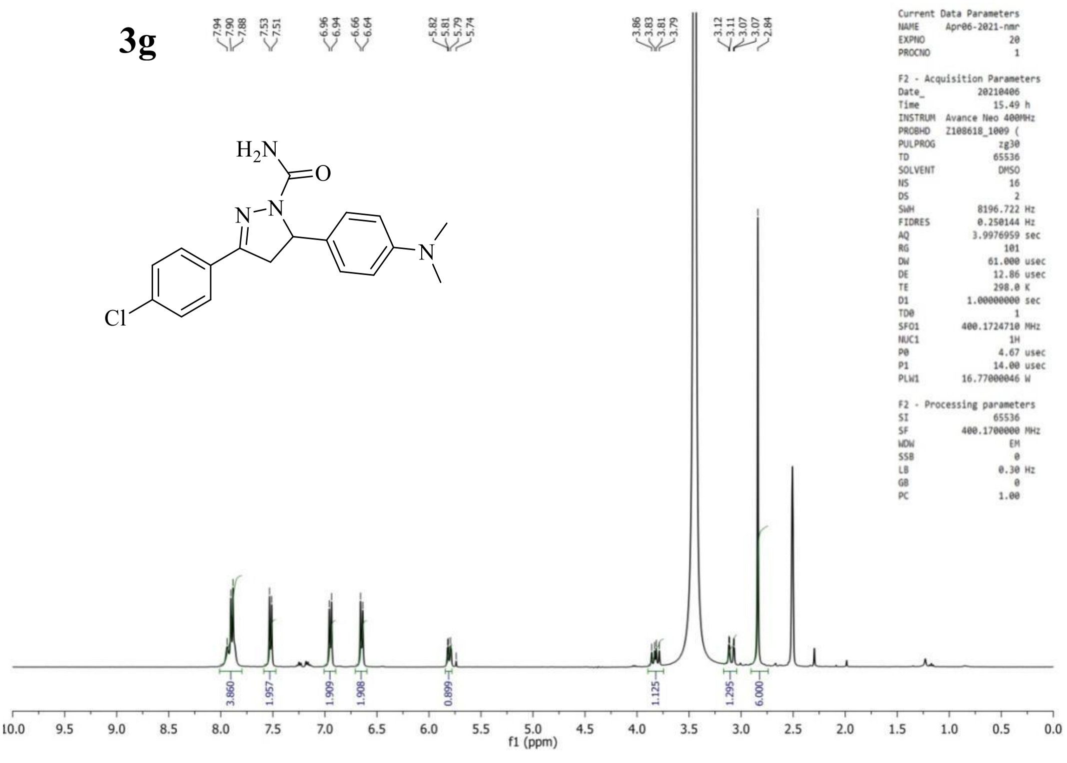
7.94
7.90
7.88
7.53
7.51
6.96
6.94
6.66
6.64
5.82
5.81
5.79
5.74
3.86
3.83
3.81
3.79
3.12
3.11
3.07
3.07
2.84
H_2N
O
N
N
N
Cl
Current Data Parameters
NAME Apr06-2021-nmr
EXPNO 20
PROCNO 1
F2 - Acquisition Parameters
Date_ 20210406
Time 15.49 h
INSTRUM Avance Neo 400MHz
PROBHD Z108618_1009 (
PULPROG zg30
TD 65536
SOLVENT DMSO
NS 16
DS 2
SWH 8196.722 Hz
FIDRES 0.250144 Hz
AQ 3.9976959 sec
RG 101
DW 61.000 usec
DE 12.86 usec
TE 298.0 K
D1 1.00000000 sec
TD0 1
SFO1 400.1724710 MHz
NUC1 1H
P0 4.67 usec
P1 14.00 usec
PLW1 16.77000046 W
F2 - Processing parameters
SI 65536
SF 400.1700000 MHz
WDW EM
SSB 0
LB 0.30 Hz
GB 0
PC 1.00
3.860
1.957
1.909
1.908
0.899
1.125
1.295
6.000
10.0 9.5 9.0 8.5 8.0 7.5 7.0 6.5 6.0 5.5 5.0 4.5 4.0 3.5 3.0 2.5 2.0 1.5 1.0 0.5 0.0
f1 (ppm)

3g

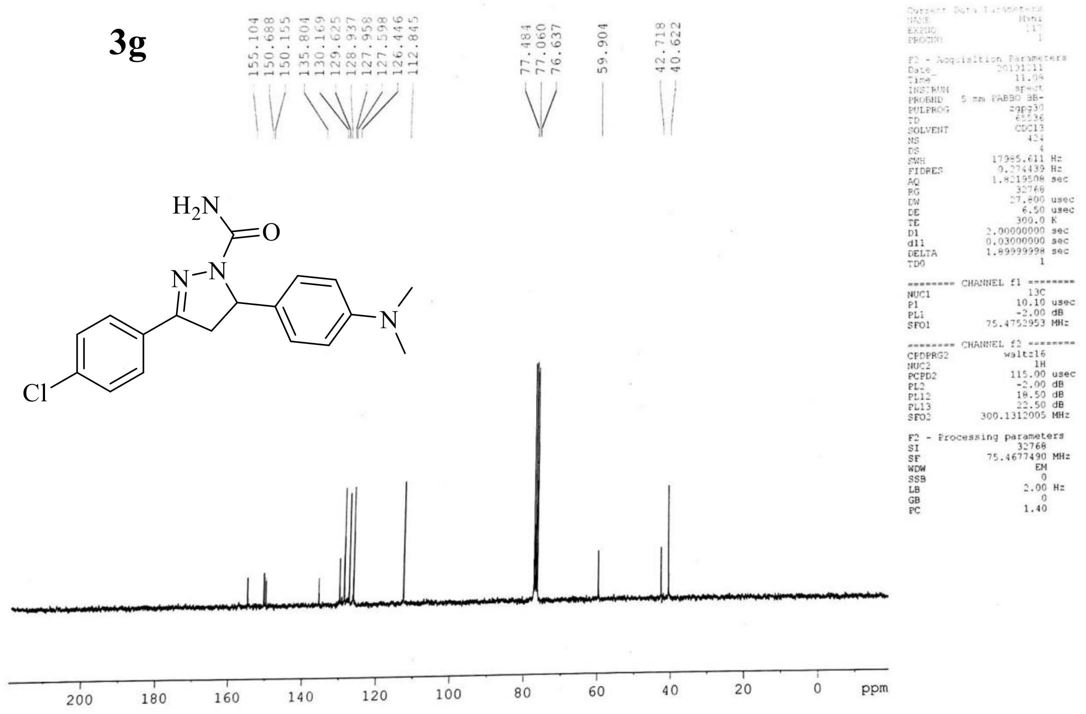
155.104
150.688
150.155
135.804
130.169
129.625
128.937
127.958
127.598
126.446
112.845
77.484
77.060
76.637
59.904
42.718
40.622
H_2N
O
N
N
N
Cl
F2 - Acquisition Parameters
SOLVENT CDCl3
NUC1 13C
SFO1 75.4752953 MHz
CPDPRG2 waltz16
NUC2 1H
SFO2 300.1312005 MHz
F2 - Processing parameters
SI 32768
SF 75.4677490 MHz
WDW EM
SSB 0
LB 2.00 Hz
GB 0
PC 1.40
200 180 160 140 120 100 80 60 40 20 0 ppm

3h

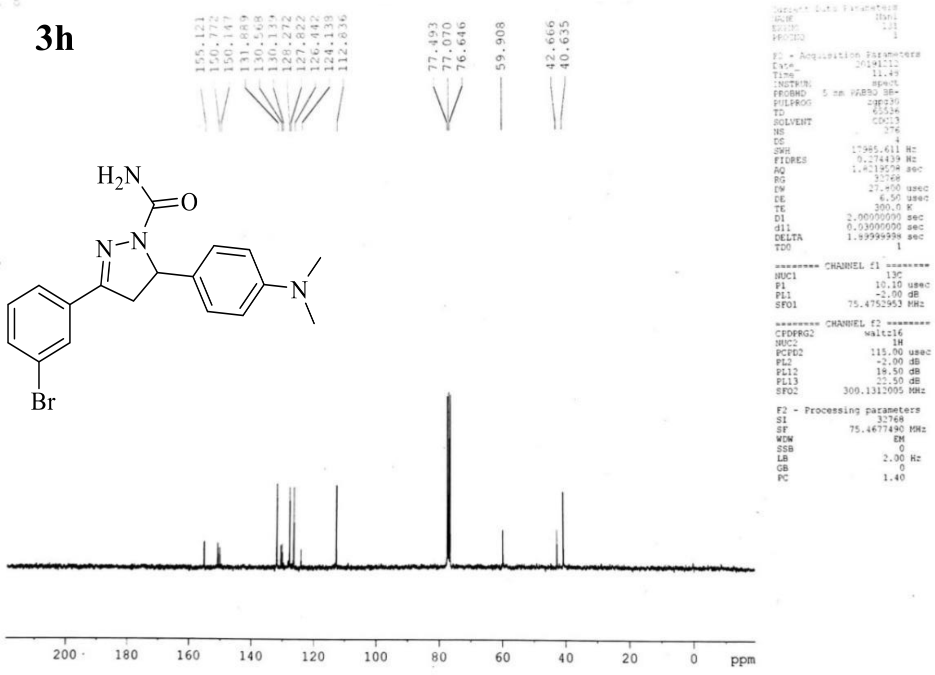

155.121
150.772
150.147
131.889
130.568
130.134
128.272
127.822
126.442
124.133
112.836
77.493
77.070
76.646
59.908
42.666
40.635
H_2N
O
N
N
N
Br
200
180
160
140
120
100
80
60
40
20
0
ppm

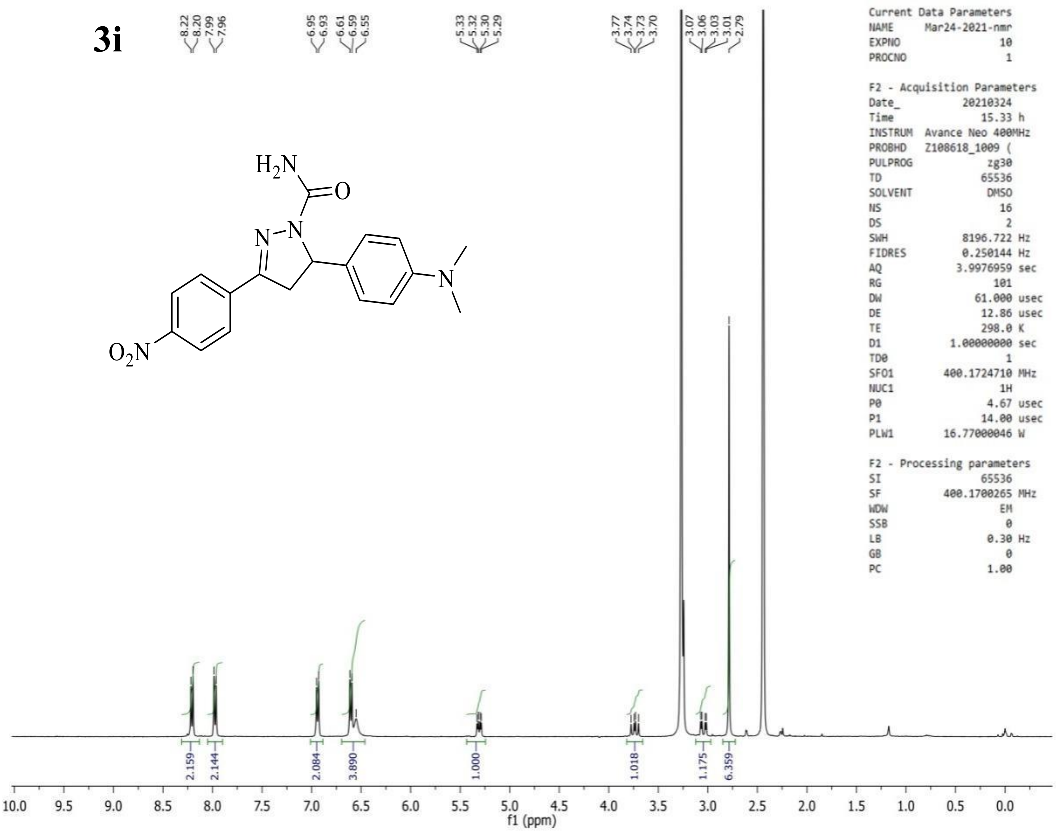
3i
8.22
8.20
7.99
7.96
6.95
6.93
6.61
6.59
6.55
5.33
5.32
5.30
5.29
3.77
3.74
3.73
3.70
3.07
3.06
3.03
3.01
2.79
H_2N
O
N–N
O_2N
N
Current Data Parameters
NAME Mar24-2021-nmr
EXPNO 10
PROCNO 1
F2 - Acquisition Parameters
Date_ 20210324
Time 15.33 h
INSTRUM Avance Neo 400MHz
PROBHD Z108618_1009 (
PULPROG zg30
TD 65536
SOLVENT DMSO
NS 16
DS 2
SWH 8196.722 Hz
FIDRES 0.250144 Hz
AQ 3.9976959 sec
RG 101
DW 61.000 usec
DE 12.86 usec
TE 298.0 K
D1 1.00000000 sec
TD0 1
SFO1 400.1724710 MHz
NUC1 1H
P0 4.67 usec
P1 14.00 usec
PLW1 16.77000046 W
F2 - Processing parameters
SI 65536
SF 400.1700265 MHz
WDW EM
SSB 0
LB 0.30 Hz
GB 0
PC 1.00
2.159
2.144
2.084
3.890
1.000
1.018
1.175
6.359
10.0 9.5 9.0 8.5 8.0 7.5 7.0 6.5 6.0 5.5 5.0 4.5 4.0 3.5 3.0 2.5 2.0 1.5 1.0 0.5 0.0
f1 (ppm)

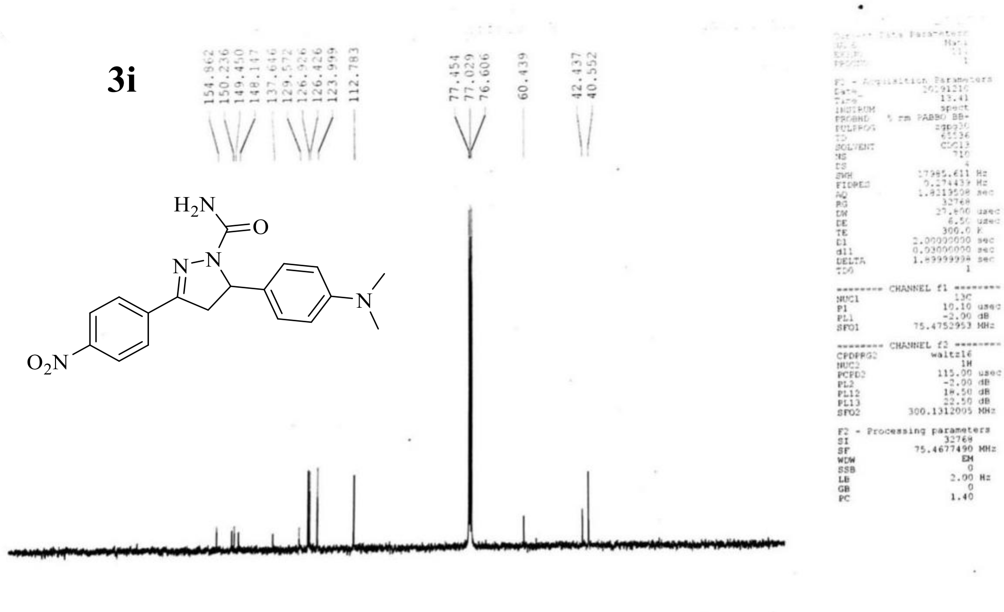
3i
154.862
150.236
149.450
148.147
137.666
129.572
126.926
126.426
123.999
112.783
77.454
77.029
76.606
60.439
42.437
40.552
H_2N
O
N–N
O_2N
N
F2 - Acquisition Parameters
SOLVENT CDCl3
CHANNEL f1
NUC1 13C
P1 10.10 usec
PL1 -2.00 dB
SFO1 75.4752953 MHz
CHANNEL f2
CPDPRG2 waltz16
NUC2 1H
PCPD2 115.00 usec
PL2 -2.00 dB
PL12 18.50 dB
PL13 22.50 dB
SFO2 300.1312005 MHz
F2 - Processing parameters
SI 32768
SF 75.4677490 MHz
WDW EM
SSB 0
LB 2.00 Hz
GB 0
PC 1.40
200 180 160 140 120 100 80 60 40 20 0 ppm

3j

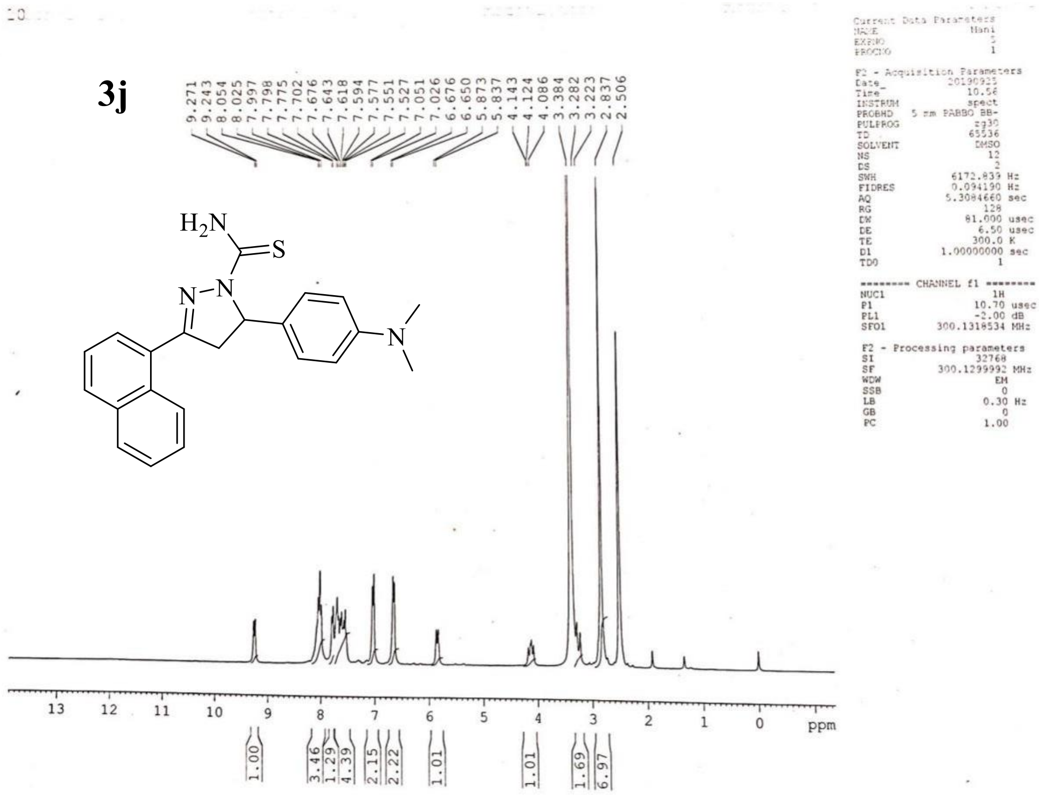
9.271
9.243
8.054
8.025
7.997
7.798
7.775
7.702
7.676
7.643
7.618
7.594
7.577
7.551
7.527
7.051
7.026
6.676
6.650
5.873
5.837
4.143
4.124
4.086
3.384
3.282
3.223
2.837
2.506
H_2N
S
N–N
N
13 12 11 10 9 8 7 6 5 4 3 2 1 0 ppm
1.00
3.46
1.29
4.39
2.15
2.22
1.01
1.01
1.69
6.97

3j

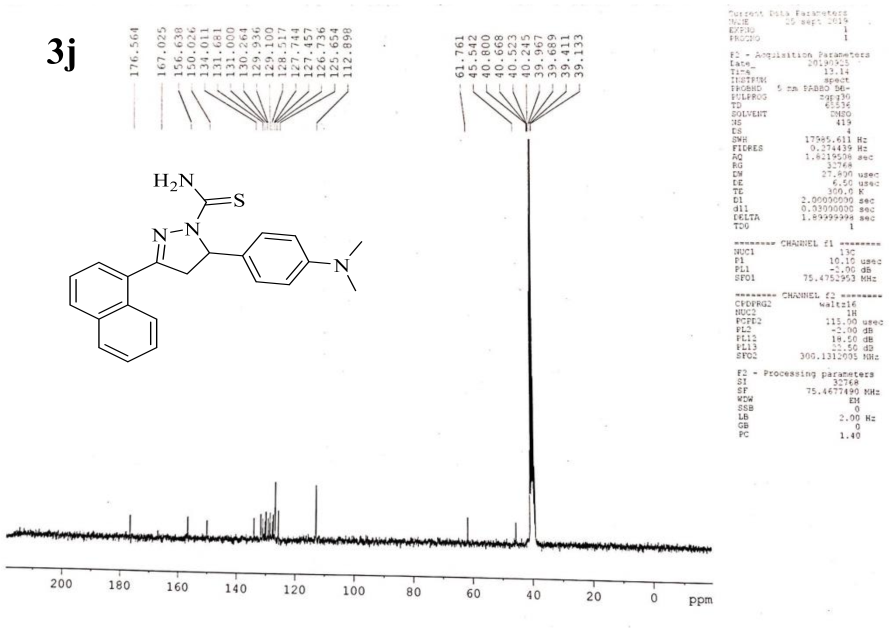
176.564
167.025
156.638
150.026
134.011
131.681
131.000
130.264
129.936
129.100
128.517
127.744
127.457
126.736
125.654
112.898
61.761
45.542
40.800
40.668
40.523
40.245
39.967
39.689
39.411
39.133
H_2N
S
N–N
N
200 180 160 140 120 100 80 60 40 20 0 ppm

3k

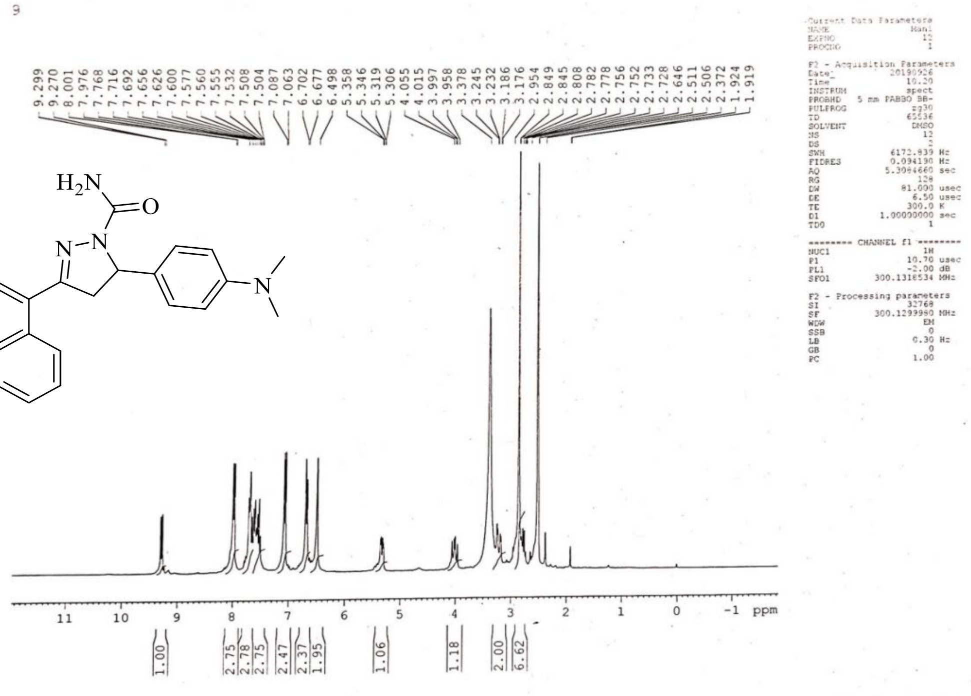
9
9.299
9.270
8.001
7.976
7.768
7.716
7.692
7.656
7.626
7.600
7.577
7.560
7.555
7.532
7.508
7.504
7.087
7.063
6.702
6.677
6.498
5.358
5.346
5.319
5.306
4.055
4.015
3.997
3.958
3.378
3.245
3.232
3.186
3.176
2.954
2.849
2.845
2.808
2.782
2.778
2.756
2.752
2.733
2.728
2.646
2.511
2.506
2.372
1.924
1.919
H_2N
O
N
N
N
Current Data Parameters
NAME Mani
EXPNO 12
PROCNO 1
F2 - Acquisition Parameters
Date_ 20190926
Time 10.20
INSTRUM spect
PROBHD 5 mm PABBO BB-
PULPROG zg30
TD 65536
SOLVENT DMSO
NS 12
DS 2
SWH 6172.839 Hz
FIDRES 0.094190 Hz
AQ 5.3084660 sec
RG 128
DW 81.000 usec
DE 6.50 usec
TE 300.0 K
D1 1.00000000 sec
TD0 1
======== CHANNEL f1 ========
NUC1 1H
P1 10.70 usec
PL1 -2.00 dB
SFO1 300.1316534 MHz
F2 - Processing parameters
SI 32768
SF 300.1299980 MHz
WDW EM
SSB 0
LB 0.30 Hz
GB 0
PC 1.00
11 10 9 8 7 6 5 4 3 2 1 0 -1 ppm
1.00
2.75
2.78
2.75
2.47
2.37
1.95
1.06
1.18
2.00
6.62

3k

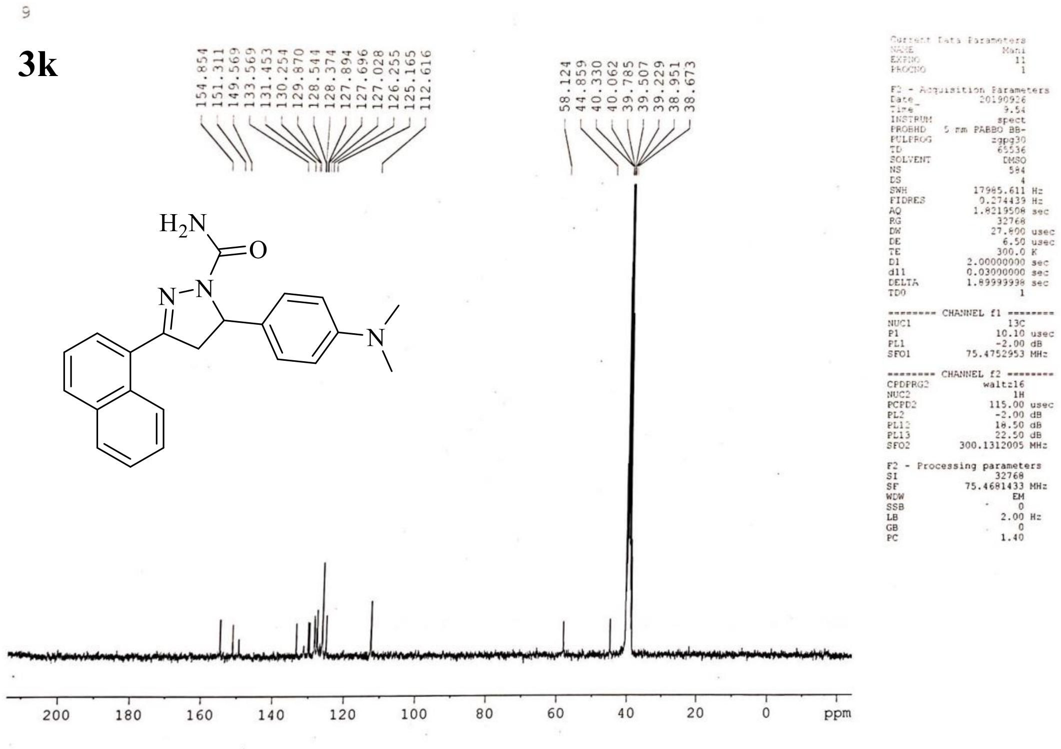
9
154.854
151.311
149.569
133.569
131.453
130.254
129.870
128.544
128.374
127.894
127.696
127.028
126.255
125.165
112.616
58.124
44.859
40.330
40.062
39.785
39.507
39.229
38.951
38.673
H_2N
O
N
N
N
Current Data Parameters
NAME Mani
EXPNO 11
PROCNO 1
F2 - Acquisition Parameters
Date_ 20190926
Time 9.54
INSTRUM spect
PROBHD 5 mm PABBO BB-
PULPROG zgpg30
TD 65536
SOLVENT DMSO
NS 584
DS 4
SWH 17985.611 Hz
FIDRES 0.274439 Hz
AQ 1.8219508 sec
RG 32768
DW 27.800 usec
DE 6.50 usec
TE 300.0 K
D1 2.00000000 sec
d11 0.03000000 sec
DELTA 1.89999998 sec
TD0 1
======== CHANNEL f1 ========
NUC1 13C
P1 10.10 usec
PL1 -2.00 dB
SFO1 75.4752953 MHz
======== CHANNEL f2 ========
CPDPRG2 waltz16
NUC2 1H
PCPD2 115.00 usec
PL2 -2.00 dB
PL12 18.50 dB
PL13 22.50 dB
SFO2 300.1312005 MHz
F2 - Processing parameters
SI 32768
SF 75.4681433 MHz
WDW EM
SSB 0
LB 2.00 Hz
GB 0
PC 1.40
200 180 160 140 120 100 80 60 40 20 0 ppm

3l

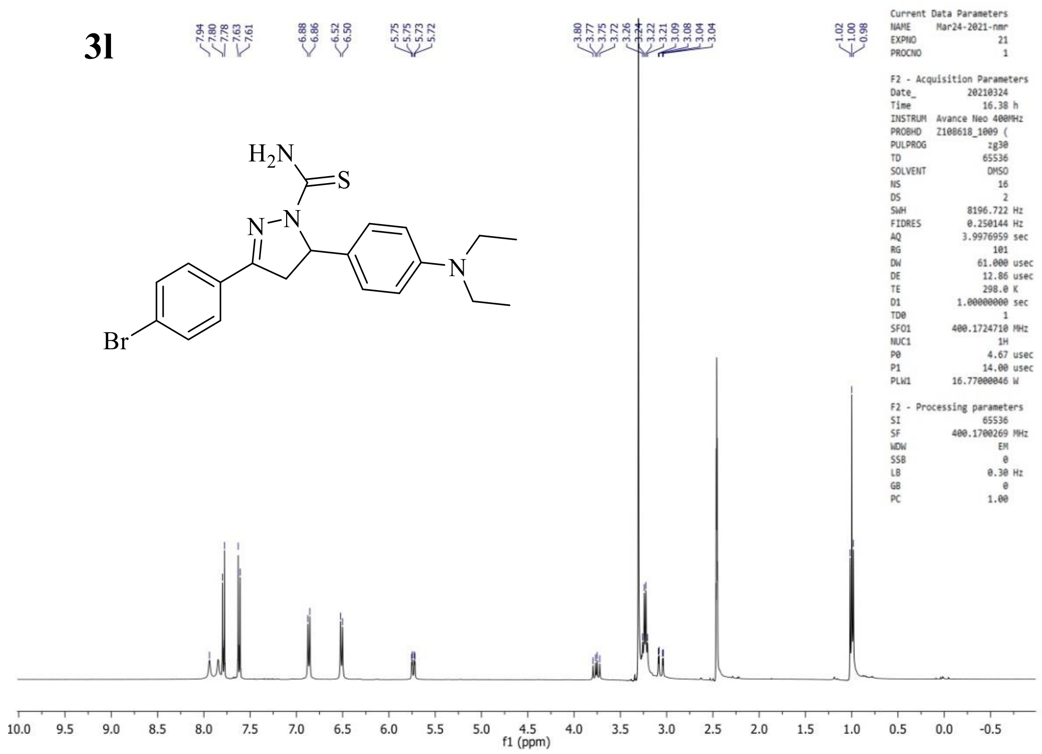

3l

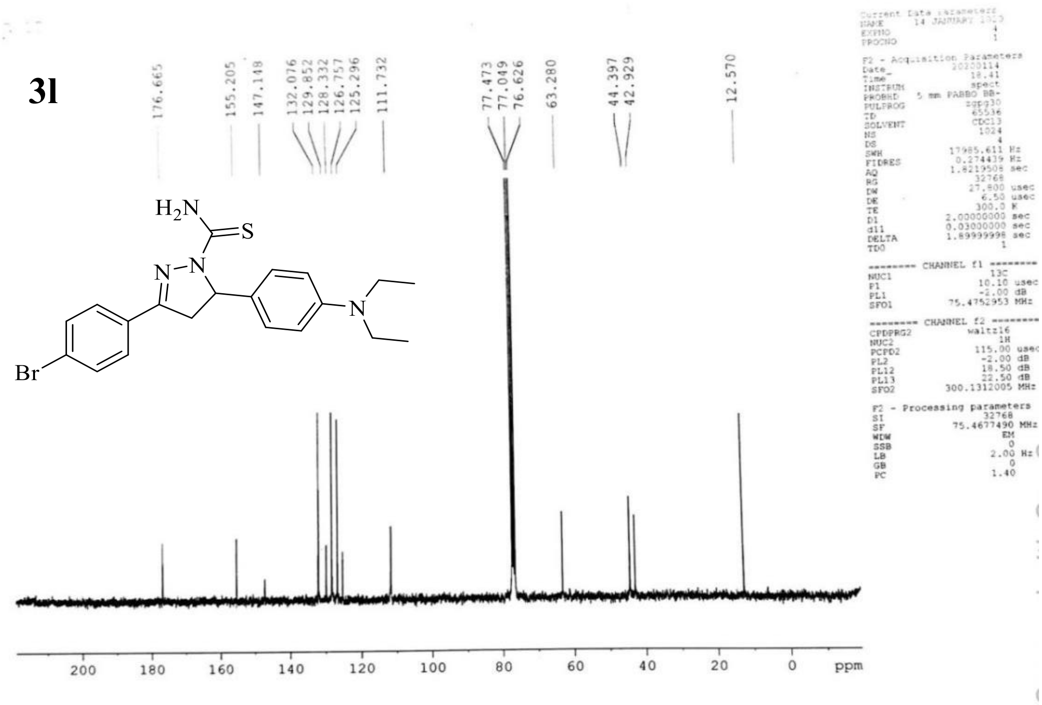

Figure. S1. ^{1}H NMR spectra of pyrazoline derivatives (3b-3g and 3i-3l) and ^{13}C-NMR spectra of derivatives 3a-3l.

(3a)

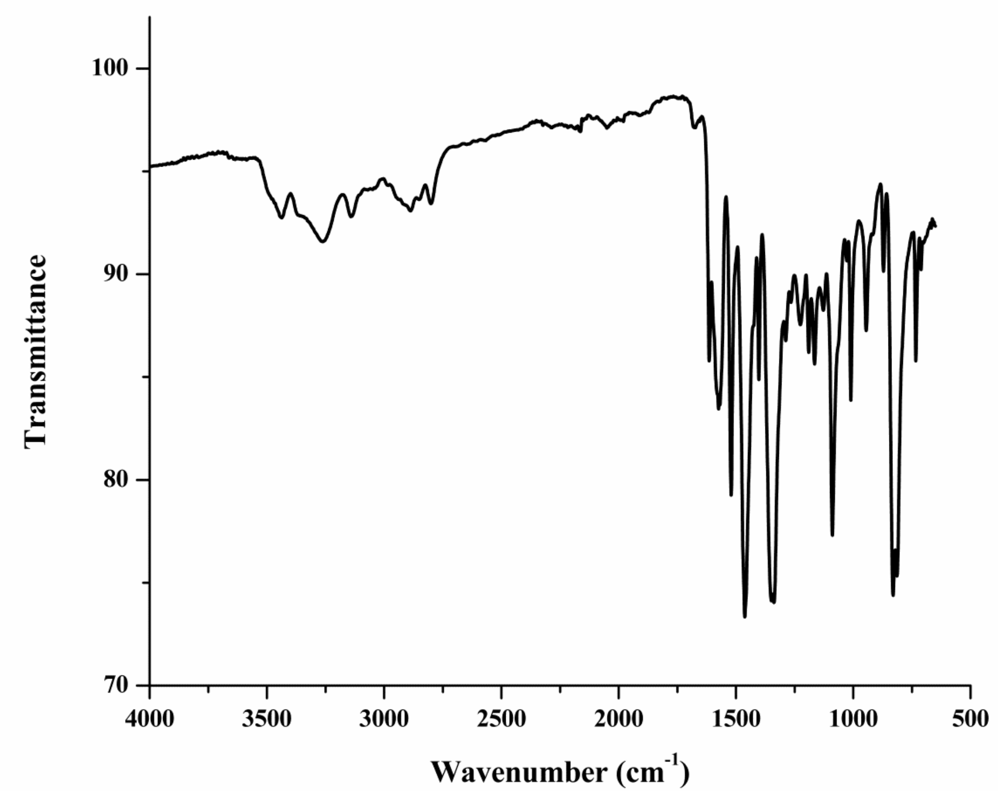
Transmittance
100
90
80
70
4000
3500
3000
2500
2000
1500
1000
500
Wavenumber (cm-1)

(3b)

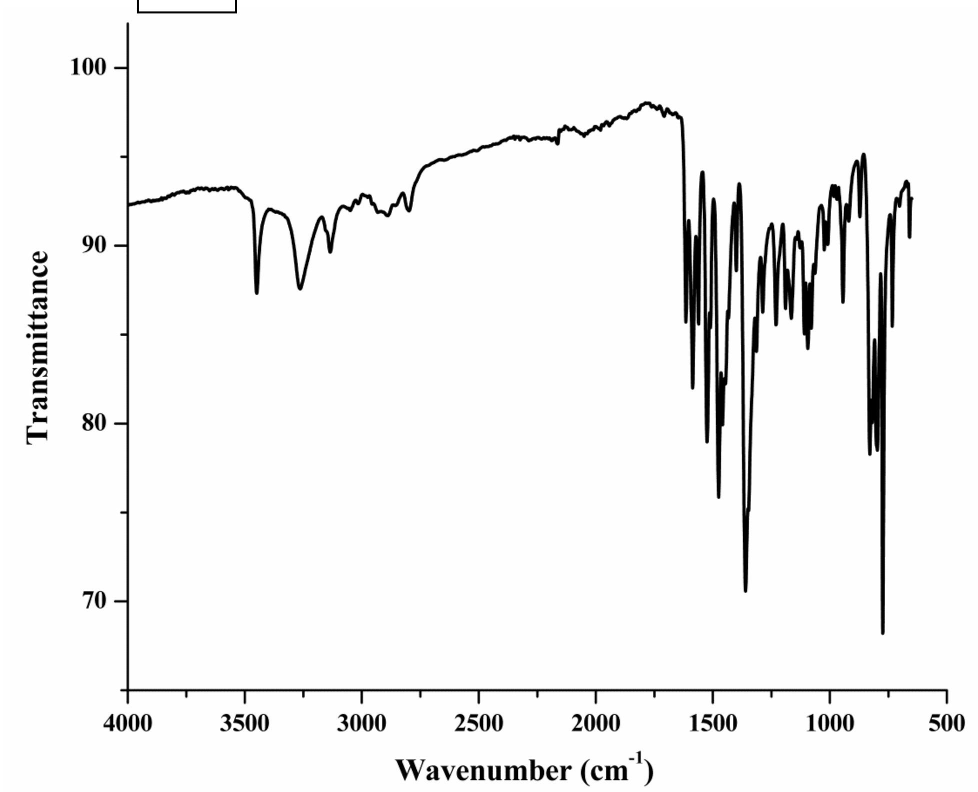
Transmittance
100
90
80
70
4000
3500
3000
2500
2000
1500
1000
500
Wavenumber (cm-1)

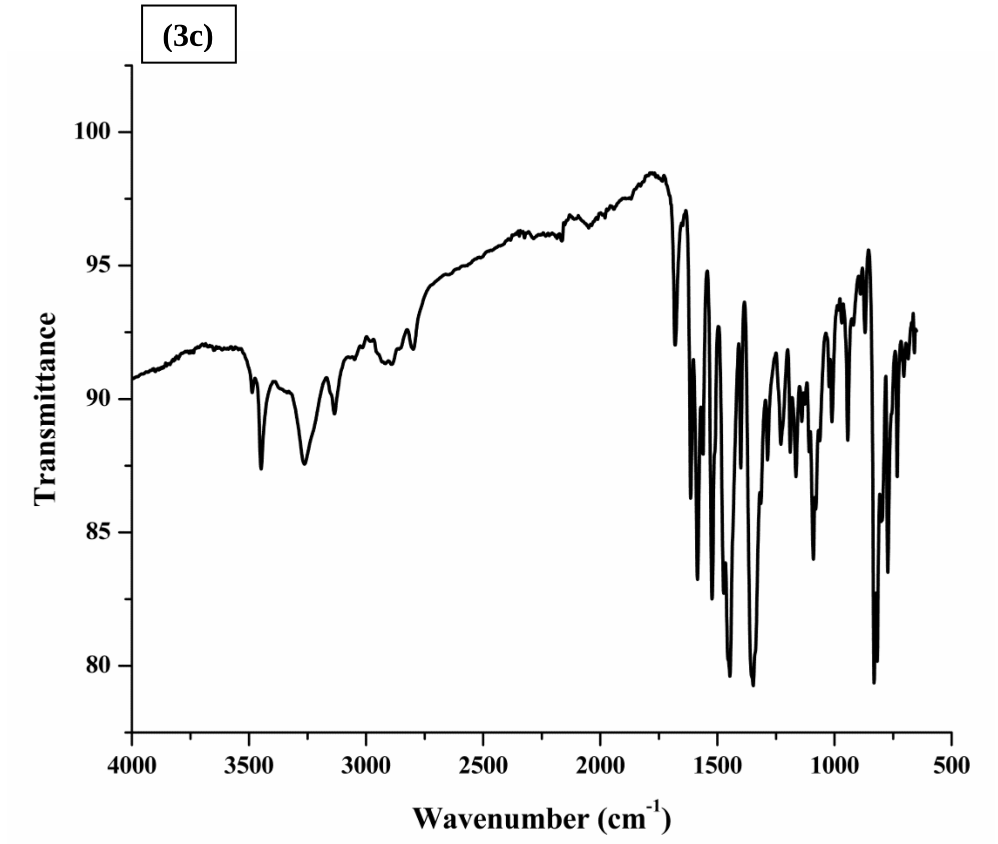
(3c)
Transmittance
100
95
90
85
80
4000
3500
3000
2500
2000
1500
1000
500
Wavenumber (cm-1)

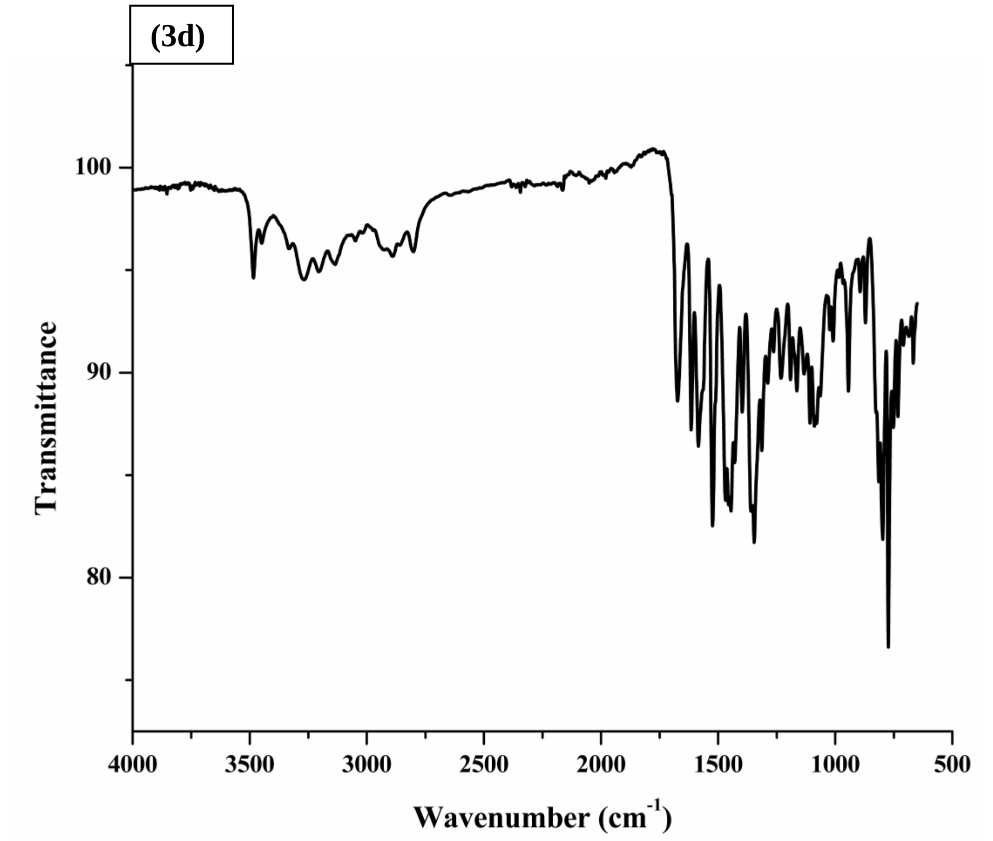
(3d)
Transmittance
100
90
80
4000
3500
3000
2500
2000
1500
1000
500
Wavenumber (cm-1)

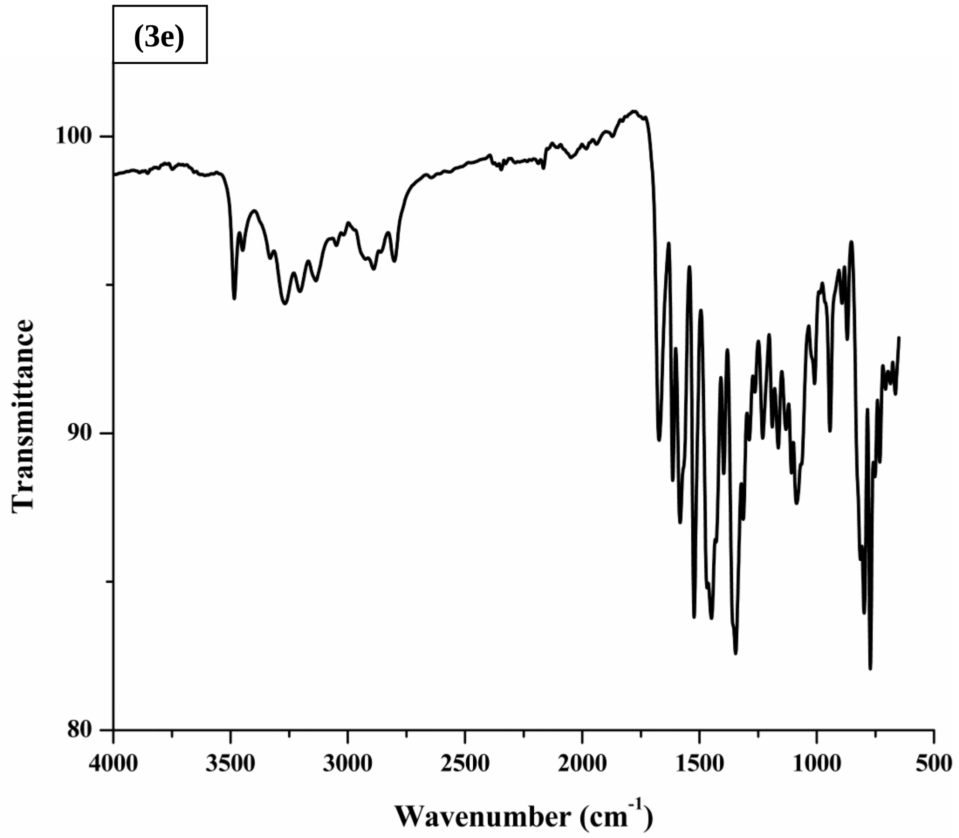
(3e)
100
90
80
Transmittance
4000
3500
3000
2500
2000
1500
1000
500
Wavenumber (cm-1)

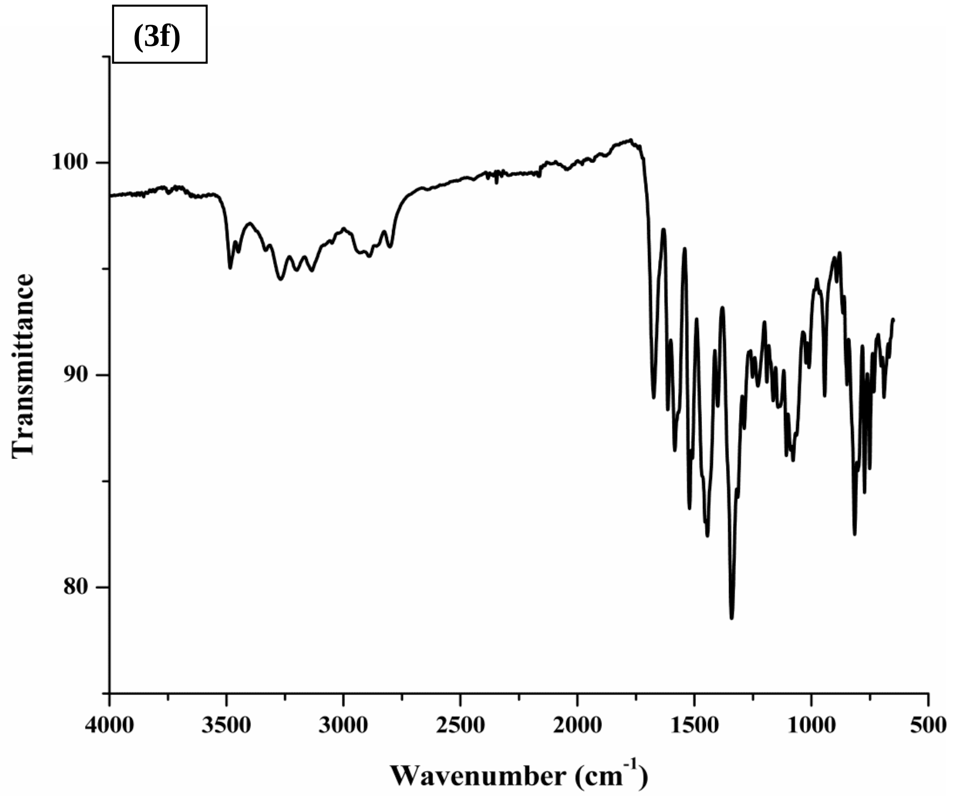
(3f)
100
90
80
Transmittance
4000
3500
3000
2500
2000
1500
1000
500
Wavenumber (cm-1)

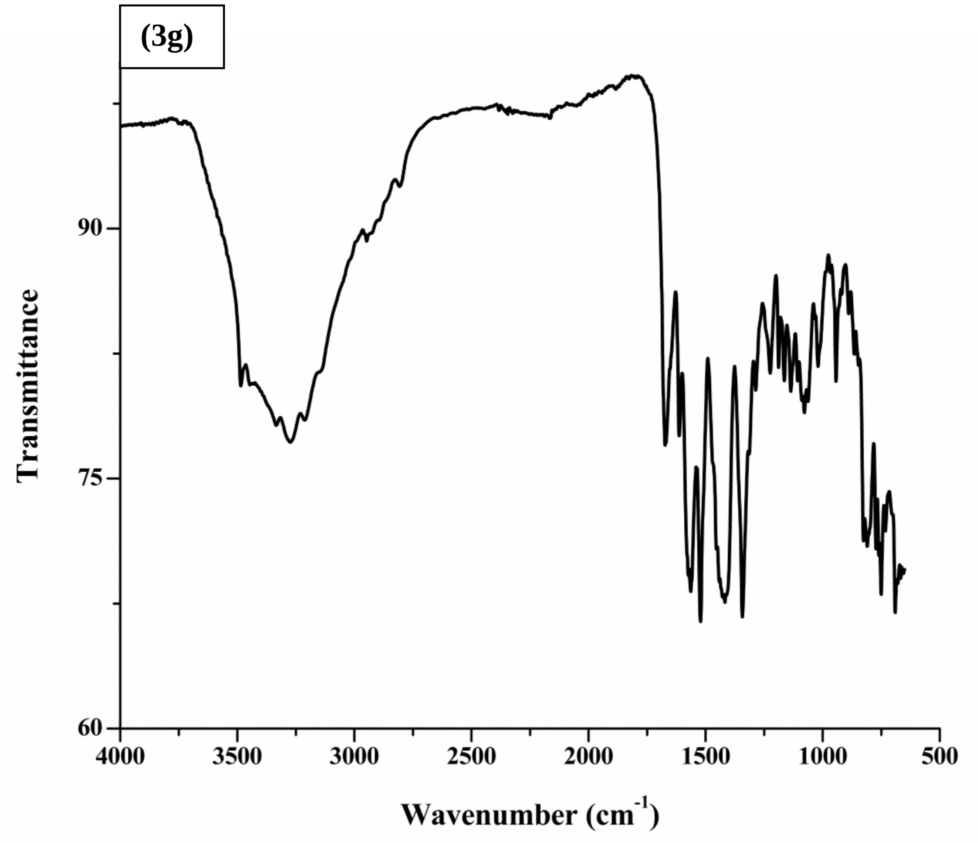
(3g)
Transmittance
90
75
60
4000
3500
3000
2500
2000
1500
1000
500
Wavenumber (cm-1)

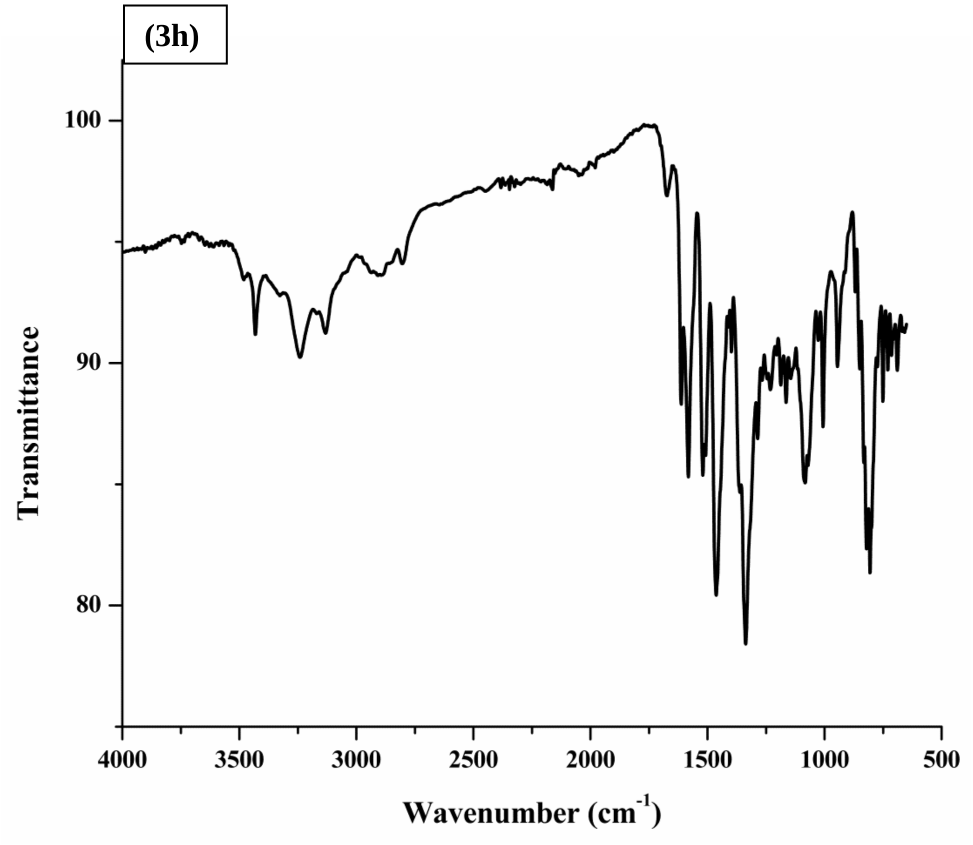
(3h)
Transmittance
100
90
80
4000
3500
3000
2500
2000
1500
1000
500
Wavenumber (cm-1)

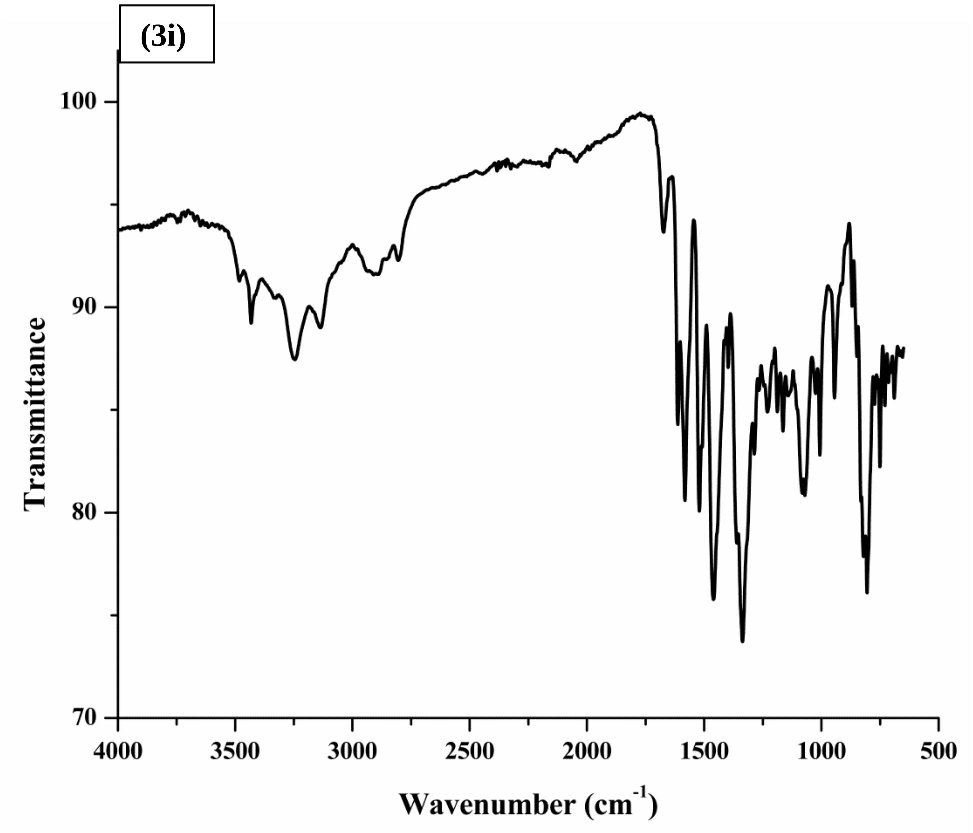
(3i)
Transmittance
100
90
80
70
4000
3500
3000
2500
2000
1500
1000
500
Wavenumber (cm-1)

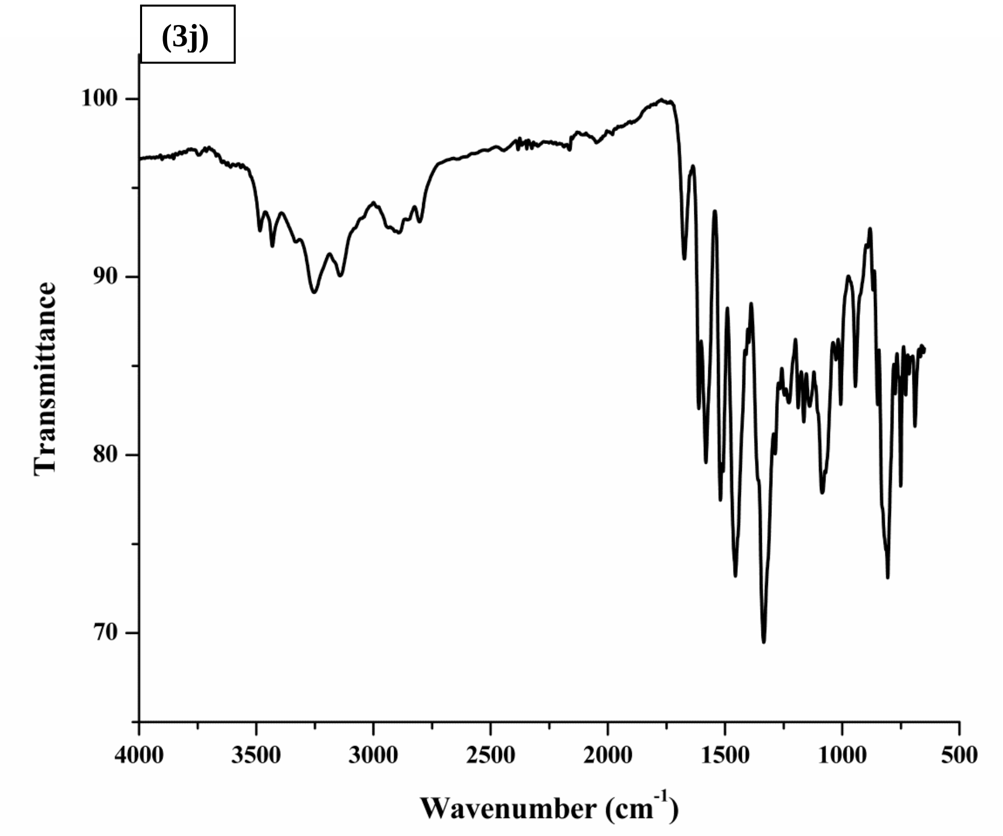
(3j)
Transmittance
100
90
80
70
4000
3500
3000
2500
2000
1500
1000
500
Wavenumber (cm-1)

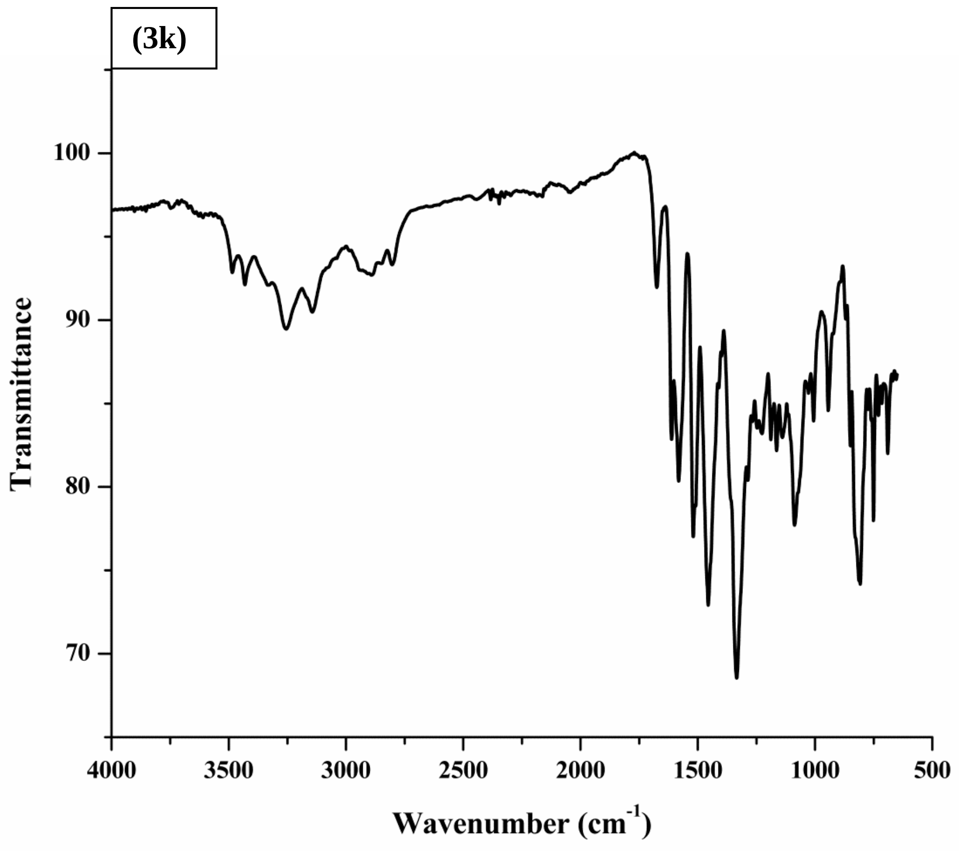
(3k)
Transmittance
100
90
80
70
4000
3500
3000
2500
2000
1500
1000
500
Wavenumber (cm-1)

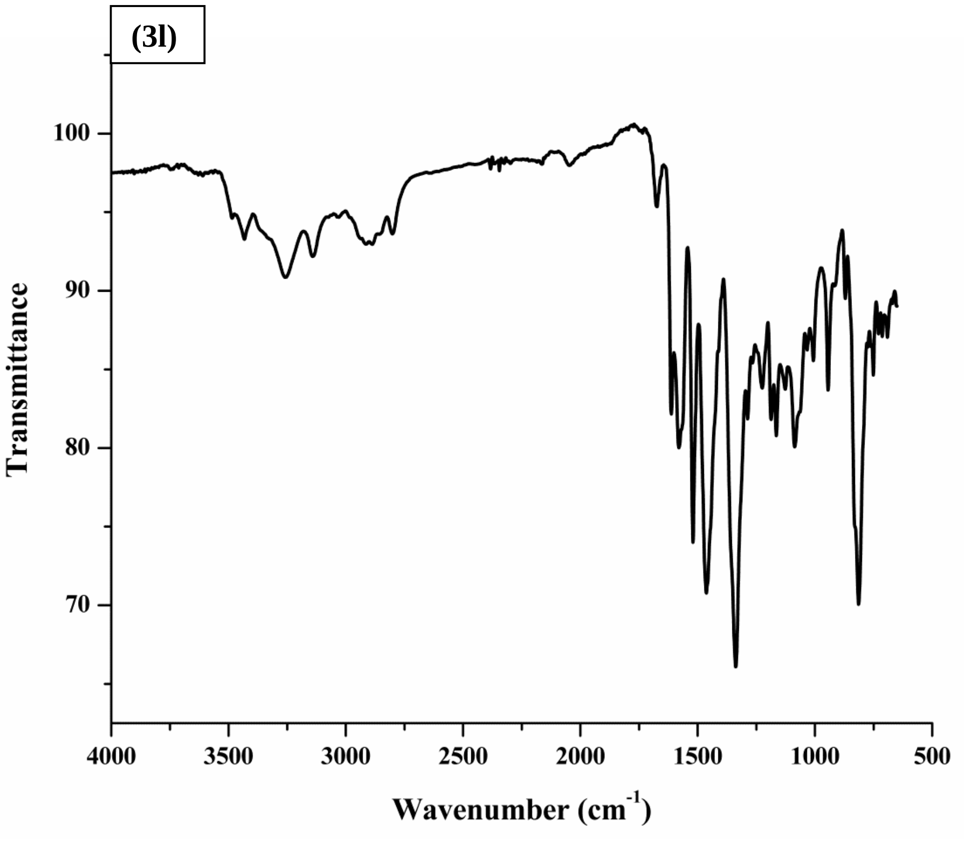
(3l)
Transmittance
100
90
80
70
4000
3500
3000
2500
2000
1500
1000
500
Wavenumber (cm-1)

Figure. S2. FTIR spectra of pyrazoline derivatives (3a-3l).

(3b)

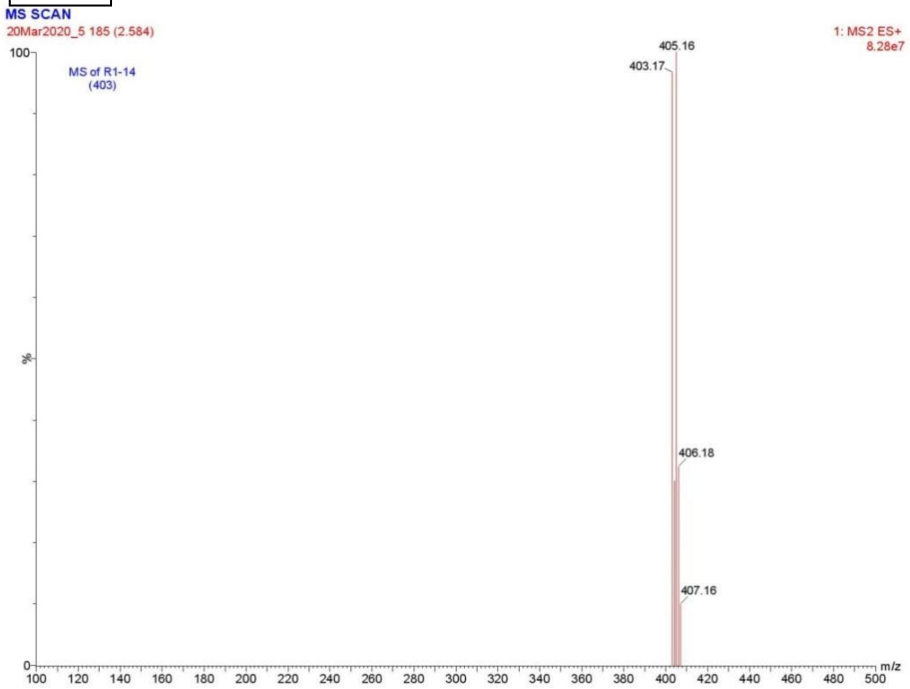

(3c)

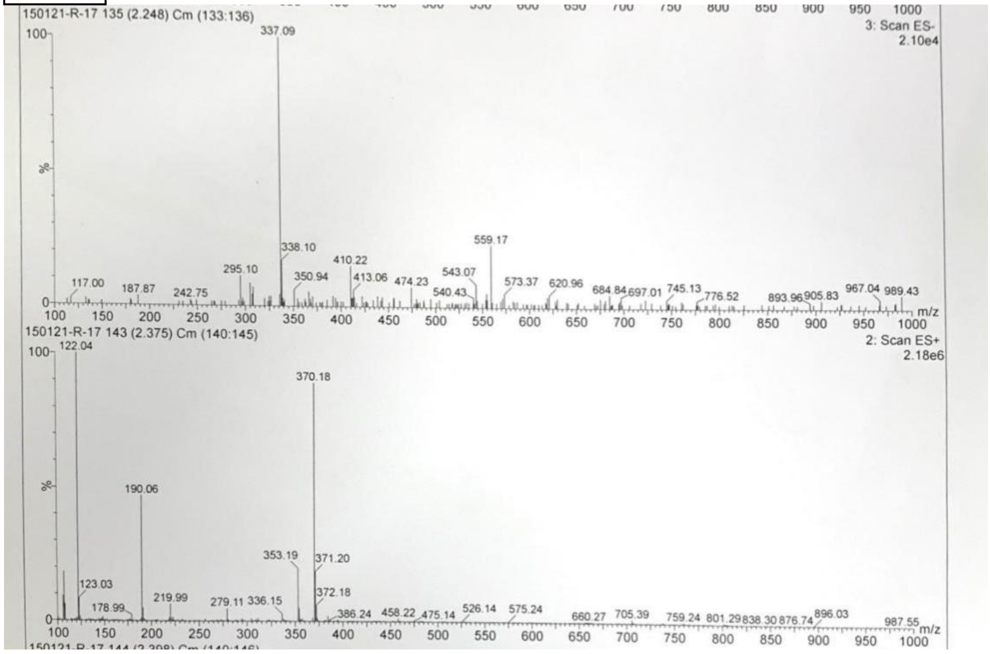

(3d)

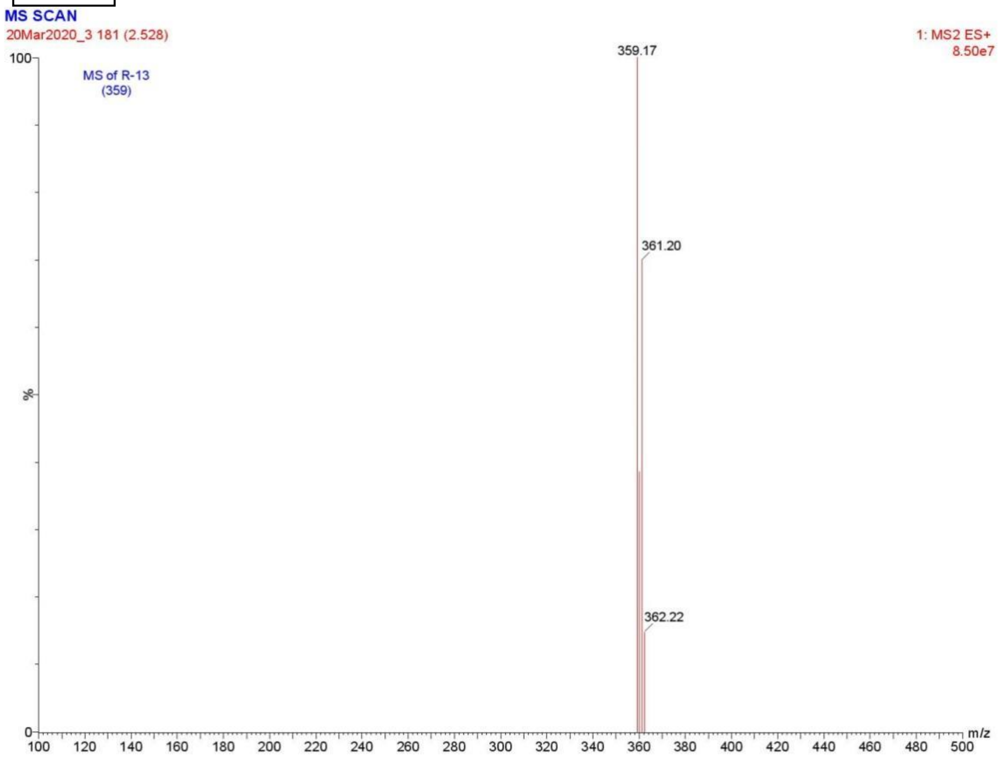
MS SCAN
20Mar2020_3 181 (2.528)
1: MS2 ES+
8.50e7
MS of R-13
(359)
359.17
361.20
362.22
%
m/z
100
0
100 120 140 160 180 200 220 240 260 280 300 320 340 360 380 400 420 440 460 480 500

(3e)

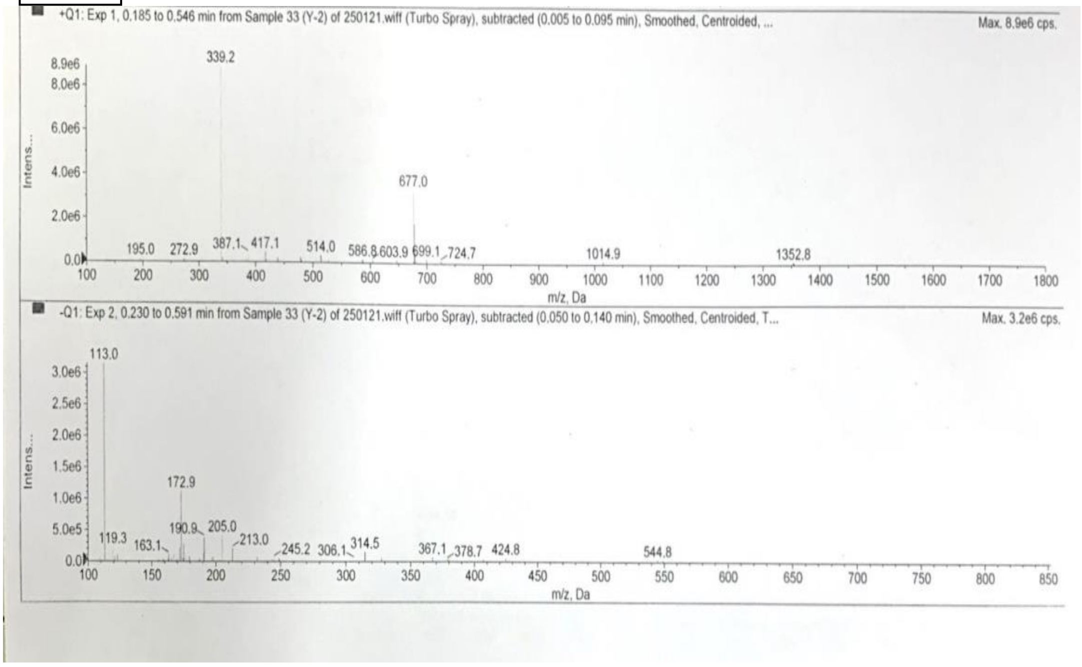
+Q1: Exp 1, 0.185 to 0.546 min from Sample 33 (Y-2) of 250121.wiff (Turbo Spray), subtracted (0.005 to 0.095 min), Smoothed, Centroided, ...
Max. 8.9e6 cps.
Intens...
339.2
677.0
195.0 272.9 387.1 417.1 514.0 586.8 603.9 699.1 724.7 1014.9 1352.8
m/z, Da
-Q1: Exp 2, 0.230 to 0.591 min from Sample 33 (Y-2) of 250121.wiff (Turbo Spray), subtracted (0.050 to 0.140 min), Smoothed, Centroided, T...
Max. 3.2e6 cps.
Intens...
113.0
172.9
119.3 163.1 190.9 205.0 213.0 245.2 306.1 314.5 367.1 378.7 424.8 544.8
m/z, Da

(3f)

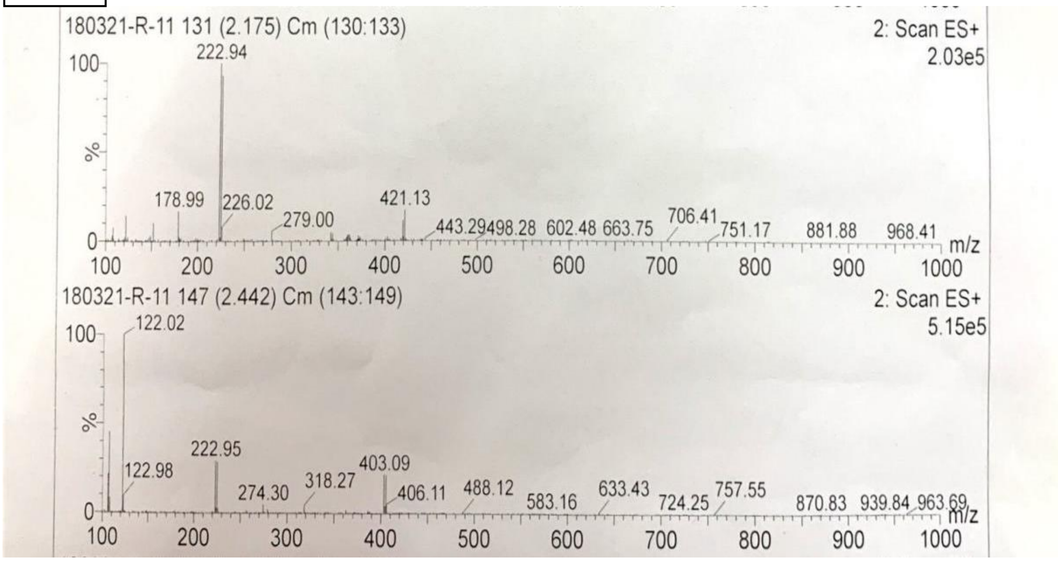
180321-R-11 131 (2.175) Cm (130:133)
2: Scan ES+
2.03e5
100
%
0
222.94
178.99
226.02
279.00
421.13
443.29
498.28
602.48
663.75
706.41
751.17
881.88
968.41
m/z
100 200 300 400 500 600 700 800 900 1000
180321-R-11 147 (2.442) Cm (143:149)
2: Scan ES+
5.15e5
122.02
122.98
222.95
274.30
318.27
403.09
406.11
488.12
583.16
633.43
724.25
757.55
870.83
939.84
963.69

(3g)

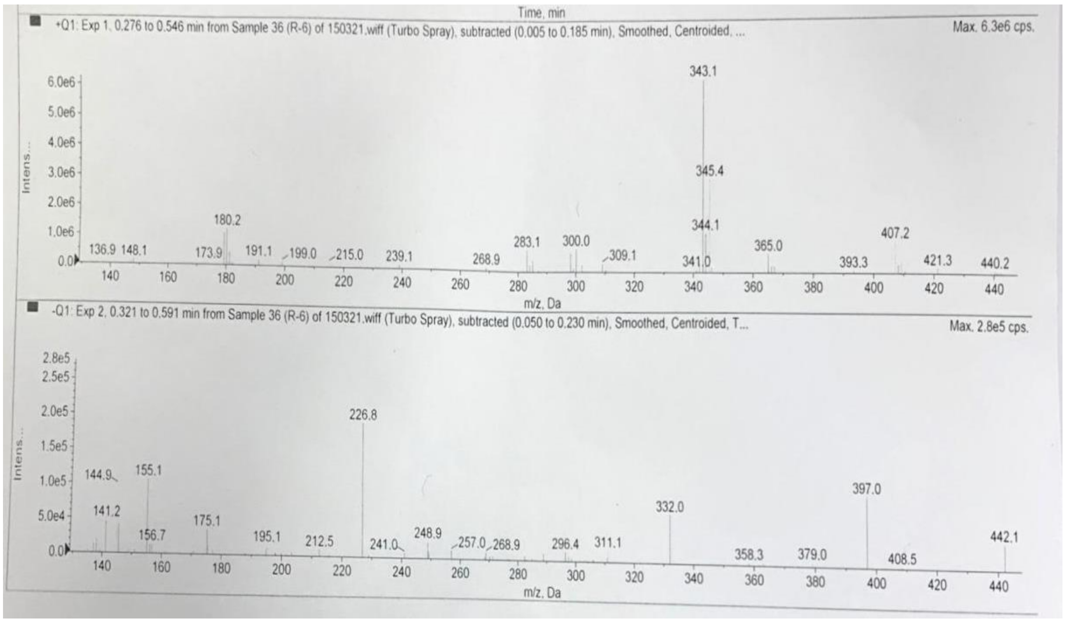
Time, min
+Q1: Exp 1, 0.276 to 0.546 min from Sample 36 (R-6) of 150321.wiff (Turbo Spray), subtracted (0.005 to 0.185 min), Smoothed, Centroided, ...
Max. 6.3e6 cps.
Intens...
343.1
345.4
344.1
180.2
407.2
365.0
283.1
300.0
309.1
341.0
393.3
421.3
440.2
136.9
148.1
173.9
191.1
199.0
215.0
239.1
268.9
m/z, Da
-Q1: Exp 2, 0.321 to 0.591 min from Sample 36 (R-6) of 150321.wiff (Turbo Spray), subtracted (0.050 to 0.230 min), Smoothed, Centroided, T...
Max. 2.8e5 cps.
226.8
144.9
155.1
141.2
156.7
175.1
195.1
212.5
241.0
248.9
257.0
268.9
296.4
311.1
332.0
358.3
379.0
397.0
408.5
442.1
m/z, Da

(3i)

Display Report

Analysis Info

Analysis Name	D:\Data\March 2020\m chem aks-nsh-299_RB7_01_1309.d	Acquisition Date	3/19/2020 6:04:49 PM
Method	2. LCMS tune wide ACN.m	Operator	IIT Indore
Sample Name	m chem aks-nsh-299	Instrument	micrOTOF-Q 228888.10348
Comment			

Acquisition Parameter

Source Type	ESI	Ion Polarity	Positive	Set Nebulizer	2.0 Bar
Focus	Not active	Set Capillary	4500 V	Set Dry Heater	250 °C
Scan Begin	50 m/z	Set End Plate Offset	-500 V	Set Dry Gas	7.0 l/min
Scan End	3000 m/z	Set Collision Cell RF	650.0 Vpp	Set Divert Valve	Waste

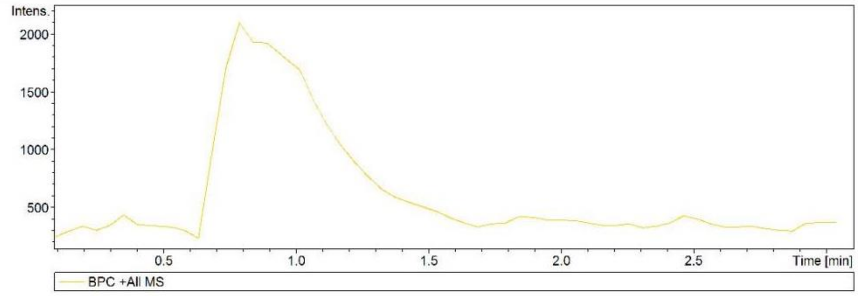

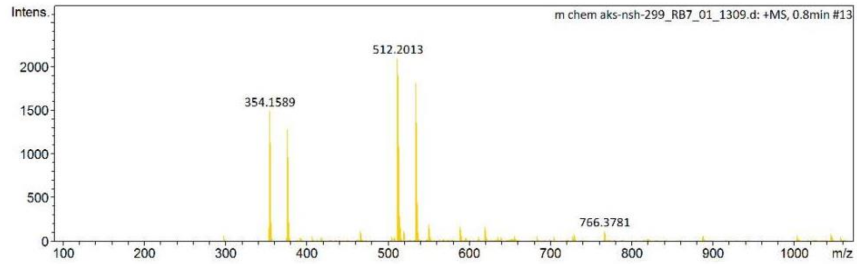

(3j)

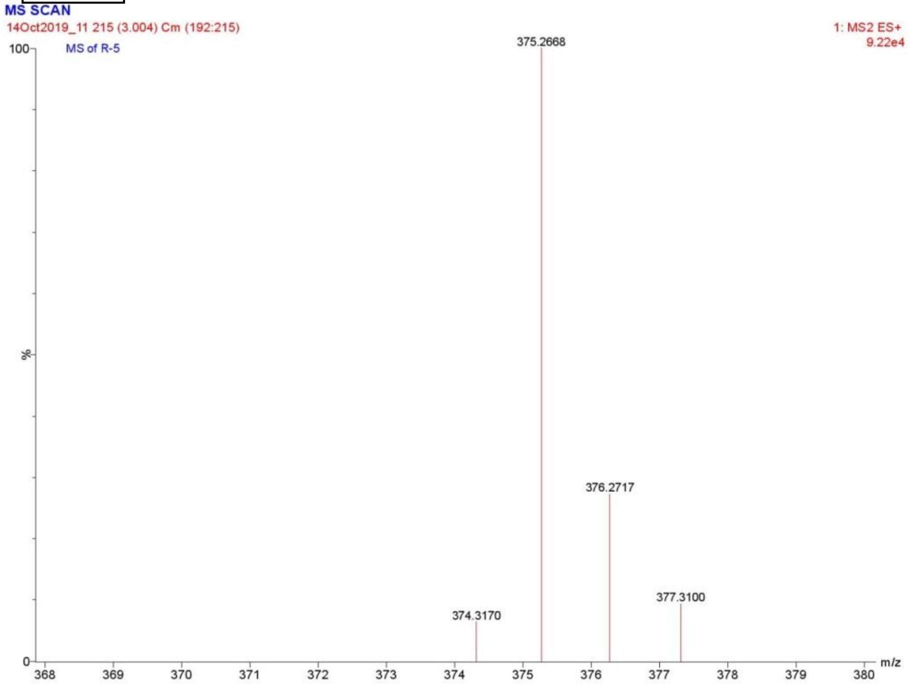

(3k)

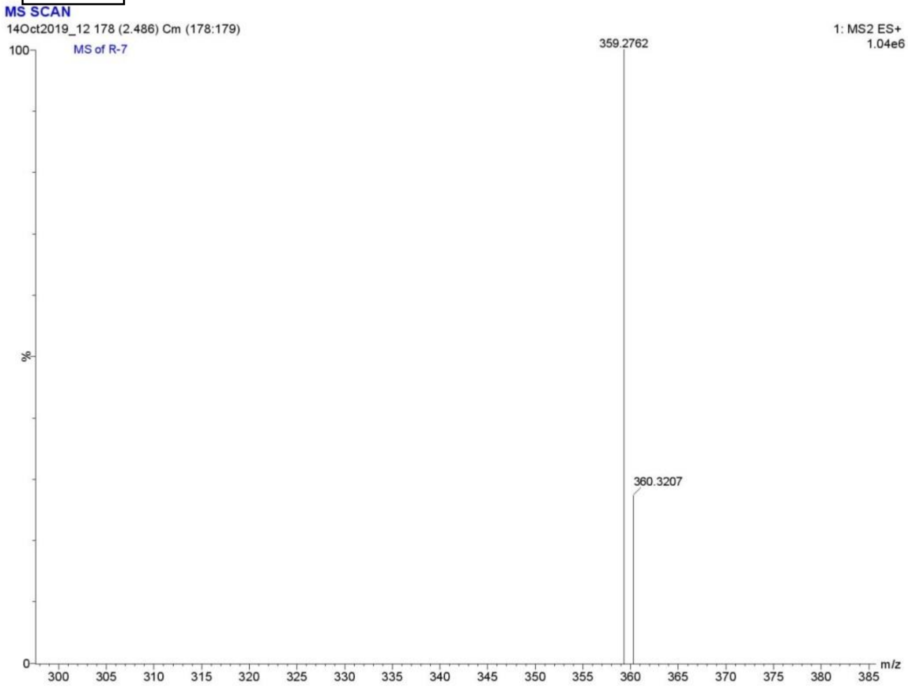
MS SCAN
14Oct2019_12 178 (2.486) Cm (178:179)
1: MS2 ES+
1.04e6
MS of R-7
359.2762
360.3207
100
%
0
m/z
300 305 310 315 320 325 330 335 340 345 350 355 360 365 370 375 380 385

(3l)

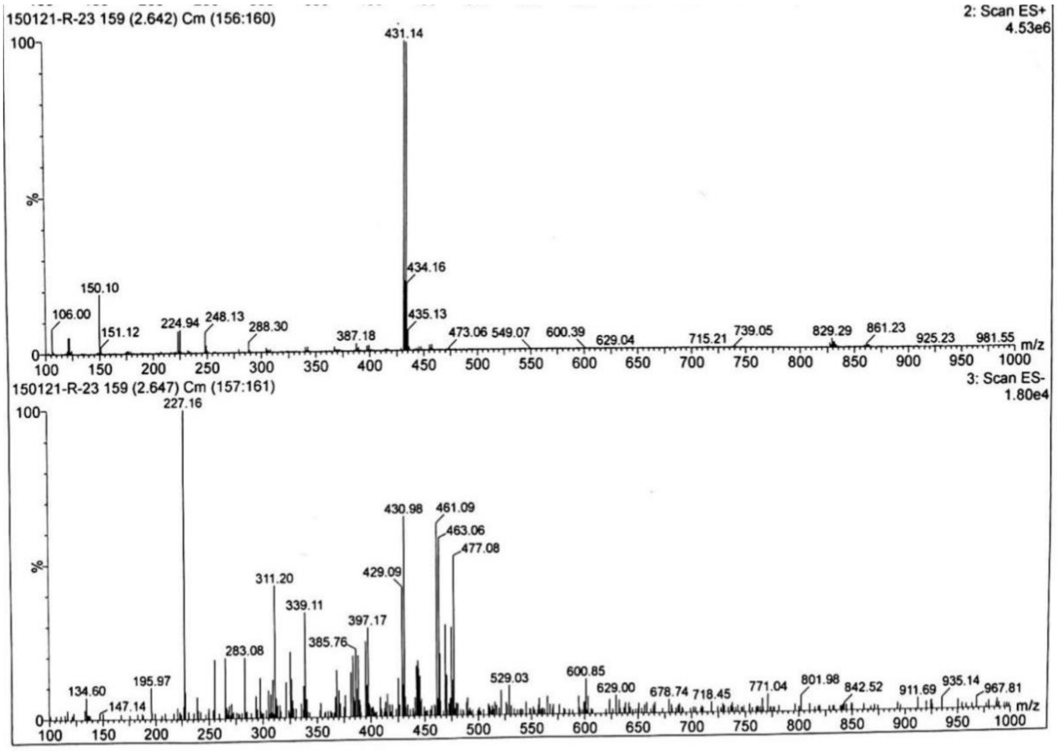
150121-R-23 159 (2.642) Cm (156:160)
2: Scan ES+
4.53e6
431.14
434.16
435.13
150.10
106.00
151.12
224.94
248.13
288.30
387.18
473.06
549.07
600.39
629.04
715.21
739.05
829.29
861.23
925.23
981.55
150121-R-23 159 (2.647) Cm (157:161)
3: Scan ES-
1.80e4
227.16
430.98
461.09
463.06
477.08
429.09
311.20
339.11
397.17
385.76
283.08
134.60
147.14
195.97
529.03
600.85
629.00
678.74
718.45
771.04
801.98
842.52
911.69
935.14
967.81
100
%
0
m/z
100 150 200 250 300 350 400 450 500 550 600 650 700 750 800 850 900 950 1000

Figure S3. Mass spectra of pyrazoline derivatives (3a-3l).

3a

SAMPLE INFORMATION

Sample Name :	A-3-XBS-ABC	Acquired By :	System HPLC_01
Vial :	20	Acq. Method Set :	NON POLAR C@A20MIN 40%
Injection # :	1	Injection Volume :	3.00 ul
Run Time :	20.0 Minutes	Proc. Chnl. Descr.:	2998 PDA 214.0 nm (2998
Date Acquired:	24-02-2022 19:32:26 IST	Date Processed :	24-02-2022 20:01:56 IST

Column Name : XSELECT PHENYL HEXYL (4.6 X 250 mm)5 µ
Mobile phase : C: 5mM ABC in water A : 100% ACN
Flow rate : 1 ml/min, Temperature : Ambient
Flow mode : Gradient , Time/C (%) : 0.0/50.0, 2.0/50.0, 10.0/20.0, 15/05.0, 17.0/05.0, 17.50/50.0. 20.0/50.0

Auto-Scaled Chromatogram

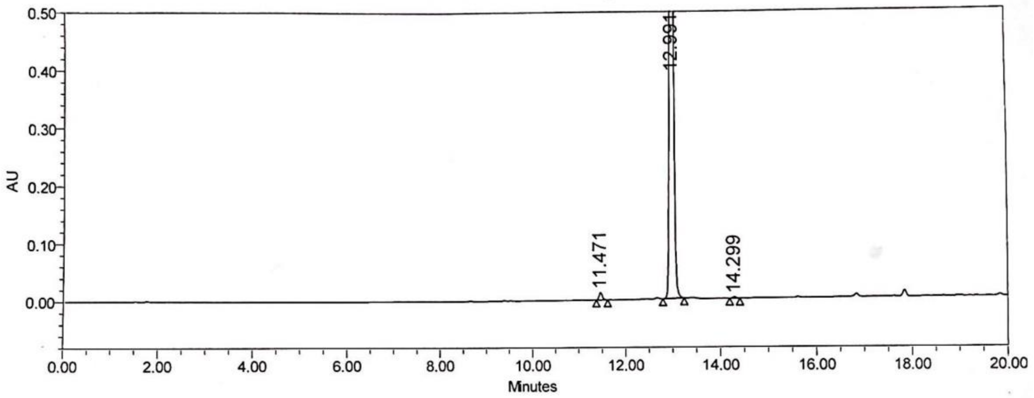

Peak Results

	RT	Area	% Area
1	11.471	62122	0.91
2	12.991	6780194	98.91
3	14.299	12733	0.19

3b

SAMPLE INFORMATION

Sample Name :	B-3-XBS-ABC	Acquired By :	System HPLC_01
Vial :	71	Acq. Method Set :	NON POLAR C@A20MIN 40%
Injection # :	1	Injection Volume :	3.00 ul
Run Time :	20.0 Minutes	Proc. Chnl. Descr.:	2998 PDA 328.0 nm (2998
Date Acquired:	25-02-2022 16:46:00 IST	Date Processed :	25-02-2022 18:23:30 IST

Column Name : XSELECT PHENYL HEXYL (4.6 X 250 mm)5 μ
Mobile phase : C: 5mM ABC in water A : 100% ACN
Flow rate : 1 ml/min, Temperature : Ambient
Flow mode : Gradient , Time/C (%) : 0.0/50.0, 2.0/50.0, 10.0/20.0, 15/05.0, 17.0/05.0, 17.50/50.0. 20.0/50.0

Auto-Scaled Chromatogram

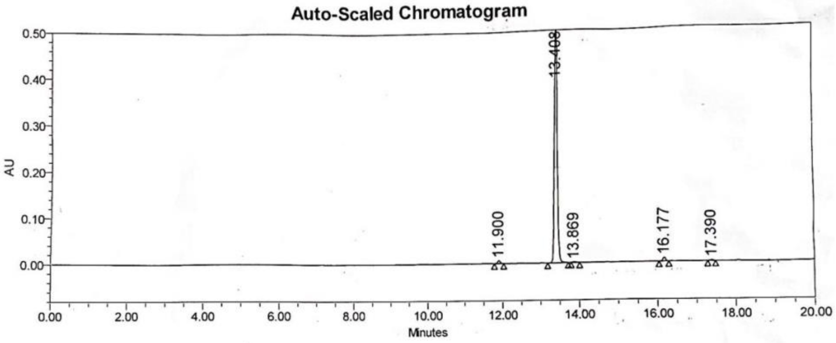

Peak Results

	RT	Area	% Area
1	11.900	32052	1.20
2	13.408	2589429	96.83
3	13.869	7716	0.29
4	16.177	38247	1.43
5	17.390	6715	0.25

3c

SAMPLE INFORMATION

Sample Name :	C-3-XBS-ABC	Acquired By :	System HPLC_01
Vial :	104	Acq. Method Set :	NON POLAR C@A20MIN 40%
Injection # :	1	Injection Volume :	3.00 ul
Run Time :	20.0 Minutes	Proc. Chnl. Descr.:	2998 PDA 254.0 nm (2998
Date Acquired:	24-02-2022 18:46:40 IST	Date Processed :	24-02-2022 19:16:50 IST

Column Name : XSELECT PHENYL HEXYL (4.6 X 250 mm)5 μ
Mobile phase : C: 5mM ABC in water A : 100% ACN
Flow rate : 1 ml/min, Temperature : Ambient
Flow mode : Gradient , Time/C (%) : 0.0/50.0, 2.0/50.0, 10.0/20.0, 15/05.0, 17.0/05.0, 17.50/50.0. 20.0/50.0

Auto-Scaled Chromatogram

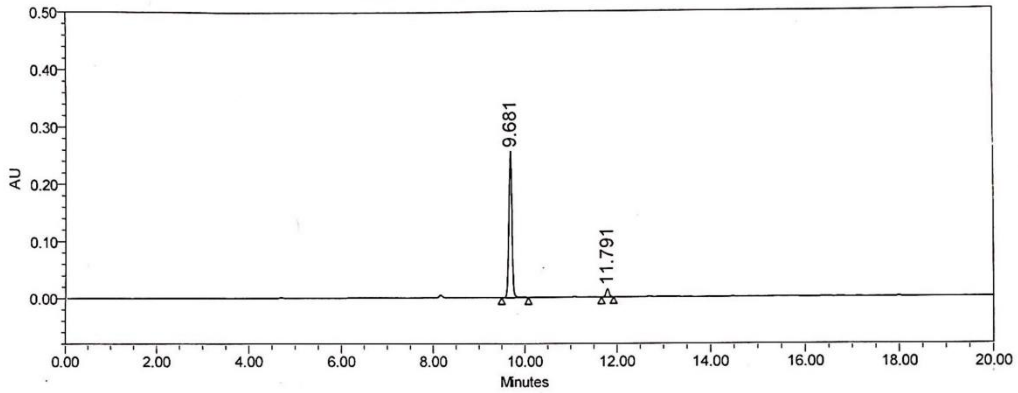

Peak Results

	RT	Area	% Area
1	9.681	1203403	94.65
2	11.791	68016	5.35

3d

SAMPLE INFORMATION

Sample Name :	D-3-XBS-ABC	Acquired By :	System HPLC_01
Vial :	72	Acq. Method Set :	NON POLAR C@A20MIN 40%
Injection # :	1	Injection Volume :	3.00 ul
Run Time :	20.0 Minutes	Proc. Chnl. Descr.:	2998 PDA 214.0 nm (2998
Date Acquired:	25-02-2022 17:08:46 IST	Date Processed :	25-02-2022 18:21:10 IST

Column Name : XSELECT PHENYL HEXYL (4.6 X 250 mm)5 μ
Mobile phase : C: 5mM ABC in water A : 100% ACN
Flow rate : 1 ml/min, Temperature : Ambient
Flow mode : Gradient , Time/C (%) : 0.0/50.0, 2.0/50.0, 10.0/20.0, 15/05.0, 17.0/05.0, 17.50/50.0. 20.0/50.0

Auto-Scaled Chromatogram

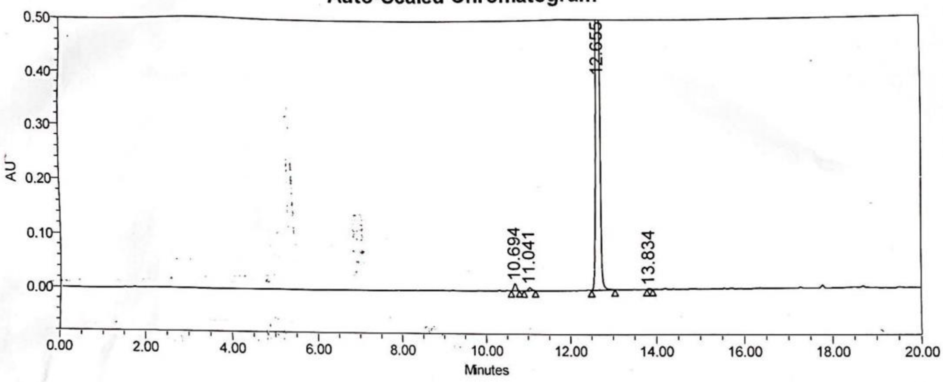

Peak Results

	RT	Area	% Area
1	10.694	65244	1.11
2	11.041	32983	0.56
3	12.655	5782277	98.21
4	13.834	6952	0.12

3e

SAMPLE INFORMATION

Name :	E-3-XBS-ABC	Acquired By :	System HPLC_01
Vial :	49	Acq. Method Set :	NON POLAR C@A20MIN 40%
Injection # :	1	Injection Volume :	3.00 ul
Run Time :	20.0 Minutes	Proc. Chnl. Descr.:	2998 PDA 260.0 nm (2998
Date Acquired:	25-02-2022 17:31:29 IST	Date Processed :	25-02-2022 18:19:07 IST

Column Name : XSELECT PHENYL HEXYL (4.6 X 250 mm)5 μ
Mobile phase : C: 5mM ABC in water A : 100% ACN
Flow rate : 1 ml/min, Temperature : Ambient
Flow mode : Gradient , Time/C (%) : 0.0/50.0, 2.0/50.0, 10.0/20.0, 15/05.0, 17.0/05.0, 17.50/50.0. 20.0/50.0

Auto-Scaled Chromatogram

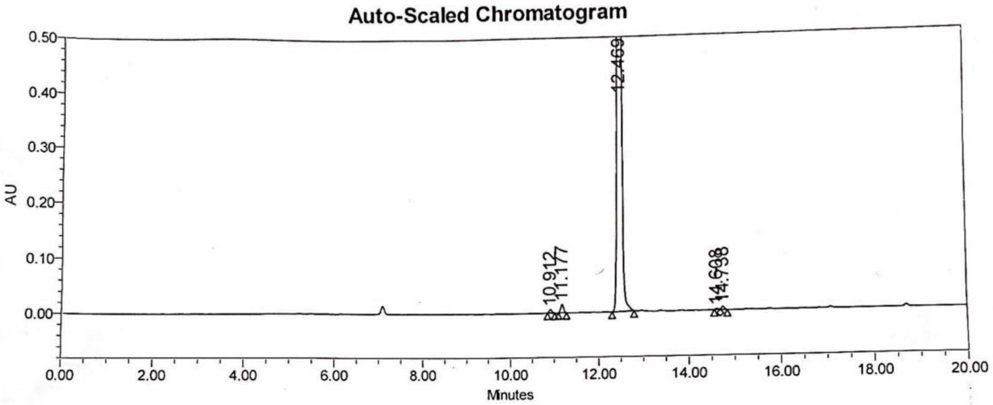

Peak Results

	RT	Area	% Area
1	10.912	28696	0.29
2	11.177	70449	0.72
3	12.469	9692199	98.61
4	14.608	11032	0.11
5	14.738	26581	0.27

3f

SAMPLE INFORMATION

Sample Name :	S3-XBS-ABC	Acquired By :	System HPLC_01
Vial :	103	Acq. Method Set :	NON POLAR C@A20MIN 40%
Injection # :	1	Injection Volume :	3.00 ul
Run Time :	20.0 Minutes	Proc. Chnl. Descr.:	2998 PDA 258.0 nm (2998
Date Acquired:	23-02-2022 19:17:27 IST	Date Processed :	23-02-2022 19:40:37 IST

Column Name : XSELECT PHENYL HEXYL (4.6 X 250 mm)5 μ
Mobile phase : C: 5mM ABC in water A : 100% ACN
Flow rate : 1 ml/min, Temperature : Ambient
Flow mode : Gradient , Time/C (%) : 0.0/50.0, 2.0/50.0, 10.0/20.0, 15/05.0, 17.0/05.0, 17.50/50.0. 20.0/50.0

Auto-Scaled Chromatogram

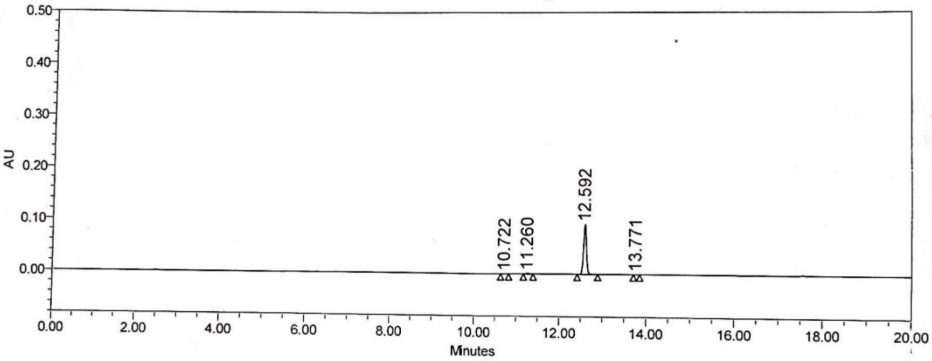

Peak Results

	RT	Area	% Area
1	10.722	2024	0.44
2	11.260	7475	1.63
3	12.592	447879	97.72
4	13.771	930	0.20

3g

SAMPLE INFORMATION

Sample Name :	G-3-XBS-ABC	Acquired By :	System HPLC_01
Vial :	106	Acq. Method Set :	NON POLAR C@A20MIN 40%
Injection # :	1	Injection Volume :	3.00 ul
Run Time :	20.0 Minutes	Proc. Chnl. Descr.:	2998 PDA 214.0 nm (2998
Date Acquired:	24-02-2022 19:55:28 IST	Date Processed :	24-02-2022 20:17:13 IST

Column Name : XSELECT PHENYL HEXYL (4.6 X 250 mm)5 μ
Mobile phase : C: 5mM ABC in water A : 100% ACN
Flow rate : 1 ml/min, Temperature : Ambient
Flow mode : Gradient , Time/C (%) : 0.0/50.0, 2.0/50.0, 10.0/20.0, 15/05.0, 17.0/05.0, 17.50/50.0. 20.0/50.0

Auto-Scaled Chromatogram

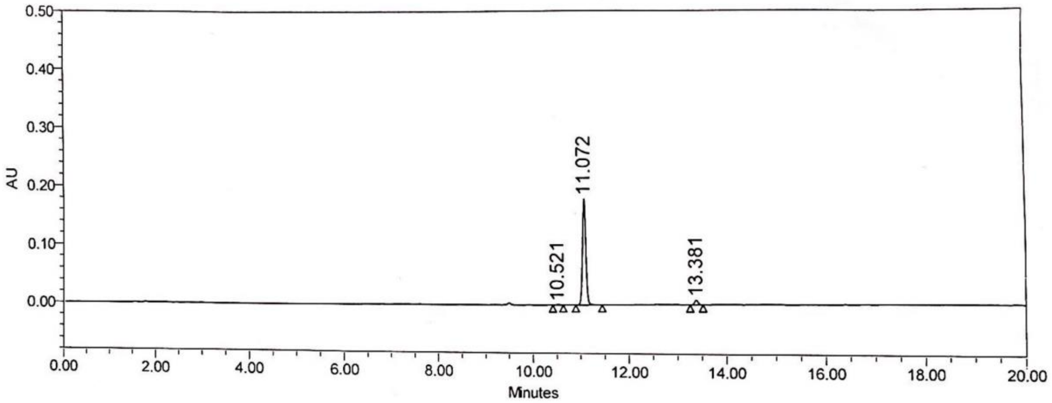

Peak Results

	RT	Area	% Area
1	10.521	5061	0.55
2	11.072	870230	95.00
3	13.381	40719	4.45

3h

SAMPLE INFORMATION

Sample Name :	H3-XBS-ABC	Acquired By :	System HPLC_01
Vial :	23	Acq. Method Set :	NON POLAR C@A20MIN 40%
Injection # :	1	Injection Volume :	5.00 ul
Run Time :	20.0 Minutes	Proc. Chnl. Descr.:	2998 PDA 214.0 nm (2998
Date Acquired:	02-03-2022 19:01:40 IST	Date Processed :	02-03-2022 19:25:19 IST

Column Name : XSELECT PHENYL HEXYL (4.6 X 250 mm)5 µ
Mobile phase : C: 5mM ABC in water A : 100% ACN
Flow rate : 1 ml/min, Temperature : Ambient
Flow mode : Gradient , Time/C (%) : 0.0/50.0, 2.0/50.0, 10.0/20.0, 15/05.0, 17.0/05.0, 17.50/50.0. 20.0/50.0

Auto-Scaled Chromatogram

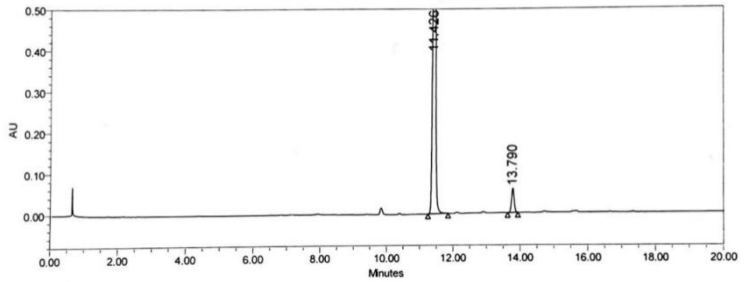

Peak Results

	RT	Area	% Area
1	11.426	7042027	95.57
2	13.790	326588	4.43

3i

SAMPLE INFORMATION

Sample Name :	I3	Acquired By :	System HPLC_01
Vial :	57	Acq. Method Set :	NON POLAR C@A20MIN 40%
Injection # :	1	Injection Volume :	3.00 ul
Run Time :	20.0 Minutes	Proc. Chnl. Descr.:	2998 PDA 270.0 nm (2998 (190-400)nm)
Date Acquired:	18-02-2022 17:55:39 IST	Date Processed :	18-02-2022 18:31:07 IST

Column Name : XBridgeC8 (4.6 X 250 mm)5 µ
Mobile phase : C : 0.1% AH in Water A : 100% ACN
Flow rate : 1 ml/min, Column Temperature : Ambient.
Flow mode : Gradient , Time/C (%) :: 0.0/60.0, 2.0/60.0, 9.0/25.0, 15.0/5.0, 18.0/5.0, 18.50/60.0, 20.0/60.0

Auto-Scaled Chromatogram

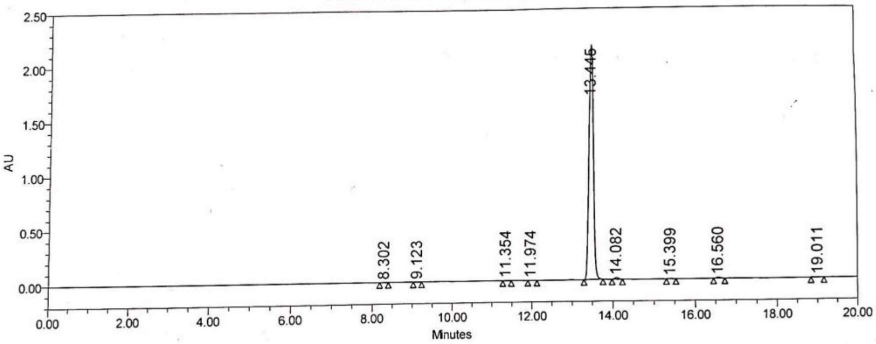

Peak Results

	RT	Area	% Area
1	8.302	11162	0.07
2	9.123	8228	0.05
3	11.354	7115	0.04
4	11.974	7084	0.04
5	13.445	16191623	98.51
6	14.082	107155	0.65
7	15.399	26533	0.16
8	16.560	66586	0.41
9	19.011	11701	0.07

3j

SAMPLE INFORMATION

Sample Name :	J3-XBS-ABC	Acquired By :	System HPLC_01
Vial :	101	Acq. Method Set :	NON POLAR C@A20MIN 40%
Injection # :	1	Injection Volume :	3.00 ul
Run Time :	20.0 Minutes	Proc. Chnl. Descr.:	2998 PDA 214.0 nm (2998
Date Acquired:	23-02-2022 18:32:06 IST	Date Processed :	23-02-2022 18:56:48 IST

Column Name : XSELECT PHENYL HEXYL (4.6 X 250 mm)5 µ
Mobile phase : C: 5mM ABC in water A : 100% ACN
Flow rate : 1 ml/min, Temperature : Ambient
Flow mode : Gradient , Time/C (%) : 0.0/50.0, 2.0/50.0, 10.0/20.0, 15/05.0, 17.0/05.0, 17.50/50.0. 20.0/50.0

Auto-Scaled Chromatogram

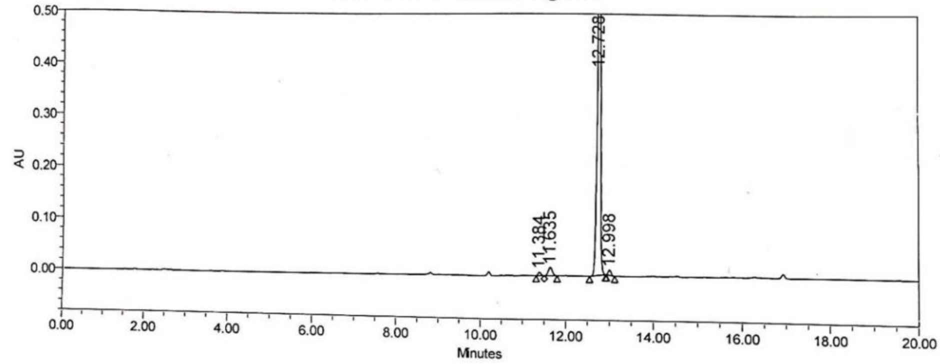

Peak Results

	RT	Area	% Area
1	11.384	29812	0.75
2	11.635	101812	2.55
3	12.728	3822752	95.61
4	12.998	43917	1.10

3k

SAMPLE INFORMATION

Sample Name :	K-3-XBS-ABC	Acquired By :	System HPLC_01
Vial :	96	Acq. Method Set :	NON POLAR C@A20MIN 40%
Injection # :	1	Injection Volume :	3.00 ul
Run Time :	20.0 Minutes	Proc. Chnl. Descr.:	2998 PDA 214.0 nm (2998 (190-400)nm)
Date Acquired:	07-03-2022 17:59:11 IST	Date Processed :	07-03-2022 18:49:07 IST

Column Name : XBS(4.6 X 250 mm)5 µ
Mobile phase : C : 5mM ABC in water A : 100% ACN
Flow rate : 1 ml/min, Temperature : Ambient
Flow mode : Gradient , Time/C (%) : 0.0/70.0, 2.0/70.0,6.0/40.0, 12.0/40.0, 15.0/5.0, 18.0/5.0, 18.50/70.0,20.0/70.0

Auto-Scaled Chromatogram

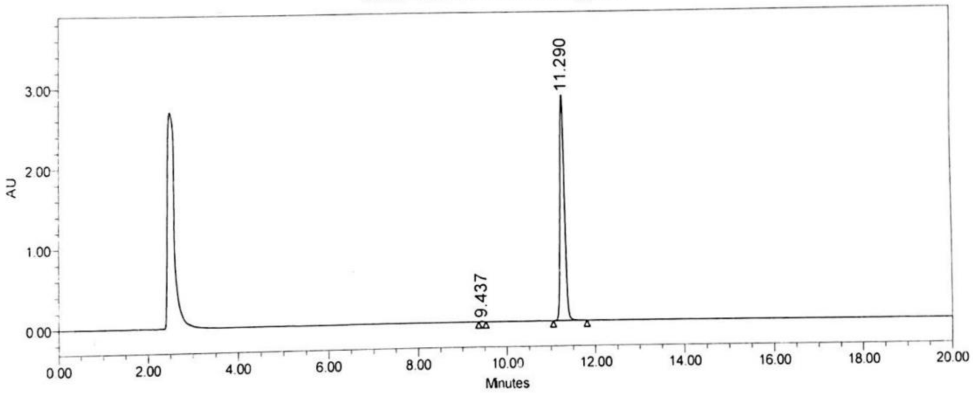

Peak Results

	RT	Area	% Area
1	9.437	42547	0.21
2	11.290	19795397	99.79

3l

SAMPLE INFORMATION

Sample Name :	l3	Acquired By :	System HPLC_01
Vial :	57	Acq. Method Set :	NON POLAR C@A20MIN 40%
Injection # :	1	Injection Volume :	3.00 ul
Run Time :	20.0 Minutes	Proc. Chnl. Descr.:	2998 PDA 270.0 nm (2998 (190-400)nm)
Date Acquired:	18-02-2022 17:55:39 IST	Date Processed :	18-02-2022 18:31:07 IST

Column Name : XBridgeC8 (4.6 X 250 mm)5 µ
Mobile phase : C : 0.1% AH in Water A : 100% ACN
Flow rate : 1 ml/min, Column Temperature : Ambient.
Flow mode : Gradient , Time/C (%) :: 0.0/60.0, 2.0/60.0, 9.0/25.0, 15.0/5.0, 18.0/5.0, 18.50/60.0, 20.0/60.0

Auto-Scaled Chromatogram

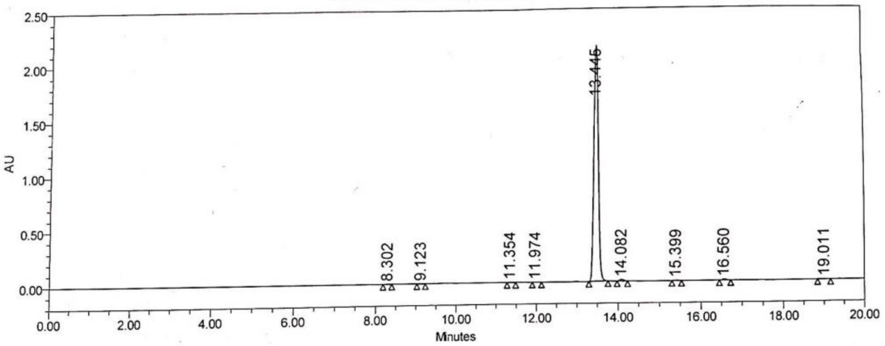

Peak Results

	RT	Area	% Area
1	8.302	11162	0.07
2	9.123	8228	0.05
3	11.354	7115	0.04
4	11.974	7084	0.04
5	13.445	16191623	98.51
6	14.082	107155	0.65
7	15.399	26533	0.16
8	16.560	66586	0.41
9	19.011	11701	0.07

Figure. S4. HPLC spectra of pyrazoline derivatives (3a-3l).

3a

Analysis Info

Analysis Name	Y:\Data\MARCH 2022\KM 3A000002.d	Acquisition Date	03/15/22 3:25:37 PM
Method	tune_low.m	Operator	BDAL@DE
Sample Name	tm 1 100	Instrument	micrOTOF-Q 228888.10262
Comment			

Acquisition Parameter

Source Type	ESI	Ion Polarity	Positive	Set Nebulizer	0.4 Bar
Focus	Active	Set Capillary	4500 V	Set Dry Heater	180 °C
Scan Begin	50 m/z	Set End Plate Offset	-500 V	Set Dry Gas	4.0 l/min
Scan End	2800 m/z	Set Collision Cell RF	150.0 Vpp	Set Divert Valve	Waste

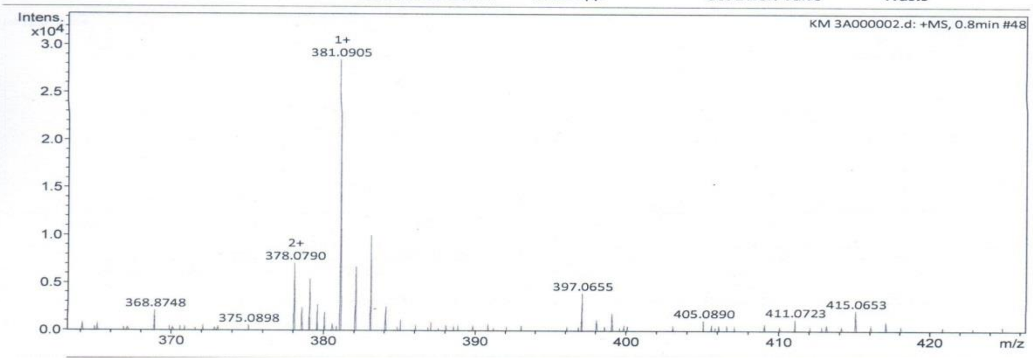

3h

Analysis Info

Analysis Name	Y:\Data\MARCH 2022\KM 3H000002.d	Acquisition Date	03/15/22 3:14:00 PM
Method	Tune_Low_New.m	Operator	BDAL@DE
Sample Name	tm 1 100	Instrument	micrOTOF-Q 228888.10262
Comment			

Acquisition Parameter

Source Type	ESI	Ion Polarity	Positive	Set Nebulizer	0.4 Bar
Focus	Active	Set Capillary	4500 V	Set Dry Heater	180 °C
Scan Begin	50 m/z	Set End Plate Offset	-500 V	Set Dry Gas	4.0 l/min
Scan End	3000 m/z	Set Collision Cell RF	250.0 Vpp	Set Divert Valve	Source

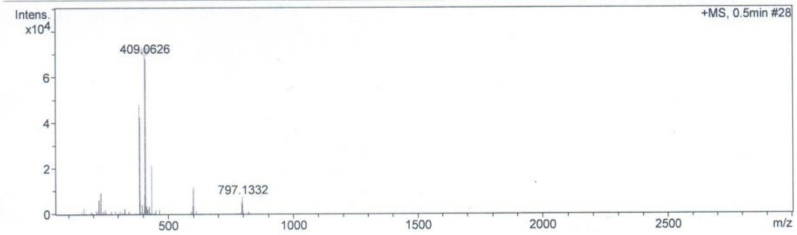

Meas. m/z	#	Ion Formula	m/z	err [ppm]	mSigma	# mSigma	Score	rdb	e⁻ Conf	N-Rule
409.0626	1	C18H19BrN4NaO	409.0634	2.1	13.6	1	100.00	10.5	even	ok

Figure. S5. HRMS spectra of lead compounds (3a and 3h).
